# Physiological characteristics and transcriptomic analyses of alfalfa root crown in wintering

**DOI:** 10.3389/fpls.2024.1486564

**Published:** 2024-12-09

**Authors:** Xiaolong Wang, Hua Chai, Shasha Li, Yanxia Xu, Yue Wu, Jianli Wang, Zhao Yang

**Affiliations:** ^1^ Branch of Animal Husbandry and Veterinary of Heilongjiang Academy of Agricultural Sciences, Qiqihar, Heilongjiang, China; ^2^ Institute of Grass Science of Heilongjiang Academy of Agricultural Sciences, Harbin, Heilongjiang, China

**Keywords:** alfalfa, cold resistance, physiological characteristics, RNA sequencing (RNA-seq), differentially expressed genes (DEGs), winter survival rate

## Abstract

**Background:**

Alfalfa, scientifically identified as *Medicago sativa*, is repeatedly referred to as the “king of forages”. Because of its tight relationship to winter hardiness, the alfalfa’s root crown plays a significant role as a storage organ over the winter. At present, it is still unknown what molecular process makes the alfalfa root crown resistant to cold. This study was aimed to study these knowledge gaps. Using RNA sequencing (RNA-Seq) technology, significant genes associated with cold hardiness were found.

**Methods:**

According to the random block design, Longmu 806 alfalfa and Sardi alfalfa were planted in regional experiments. Under the condition of low-temperature treatment in winter, the differentially expressed genes (DEGs), winter survival rate (WSR), and physiological characteristics were, in turn, calculated by RNA-Seq, chemical analysis, and field investigation.

**Results:**

The WSR of the Longmu 806 alfalfa was 3.68-fold greater than that of the Sardi alfalfa. The jasmonic acid (JA), soluble sugar (SS), proline (Pro), and glutathione (GSH) concentration in the roots of Longmu 806 alfalfa was more than the same amount in Sardi alfalfa in other words P is less than 0.05. An entire set of 878 DEGs related to winter hardiness was found by statistical analysis. Among them, 463 DEGs showed an increase in expression, whereas 415 DEGs showed a decrease in expression. The metabolic pathways’ examination presented that the DEGs (*MsERF1, MsCHIB, MsJAZ, MsAOC, MsGST, MsINV*, *MsTPS*, and *MsOAT*) were linked to the pathways of “plant hormone signaling transduction”, “Amino sugar and nucleotide sugar metabolism”, and “glutathione metabolism”. Furthermore, the physiological changes in JA, SS, Pro content, and GSH were influenced by the dynamic transcription profile of LT (low- temperature) resistance-related genes.

## 1 Introduction

Alfalfa frequently named the “king of forages”, is a highly popular perennial legume forage. It is widely recognized for its exceptional productivity and abundant nutritional value (Wang et al., 2021). Alfalfa is known as the most frequently produced and widely distributed forage globally in China (Shi et al., 2017; Liu et al., 2021). With the abnormal global climate change, extreme weather such as sudden cooling and cold waves occur frequently in local areas. Low temperatures will not only affect the growth and development of alfalfa but also affect the overwintering and yield of alfalfa, so cold-resistant varieties are very scarce in cold areas. In recent years, although some alfalfa varieties sourced domestically and internationally offer high quality and yield, their low winter survival rates significantly hinder the industrial development of alfalfa in northern China (Seppänen et al., 2018; Wang et al., 2021). The farming of alfalfa diversities that could not carefully endure harsh winter situations poses problems not only in northern China but also in midwestern Canada, the United States, Russia, Italy, and other countries (Castonguay et al., 2010). The deficiency of winter-hardy alfalfa diversities in several cold districts makes it crucial for breeders to know the molecular and physiological instruments of alfalfa’s resistance to cold stress, aiming to enhance its yield.

During winter, plants can ensure their survival and bolster freezing resistance through a cold adaptation process. Previous research has highlighted that plant responses to low temperatures primarily involve plant physiology, phytohormones, and the upregulation of cold-induced gene expression (GE) via the regulation of transcription factors (TFs) (Zhuo et al., 2018), comprising alters in the features of the cryoprotectant molecules’ synthesis, for instance, soluble sugars and proline, and a surge in the glutathione (GSH). During the plant’s reaction to cold stress, the main physiological functions of GSH are scavenging free radicals and anti-oxidation. GSH can eliminate free radicals and play a strong protective role on plant cells. Due to the structure of glutathione, GSH contains a reactive sulfhydryl -SH that is easily dehydrogenated by oxidation, this special structure makes it the main free radical scavenger in the plant ROS (reactive oxygen species). During the plant’s reaction to cold stress, detrimental factors namely, malondialdehyde and reactive oxygen species accumulate within plant cells. These harmful substances can inflict damage to cell membranes, proteins, and biological macromolecules, disrupting the organism’s homeostasis and resulting in significant harm to the plants.

Plants experience several changes when exposed to low temperatures. A crucial reaction involves activating molecular networks that are connected to pathways for transmitting stress signals. This activation also leads to the production of certain groups of genes relevant to stress (Chinnusamy et al., 2004). Currently, the broadly investigated signaling pathway is the CBF (C-repeat-binding factor) pathway. *CBF* transcription factors (TFs) control the explanation of *COR* (cold-responsive) functional genes, being essential parts of the LT signal transduction system. This regulatory mechanism ultimately enhances the plants’ cold tolerance (Tayeh et al., 2013; Zhu, 2016). Prior research has demonstrated that the CBF signaling system can enhance the cold resistance of plants, specifically *Arabidopsis thaliana* (Park et al., 2018; Wang et al., 2022), *Medicago sativa* (Kanchupati et al., 2017; Shu et al., 2017), *Triticum aestivum* (Guo et al., 2019), and *Glycine max* (Robison et al., 2019). The CBF signaling pathway likely has a substantial impact in bolstering alfalfa’s resistance to colds. Meanwhile, the plant hormone signaling system is crucial during the plant’s reaction to LT stress and regulates both the CBF-independent and CBF-dependent pathways (Eremina et al., 2016). Among them, JA (jasmonic acid) is a vital hormone that regulates abiotic stress. Nevertheless, new research has designated that JA can influence the expression of the allene oxide cyclase (*AOC*) genes through the regulation of *CBF* transcription, and potentially serving a main function in mitigating LT stress in plants (Yang et al., 2023).

The exact roles of JA in reaction to LT stress will be clarified in the future. In addition, Functional genes, including those in the AP2/ERF families, have a significant role in safeguarding plants from freezing and cold. ERFs (ethylene-responsive factors) are members of the AP2/ERF superfamily of transcription features and have a significant impact on plant reactions to various environmental stressors. The *MfERF* gene, which is sensitive to cold temperatures, was obtained from *Medicago falcata*, a significant forage legume known for its high cold tolerance. Transgenic plants that had an excessive amount of *MfERF* showed enhanced resistance to freezing and chilling (Zhang and Huang, 2010; Ma et al., 2014; Zhuo et al., 2018). These genes act as crucial regulators of plant reactions to LT stress.

RNA sequencing (RNA-Seq) is now vital for functional gene explanation, identifying different genes, analyzing GE differences, and conducting molecular marker investigations. RNA-Seq allows for the investigation of GE at the level of transcription and reveals genes that are engaged in biological processes particular to plants (Gailing et al., 2017; Qi et al., 2022). RNA-Seq is utilized to identify LT stress reaction genes in seedlings, taproots, and alfalfa’s crown buds (Song et al., 2016; Zhuo et al., 2018; Liu et al., 2019). Thus, to boost the finding of the extensive genetic regulation apparatus governing alfalfa’s resistance to low temperatures, the explanations patterns of genes reacting to low temperatures in the alfalfa root crown were specifically examined. The alfalfa root crown is a crucial storage organ, particularly throughout the wintering phase, as it plays a significant role in winter hardiness. When the root crown is not adapted to climate change, temperatures below 0°C could be a reason for damaging the early spring’s roots, which can cause plant death in severe LT stress. However, the Longmu 806 alfalfa variety is capable of withstanding winter temperatures as low as -35°C without any harm. At present, the specific molecular processes that contribute to the ability of the alfalfa root crown to withstand low temperatures throughout the winter are not yet understood. Conducting a systematic examination of the Longmu 806 alfalfa’s resistance to low temperatures during overwintering is beneficial for understanding the alfalfa root crown’s response mechanism throughout this period. Additionally, it serves as a valuable reference point for functional genomics studies on LT stress in alfalfa.

## 2 Materials and methods

### 2.1 Plant material and sample collection

2 tetraploid varieties of *Medicago sativa* were chosen: “Longmu 806” alfalfa (fall dormancy, FD = 1.0), known for its high winter hardiness, and “Sardi” alfalfa, whose FD is equal to 7.0, lacking winter hardiness. Alfalfa seeds were acquired from the Veterinary of Heilongjiang Academy of Agricultural Sciences and Branch of Animal Husbandry. To maintain consistency and reduce uncontrolled stress factors, the two varieties were first cultivated in a greenhouse adjacent to the Research areas. On April 6th, seeds of alfalfa were planted in cultivation pots with certain dimensions that are height: 17.5 cm, top diameter: 8.0 cm, and bottom diameter: 5.5 cm. The pots have been stuffed with a soil substrate that included a high concentration of nutrients, which was supplied by Worth Production Base in Shanghai, China. Every pot was filled with 5 seeds, resulting in a total of 300 pots for each variety. Every set of seeds comprised 300 plants, with a total of three replications. After 2 weeks of propagation, the seedlings were manually thinned to 3 plants per container. The plants were irrigated at a frequency of once every 3 days. The greenhouse temperature was carefully regulated at 25°C, creating an optimal environment for the growth of seedlings. These seedlings were nurtured under natural daylight hours, receiving a generous 14 hours of daylight each day. The weeds were carefully and methodically eliminated by hand.

The alfalfa’s 2 diversities were moved to the search area placed in Hohhot, Inner Mongolia, China (111°58’E, 40°39’N) after a period of a month and a half of rising in the greenhouse. Every plot measured 6.0 meters in length and 4.0 meters in width, with a row spacing of 0.4 meters. Each row consisted of 30 plants, spaced 0.2 meters apart. The examination plots were arranged in a stochastic full-block design by 3 independent experiments. The Caoyuan No. 3 variety of alfalfa was grown around the allotment as a protective barrier. The soil consisted primarily of chestnut soil and had a composition of 15.97 g kg^-1^ of OM (organic matter) at a depth of 0-30 cm. It also contained 348.0 mg kg^-1^ of AN (available nitrogen), 54.0 mg kg^-1^ of AP (available phosphorus), and 328.0 mg kg^-1^ of AK (available potassium). The soil’s pH, measured at a ratio of 1:2.5 in water, was 8.30.

The root crown of the Longmu 806 and Sardi alfalfa cultivars were obtained from plants that were 1 year old. The root crown was excavated from plants that were randomly selected from the test plot. The root crown was rinsed with filtered water, thereafter isolated, and promptly cryopreserved in liquid nitrogen at -80°C for subsequent RNA extraction. The levels of soluble sugar (Buysse and Merckx, 1993), free proline (Bates et al., 1973), GSH (Griffith, 1980), and JA (Šimura et al., 2018) contents were measured. The root crown was washed with purified water, then separated and immediately frozen in liquid nitrogen at -80°C for later production of RNA.

The statistical study was conducted utilizing SAS 9.0 software (North Carolina State, SAS Institute Inc, USA), employing an ANOVA trial. The two groups of sample data conform to the normal distribution of Student’s T-test. Main modifications were determined utilizing the Duncan multiple ranges assessment at a level of significance of P less than 0.05.

### 2.2 RNA-Seq library construction

The RNA Prep Pure Plant Kit (Tiangen, Beijing, China) was utilized to take out the whole RNA from 6 samples, every consisting of 3 biological duplicates of *Medicago sativa* varieties. The extraction process followed the manufacturer’s procedure. The RNA samples were evaluated utilizing an Agilent 2100 Bioanalyzer (USA, Agilent Technologies, Santa Clara, CA), and their amount was specified utilizing a NanoDrop-2000 (USA, Wilmington, Thermo Scientific, DE). The mRNA was extracted utilizing the poly (A) selecting technique, employing oligo (dT) beads. Subsequently, it was disintegrated utilizing a fragmentation buffer. Subsequently, the mRNA that had been enhanced was transformed into a cDNA (complementary DNA) library using the double-stranded cDNA synthesis kit Superscript (USA, CA, Invitrogen, Carlsbad) and stochastic primers (Illumina), using the instructions provided by the manufacturer. The libraries of cDNA were generated utilizing the Tru-Seq RNA Sample Prep Kit (Illumina) following the guidelines provided by the manufacturer, with a total of fifteen PCR cycles. The libraries were chosen using two percent ultra-low range agarose (USA, Bio-Rad, CA, Hercules). The bands of interest were chosen based on their size utilizing low-range ultra-agarose and their quantity was measured utilizing a TBS 380 fluorometer (Invitrogen) and Pico Green Assay (USA, Carlsbad, CA, Life Technologies).

### 2.3 Illumina deep sequencing

Shanghai Majorbio Bio-pharm Biotechnology Co., Ltd. in Shanghai, China received the alfalfa specimens. Utilizing an Illumina HiSeqTM 4000 system with 200 cycles, transcriptome sequencing was carried out, yielding a read length of 2 × 150 bp. The data from the sequencing of the transcriptome were archived in the NCBI/SRA (National Center for Biotechnology Information Short Read Archive) database.

### 2.4 *De novo* transcriptome gathering and unigene discovery

Due to the lack of prior alfalfa genome data, the clean data obtained from the alfalfa specimens were reconstructed from scratch utilizing the Trinity (V.2.4) software and reference genome (http://trinityrnaseq.sf.net) (Grabherr et al., 2011). Trinity is a software bundle comprising 3 components: Butterfly, Inchworm, and Chrysalis. Initially, Inchworm divides the reads into smaller fragments, constructs a dictionary of k-mers (k=25), chooses the k-mers from the clear readings, and elongates both ends to create contigs. Next, Chrysalis fragments combine and cluster with overlapping contigs to create parts. Every part represents a collection of potential classifications for homologous genes or variable shear isoforms. A de Bruijn graph is constructed for every part. Butterfly streamlines the de Bruijn graph for every part, produces the variable splicing subtype’s complete transcript, merges the homologous genes transcripts, and ultimately produces the splicing consequence file.

### 2.5 Unigene annotation and DEGs analyses

The unigenes have been annotated by aligning them with the BLASTX algorithm against the NCBI Nr database (https://www.ncbi.nlm.nih.gov/public/, July 2023), the GO database (http://www.geneontology.org, July 2023), and the KEGG database (http://www.genome.jp/kegg/, July 2023), using an E-value threshold of 1 × 10^-5^. Protein functional annotations were acquired based on the most accurate alignment outcomes. The study of GE of unigenes was computed and standardized using the FPKM reads (fragments per kilobase per million) method. The DEGs were examined utilizing the DESeq2 program. Following P-value threshold testing utilizing the false discovery rate (FDR), genes with an FDR value less than or equal to 0.05 and a fold alteration greater than 1 (in absolute value) were chosen as thresholds to choose the DEGs. The DEGs underwent additional analysis to determine their enrichment in Gene Ontology (GO) terms and Kyoto Encyclopedia of Genes and Genomes (KEGG) pathways.

### 2.6 Winter survival and physiological characteristics

**Figure 1:**
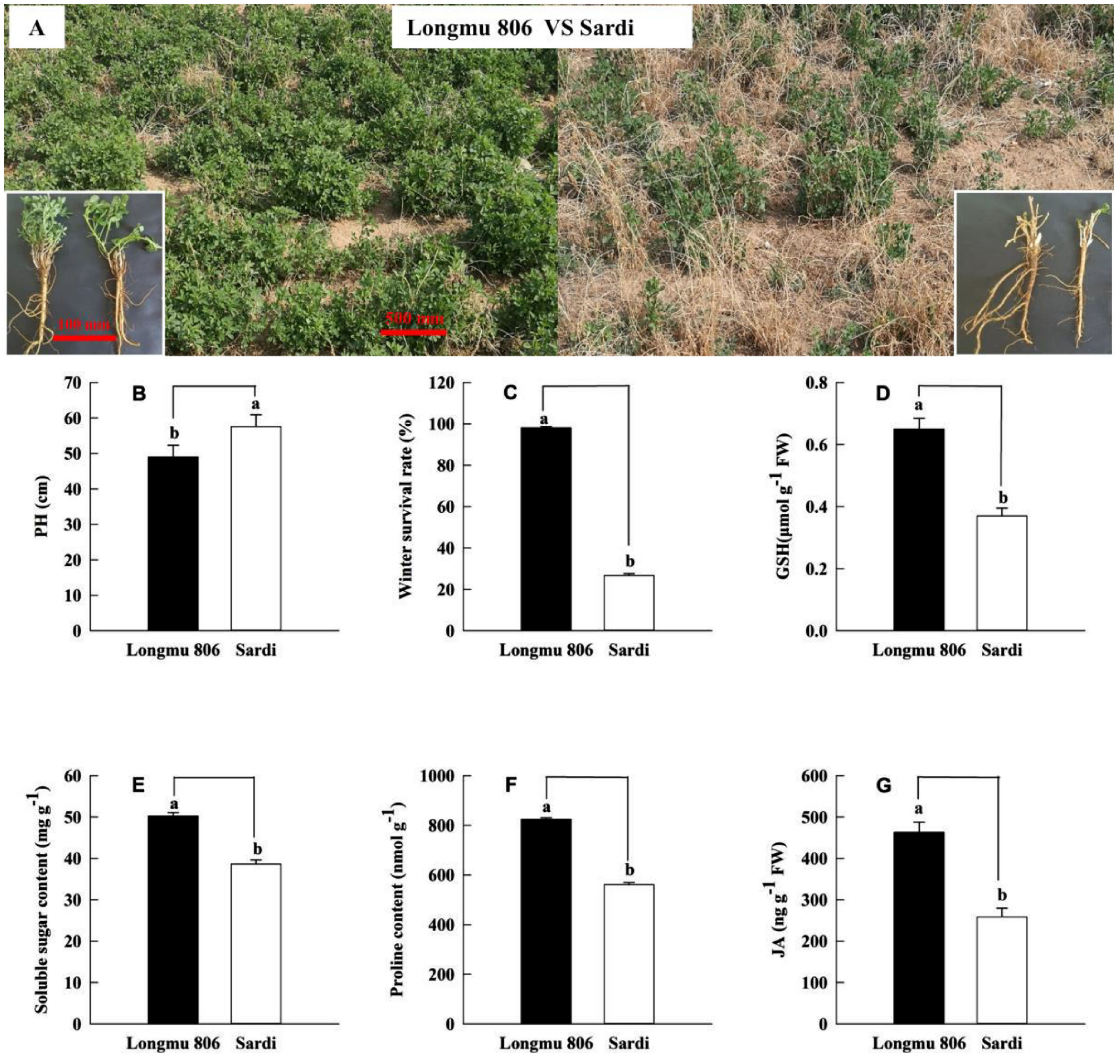
An analysis of physiological indexes and morphological characteristics among Longmu 806 and Sardi alfalfa. The winter survival investigation **(A)**, plant height (PH) **(B)**, winter survival rate **(C)**, GSH **(D)**, soluble sugar **(E)**, proline **(F)**, and JA **(G)** of Longmu 806 and Sardi. Longmu 806 alfalfa exhibits winter endurance, whereas Sardi alfalfa lacks winter hardiness. The information is displayed as the mean value plus or minus the standard error. Lowercase letters signify statistically important differences at the 0.05 probability level between the 2 kinds.

During the spring season, Longmu 806 alfalfa revealed more regrowing vegetation in comparison with Sardi alfalfa (**Figure 1A**). The Sardi alfalfa PH (plant height) was substantially greater than that of Longmu 806 alfalfa, precisely P is less than 0.05 (**Figure 1B**). The Longmu 806 alfalfa’s winter survival rate (98.09%) was significantly higher compared to Sardi alfalfa (P < 0.05, **Figure 1C**), showing a 3.68-fold increase. The GSH concentration in the roots of Longmu 806 alfalfa was 1.74-fold more than the same amount in Sardi alfalfa in other words P is less than 0.05 (**Figure 1D**). In Longmu 806 alfalfa roots, the amounts of soluble sugar, proline, and JA were considerably greater compared to Sardi alfalfa (P < 0.05, **Figures 1E–G**).

### 2.7 *De novo* transcriptome assembly

**Figure 2:**
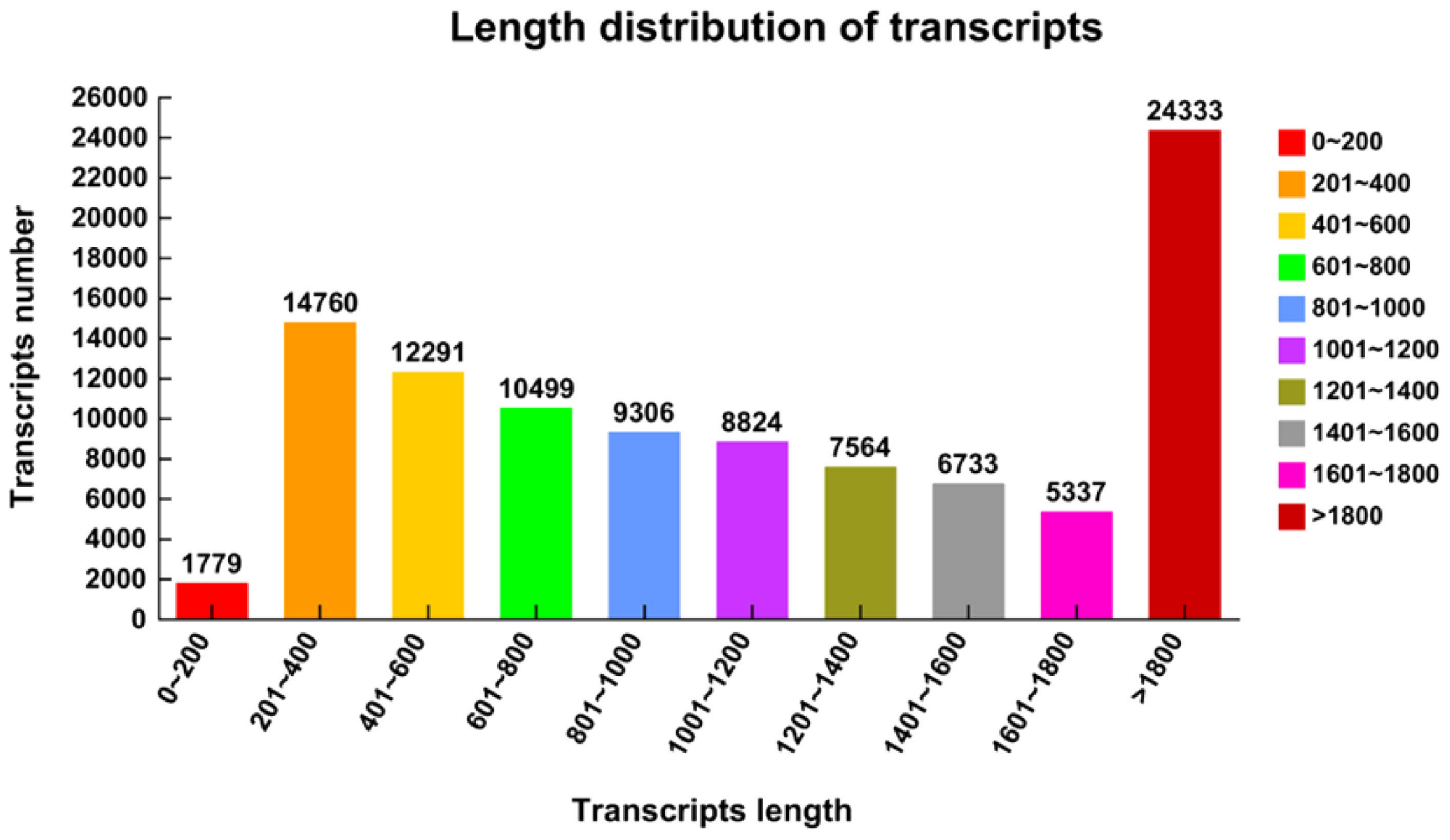
Length distribution of *Medicago sativa* transcripts. The abscissa represents the transcripts’ length of the *Medicago sativa*, ranging from 0 transcripts to more than 1800. The ordinate represents the number of transcripts of *Medicago sativa*.

Six libraries were created for high-throughput sequencing using whole RNA isolated from the Longmu 806 and Sardi root crowns cultivars throughout the wintering phase. To ensure the accuracy and dependability of the data examination, the main dataset underwent a process of filtration. Raw readings were filtered by eliminating inferior reads, reads with adaptor sequences, and reads with inferior bases. The reference genome was Zhongmu No 1. A whole 44,165 genes were detected, including 32,513 identified genes and 11,652 new genes. The total number of expressed transcripts was 73,536, consisting of 33,349 known transcripts and 40,187 new transcripts. A total of 101,426 transcripts were acquired, with a maximum length of 1,779 (ranging from 0 to 200) and a minimum length of 14,760 (ranging from 201 to 400) (**Figure 2**).

### 2.8 Functional annotation

**Figure 3:**
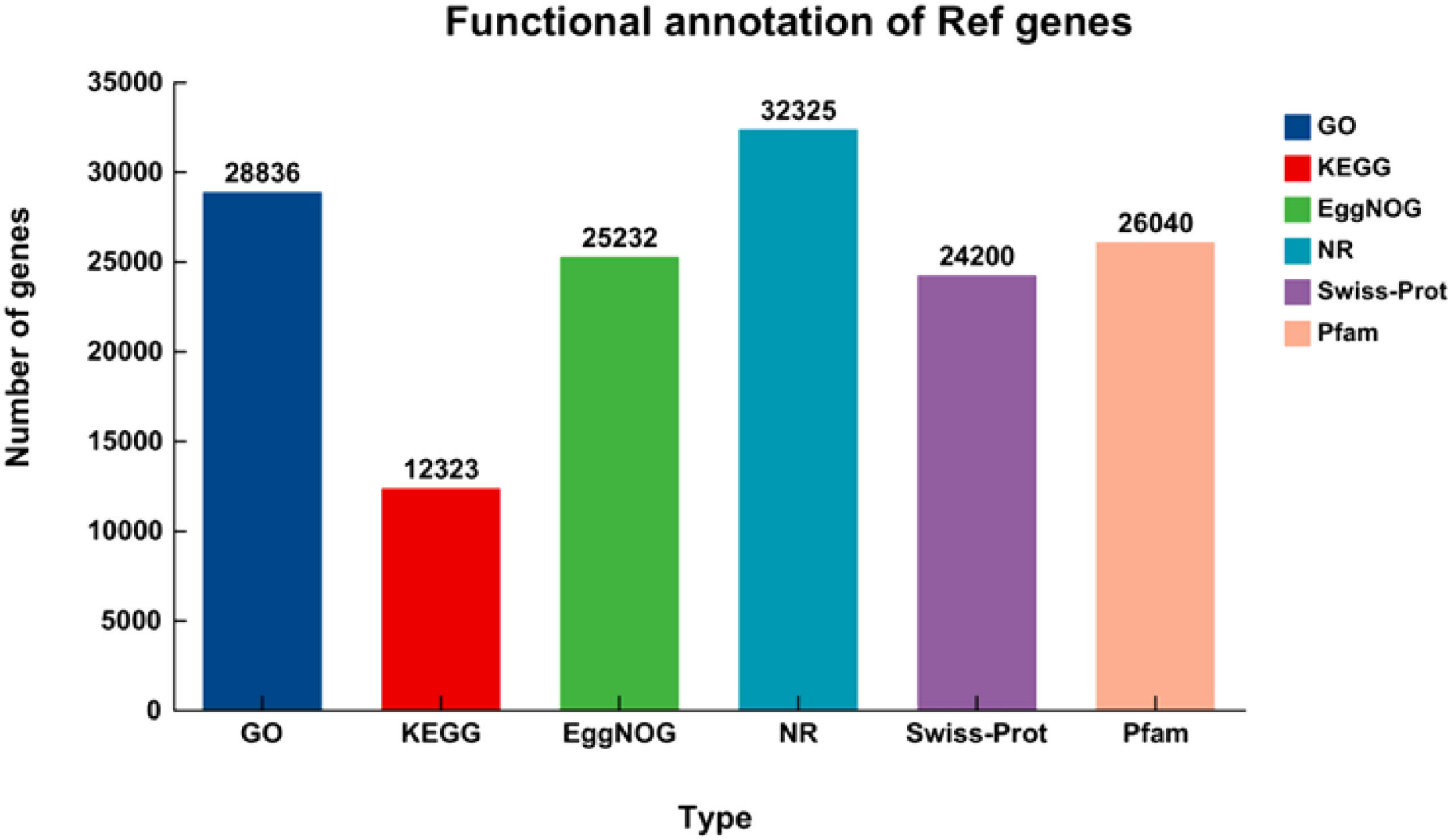
The number of genes that have been explained by BLASTX using an E-value less than 1 × 10^-5^ in the databases. The numerical values displayed within the bars represent the overall count of genes that have been annotated in the databases.

A total of 32,513 unigenes, which account for 73.61% of the dataset, were annotated using BLASTX against several protein databases including KEGG, Pfam, EggNOG, Nr, SwissProt, and GO. The annotation was performed with a stringent E-value threshold of less than 1 × 10^-5^. Out of the unigenes, 99.42% (32,325), 74.43% (24,200), 80.09% (26,040), 77.61% (25,232), 88.69% (28,836), and 37.90% (12,323) were, in turn, marked up to the SwissProt, Nr, EggNOG, Pfam, KEGG, and GO databases (**Figure 3**).

### 2.9 Analyses of differentially expressed genes

**Figure 4:**
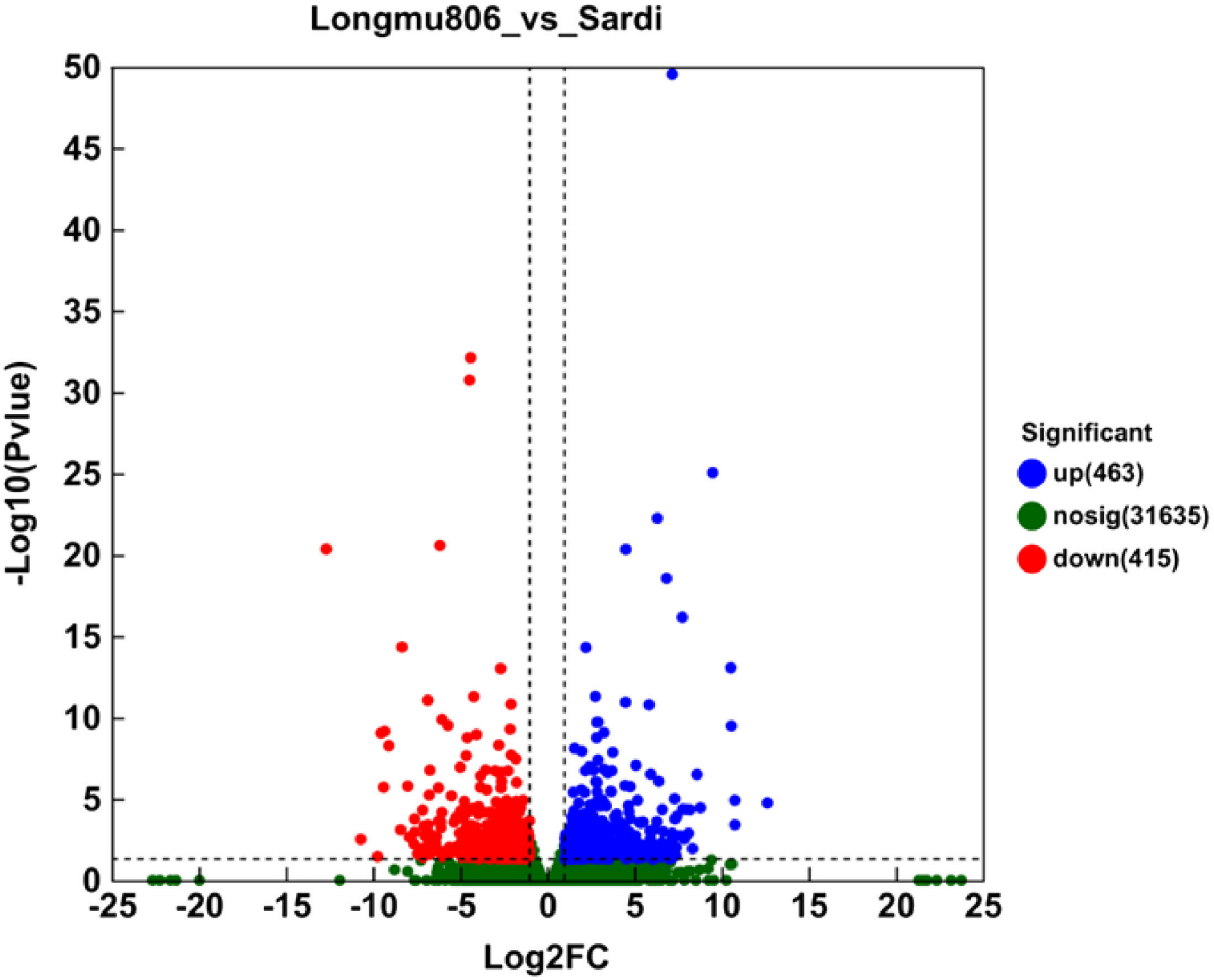
The Longmu 806 and Sardi *Medicago sativa* varieties have distinct sets of differentially expressed genes. The DEGs were selected by utilizing an FDR (false discovery rate) threshold of < $\frac{5}{100}$ and a threshold of |Log2 fold change| is more than 1. The volcano plot displayed 878 DEGs, consisting of 463 elevated unigenes and 415 reduced unigenes. Green spots designate DEGs that are downregulated, whereas red spots represent DEGs that are upregulated. The black-colored unigenes exhibited negligible expression levels.

The Nr annotation findings were utilized to construct GO functional annotations of unigenes using BLAST2GO (2.5) software. A full 878 DEGs were considered into any number of ontologies based on the criteria of |log2 fold change| that is more than 1 and P-value is less than 0.05 (Extra file 1: **Supplementary Table S1**; **Figure 4**). There was a total of 463 unigenes that showed an increase in activity, whereas the remaining 415 unigenes showed a decrease in activity when comparing Longmu 806 to Sardi. For the following studies, the DEGs were utilized.

**Figure 5:**
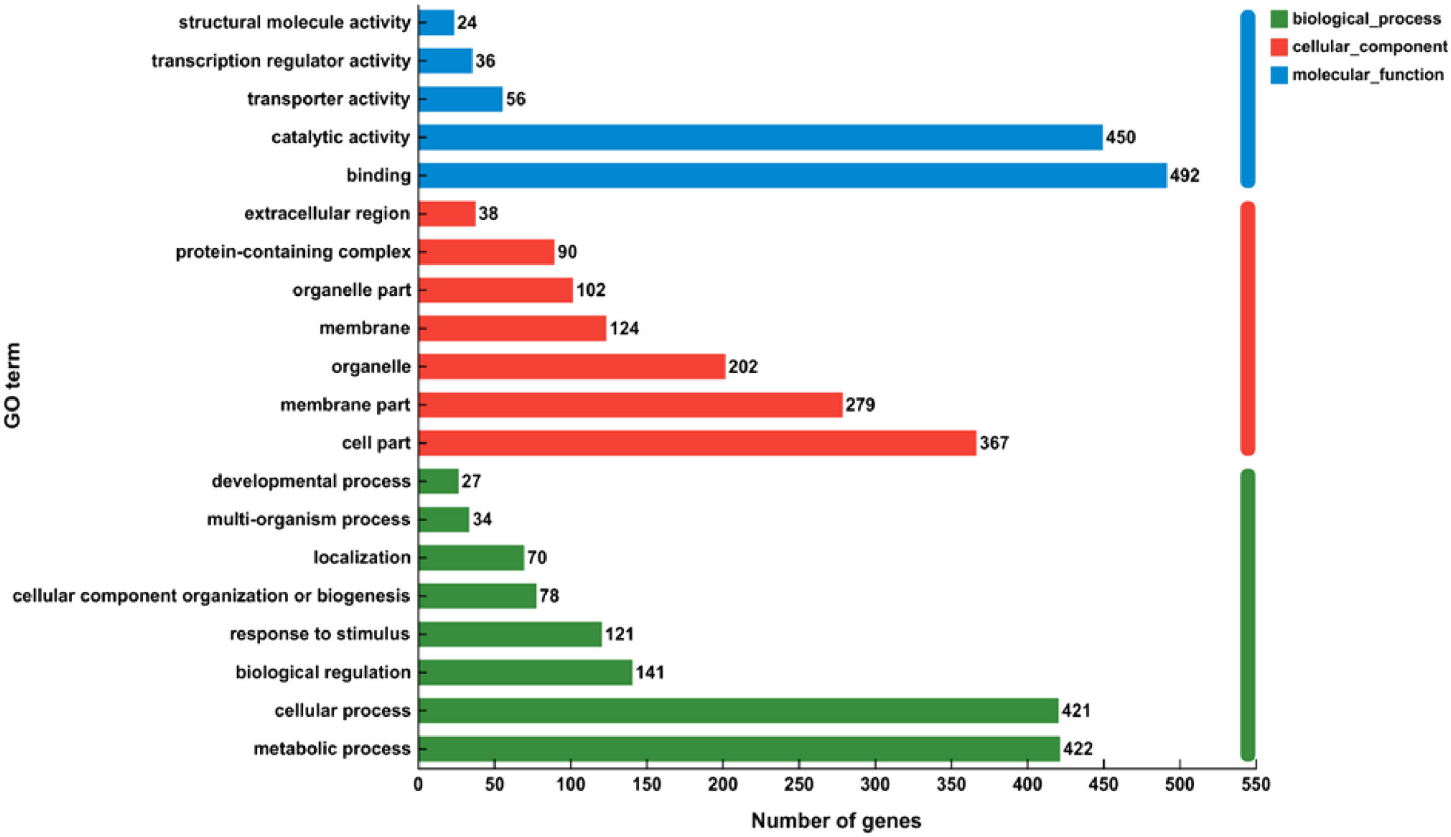
Performing GO (Gene Ontology) explanation examination on the collected unigenes. There were a total of 878 genes exposed to differential expression. These genes were divided into 3 primary categories: molecular function, cellular component, and biological process.

To gain a deeper comprehension of the impact of genes uttered differently in alfalfa during the winter season, GO functions were employed to categorize these DEGs’ activities (Extra file 2: **Supplementary Table S2**). There were a whole 878 DEGs classified into 3 primary functional sorts: molecular function, cellular component, and biological process (**Figure 5**). Within the biological processes’ domain, the phrases “metabolic process” (GO: 0008152), “cellular process” (GO: 0009987), and “biological regulation” (GO: 0065007) were frequently seen. These terms encompassed 422,421, and 141 unigenes, respectively. Within the cellular component sort, the majority of genes were categorized as “cell part” (367 unigenes), “membrane part” (279 unigenes), and “organelle” (202 unigenes). Within the molecular function category, the numerous genes were found to be highly concentrated in the subcategories of “binding” (492 unigenes) and “catalytic activity” (450 unigenes).

### 2.10 KEGG pathway analyses

**Figure 6:**
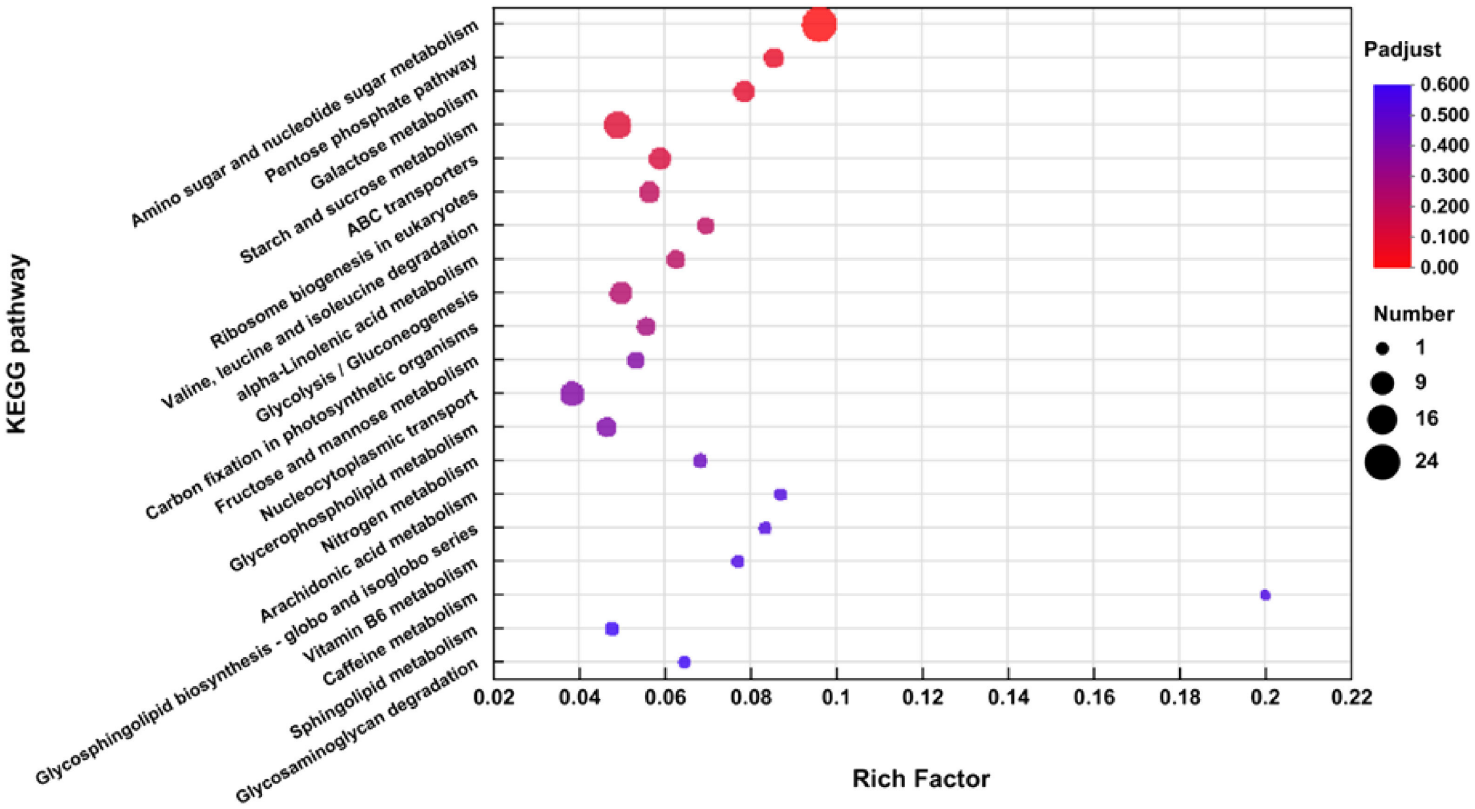
The DEGs were subjected to KEGG pathway analyses.

The DEGs’ KEGG analysis was conducted with KOBAS (2.1). A total of 333 DEGs were categorized into 87 KEGG pathways. The details may be seen in Extra File 3: **Supplementary Table S3**; **Figure 6**. The KEGG pathways with the maximum depiction of DEGs include “Amino sugar and nucleotide sugar metabolism” (map 00520), “Pentose phosphate pathway” (map 00030), “Galactose metabolism” (map 00052), “Starch and sucrose metabolism” (map 00500), “ABC transporters” (map 02010), “Valine, leucine and isoleucine degradation” (map 00280), “Ribosome biogenesis in eukaryotes” (map 03008), “Protein processing in endoplasmic reticulum” (map 04141), “Plant-pathogen interaction” (map 04626), “Nucleocytoplasmic transport” (map 03013), “alpha-Linolenic acid metabolism” (map 00592), “Plant hormone signal transduction” (map 04075), and “Glycolysis/gluconeogenesis” (map 00010).

In the pathway of “plant hormone signal transduction”, DEGs linked to ethylene’s production (*MsERF1*, *MsCHIB*) were upregulated, whereas genes involved in the manufacture of jasmonic acid (*MsAOC, MsJAZ*) were downregulated.

**Figure 7:**
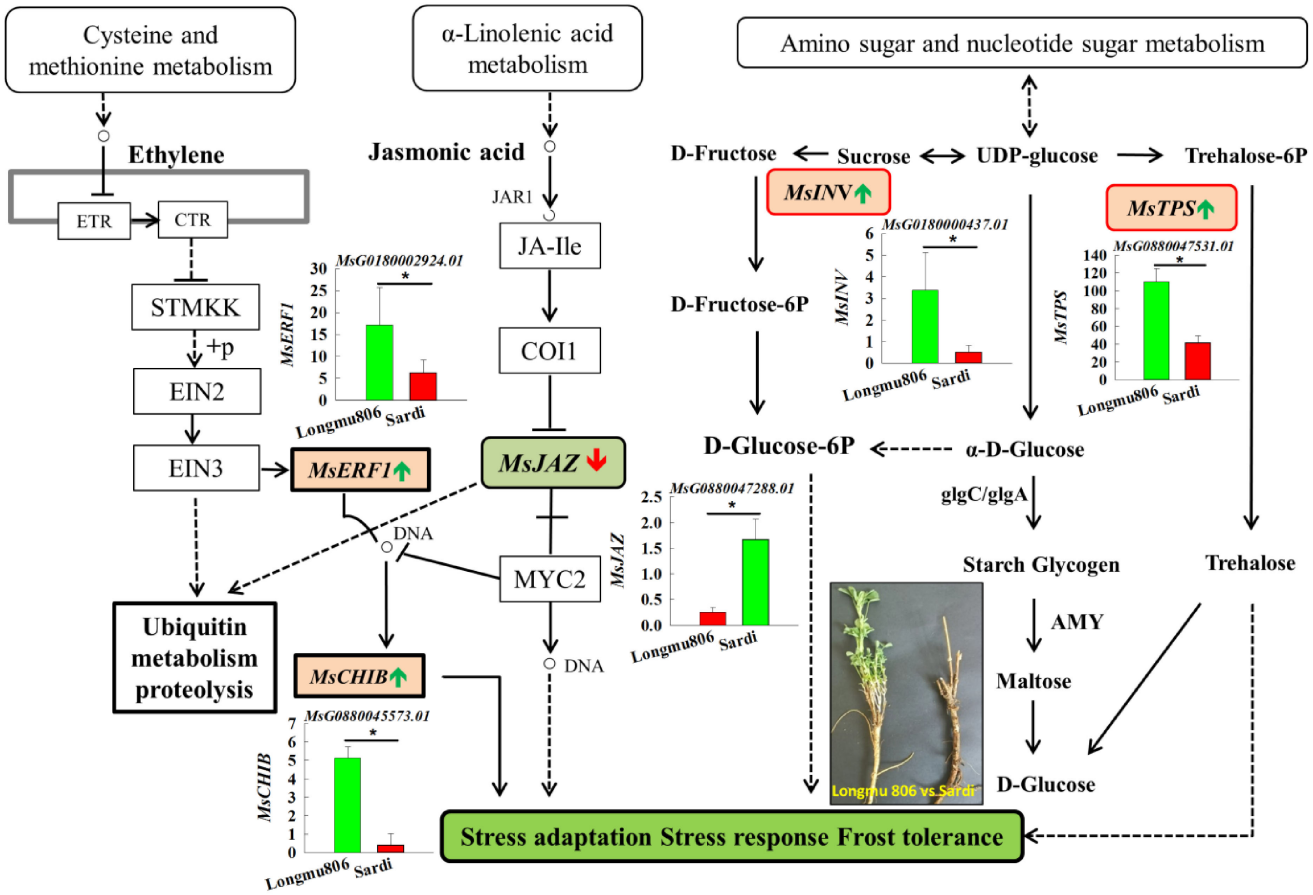
Performing KEGG studies on the pathways of “plant hormone signal transduction” and “Amino sugar and nucleotide sugar metabolism”. Green bars signify genes that have been upregulated, or increased in expression, while red bars indicate genes that have been downregulated, or decreased in expression. An asterisk indicates a statistically significant difference between the two varieties at the 0.05 probability level.

In the “Glutathione metabolism” pathway, DEGs connected to superoxide dismutase (*MsGST*) were downregulated. In the “Amino sugar and nucleotide sugar metabolism” pathway, DEGs associated with glucose, fructose, and trehalose (*MsINV, MsTPS*) were upregulated (**Figure 7**). The annotations were useful tools for investigating the precise activities and pathways of the alfalfa genes.

## 3 Discussion

### 3.1 Hormones in response to low-temperature stress

LT stress is a primary abiotic stress significantly impacting plant growth and the production of alfalfa (Seppänen et al., 2018). Typically, freezing damage at low temperatures is more likely to happen under harsh winter conditions, which can lead to decreased alfalfa yields in the subsequent year. Hence, the capacity for regrowth during the early spring after harsh winter circumstances is indicative of the level of cold resistance in forage plants (Yu et al., 2015). The Longmu 806 alfalfa’s winter survival rate was markedly superior to that of Sardi alfalfa, indicating that fall-dormant alfalfa exhibits greater winter hardiness. Additional research has also indicated a positive correlation between winter survival and fall dormancy (Li et al., 2015; Liu et al., 2021). The alfalfa variety’s winter hardiness is impacted by multiple features, including the growth environment, as well as the physiology and hormone regulation of the variety (Castonguay et al., 2013).

Hormones serve as the catalysts for the winter hardiness of GE’s activation, and the responses to abiotic stress caused by hormones entail many processes. ABA, ethylene, and JA are phytohormones that have significant roles in regulating plant development in perennial plants to help them adapt to LT stress (Kazan, 2015). Ethylene signaling stimulates several ethylene response factor (*ERF, CHIB*) genes’ transcriptions, eventually directing plant development and responses to abiotic factors (Bi et al., 2024; Zaman et al., 2023). The *MfERF1* gene is derived from *Medicago falcata*, and it can significantly improve the cold tolerance of alfalfa (Ma et al., 2014; Zhuo et al., 2018). The *MfERF1*’s GE was markedly increased in response to LT stress in the Longmu 806 alfalfa. Alike findings were observed in *Medicago truncatula* (Shu et al., 2016). The AP2/ERF transcription factor family, namely the *MtERF* genes (also known as *MtCBF* genes), is located on chromosome 6 in *Medicago truncatula* (Shu et al., 2016). This gene family plays a vital role in the plant’s reaction to freezing and cold conditions. This finding confirms earlier studies in Camellia sinensis showing five *CsAP2/ERF* genes from each AP2/ERF family (ERF subfamilies) exhibited a response to temperature stressors (Wu et al., 2015). Essentially, these outcomes validated that *ERF1* and *CHIB* genes exhibit a favorable reaction to LT stress (Bi et al., 2024; Zaman et al., 2023). The Longmu 806 cultivar exhibited upregulation of the *MsERF1* and *MsCHIB* genes, indicating an improvement in cold resistance. Multiple studies have documented that JA influences enhancing plant resilience to LT stress. Furthermore, an increase in JA levels effectively controls the ICE-CBF transcriptional pathway, leading to improved cold resistance (Hu et al., 2017). The RNA-Seq investigation of the alfalfa root crown revealed differential expression of DEGs (*MsJAZ, MsAOC*) in reactions to LT stress. The DEGs *MsJAZ* and *MsAOC* in Longmu 806 alfalfa exhibited a substantial decrease compared to Sardi. Longmu 806 alfalfa has the ability to inhibit the production of DEGs *MsJAZ* and *MsAOC* by acting on the ICE-CBF/DREB1 regulatory pathways. Consequently, the release of *CBF* transcription factors induces the explanations of *COR* genes, thus enhancing the cold resistance of alfalfa (Hu et al., 2017; Yang et al., 2023).

The DEGs in response to LT stress elucidated the association between the “plant hormone signal transduction” pathway and winter hardiness. The DEGs related to the production of ethylene (*MsERF1, MsCHIB*) were elevated, whereas the biosynthesis pathways of jasmonic acid (*MsJAZ, MsAOC*) were downregulated. Hormones, acting as signaling molecules, have crucial functions in controlling the expression of the *CBF* gene in *Medicago sativa* in response to cold and freezing conditions (Shu et al., 2017). Longmu 806 alfalfa is more likely to have greater winter hardiness compared to Sardi alfalfa because of its high quantities of accumulated plant hormones.

### 3.2 Osmoregulation and antioxidant defense system in reaction to low-temperature stress

Perennials and winter annuals undergo acclimation throughout winter, during which their metabolism shifts towards the production of cryoprotectants namely, soluble sugar, trehalose, and proline (Ponnu et al., 2011; Song et al., 2016; Zhuo et al., 2018). Throughout periods of LT stress, ROS (reactive oxygen species) could cause damage to cellular components, disrupting the balance within plants and leading to programmed cell death (Suzuki and Mittler, 2006). To safeguard plants from the harmful effects of ROS (reactive oxygen species), plants have developed an intricate physiological mechanism to eliminate ROS (Baxter et al., 2014). One notable non-enzymatic antioxidant is GSH, which has a significant impact on breaking down peroxides and serves as a substrate for some antioxidant enzymes like glutathione S-transferase (GST) (Song et al., 2019).

In their study, Zhao et al. (2015) examined the complete genome GE profile of cold-tolerant rice subjected to low temperatures. They discovered that the *GST* gene was elevated in reaction to LT stress. Contrary to the findings observed in tolerant rice subjected to LT stress. During the overwintering period, the Longmu 806 alfalfa exhibited considerably greater levels of GSH compared to the Sardi alfalfa under freezing stress. Additionally, the expression of the *MsGST* gene (*MsG0180002272.01, MsG0780038937.01*) was downregulated. The KEGG pathway “glutathione metabolism” showed a substantial enrichment, indicating that the *MsGST* gene played a role in removing reactive oxygen species through glutathione during alfalfa overwintering LT stress (Song et al., 2019). The downregulation is likely a result of alfalfa’s unique protective mechanism. The GSH antioxidant system in plants was strengthened during cold acclimation (Baek and Skinner, 2003; Song et al., 2019), as winter temperatures drop, DEGs (*MsGST*) encoding antioxidant enzymes in cold-resistant alfalfa were downregulated to adjust to LT stress in winter. Therefore, Longmu 806 alfalfa could maintain a higher survival rate in cold winters (Song et al., 2016).

The process of accumulating soluble sugars during LT acclimation has a role in stabilizing plasma membranes. Sugars have the ability to safeguard membranes and prevent damage caused by low temperatures. LT adaptation encompasses the alteration of cellular structure and the reconfiguration of GE (Song et al., 2016). Compared to the Sardi alfalfa, the Longmu 806 alfalfa showed a notable increase in soluble sugar content when subjected to LT stress (**Figure 1E**). The KEGG pathway “Amino sugar and nucleotide sugar metabolism” showed a notable increase in enrichment. The transcriptome data showed that the *MsINV* (invertase) gene’s expression level in the Longmu 806 alfalfa was 6.61 times more than that of Sardi alfalfa (**Figure 7**). This discovery is consistent with a prior investigation on *Poncirus trifoliata*, suggesting that the INV enzymes play a role in the irreparable breakdown of Suc (sucrose) into Glc (glucose) and Frc (fructose) (Dahro et al., 2022), and the increased levels of Suc, Glc, Frc, and total sugar in the LT alfalfa were directly associated with the heightened activity of soluble INV enzymes (Dahro et al., 2021). TPS (trehalose-6-phosphate synthase) is crucial for enhancing plant cold tolerance. Initially, TPS catalyzes the transfer of glucose from UDP-glucose to glucose-6-phosphate, resulting in the formation of T6P (trehalose-6-phosphate). Subsequently, T6P undergoes dephosphorylation by T6P phosphatase, resulting in the formation of trehalose (Ponnu et al., 2011). The RNA-Seq data revealed a 2.65-fold upsurge in the activity of the *MsTPS* gene in Longmu 806 alfalfa compared to Sardi alfalfa (**Figure 7**). This suggests that Longmu 806 is more likely to accumulate trehalose, which aligns with prior findings in *Medicago truncatula* (Song et al., 2021). The DEGs (*MsTPS*) were consideringly enhanced in the “starch and sucrose metabolism” pathway, consistent with the KEGG pathway enrichment investigation. The present investigation provides the groundwork for subsequent studies on the *MsTPS* gene in alfalfa and its regulatory function in reaction to LT stress (Song et al., 2021).

Proline can accumulate in conditions of low temperature in plants. It controls the osmotic potential of cells and helps maintain turgor pressure in stressed cells. It also helps plants withstand dehydration, with few or no harmful effects (Zhuo et al., 2018). Furthermore, it was shown that the attention of Pro increased when subjected to cold (Beheshti Rooy et al., 2017). The buildup of Pro in stressful conditions might occur due to increased production or decreased breakdown of Pro. Longmu 806 alfalfa showed a notable increase in Pro content when exposed to LT stress, in contrast to Sardi alfalfa (**Figure 1F**). In addition, the KEGG pathway “arginine and proline metabolism” exposed a considerable upsurge in enrichment. The differentially expressed gene *MsOAT* (ornithine aminotransferase) was upregulated in reaction to LT stress (Fang et al., 2021). The findings indicated that the breakdown of Pro was facilitated by *MsOAT*, and the process of adapting to low temperatures increased the activity of the *MsOAT* gene, leading to the buildup of Pro in Longmu 806 alfalfa.

## 4 Conclusions

This study found 878 differentially expressed genes (DEGs), including *MsERF1, MsCHIB, MsAOC, MsJAZ, MsGST, MsINV*, *MsOAT*, and *MsTPS*. These DEGs are complicated in significant pathways namely “plant hormone signal transduction”, “Amino sugar and nucleotide sugar metabolism”, “arginine and proline metabolism” and “glutathione metabolism”. In addition, the physiological alterations in jasmonic acid, soluble sugar, proline levels, and glutathione were consistent with the dynamic transcription profile of genes relevant to LT resistance. The RNA-Seq information significantly enhanced the alfalfa gene sources, serving as a valuable source for forthcoming comprehensive investigations into the winter hardiness of alfalfa.

## Data Availability

Our transcriptome datasets are available at NCBI project PRJNA558269 with accession number SRP217137, and SRA with accession numbers SRR9888366, SRR9888367, SRR9888364, SRR9888365, SRR9888363, SRR9888362.
