# Supplementary material for: Physiological characteristics and transcriptomic analyses of alfalfa root crown in wintering
Source: Front Plant Sci. 2024 Dec 9;15:1486564. doi: 10.3389/fpls.2024.1486564 (PMC11663680; doi:10.3389/fpls.2024.1486564)
Supplement: Supplementary file 1 [file Table1.docx]

**(TABLE S1) Differential expression genes in the root crown of two alfalfa varieties**

| **Gene_id** | **Log_2_FC**  **(Longmu806/Sardi)** | **P-value** | **Significant** | **Regulate** |
| --- | --- | --- | --- | --- |
| MsG0080047868.01 | 1.149069152 | 0.000889 | yes | up |
| MsG0080047910.01 | 2.18086354 | 0.001533 | yes | up |
| MsG0080047999.01 | 2.503935462 | 0.000194 | yes | up |
| MsG0080048035.01 | 2.653885315 | 0.001171 | yes | up |
| MsG0080048051.01 | -4.576079126 | 0.001056 | yes | down |
| MsG0080048106.01 | 4.241432609 | 2.55E-05 | yes | up |
| MsG0080048202.01 | -1.278946595 | 0.00026 | yes | down |
| MsG0080048322.01 | -2.129999413 | 2.73E-05 | yes | down |
| MsG0080048355.01 | -6.581300657 | 0.000403 | yes | down |
| MsG0080048527.01 | -1.658677086 | 0.000811 | yes | down |
| MsG0080048638.01 | -6.246224119 | 7.07E-09 | yes | down |
| MsG0080048716.01 | -1.871692777 | 0.000139 | yes | down |
| MsG0080048741.01 | 3.052449487 | 1.99E-06 | yes | up |
| MsG0080048805.01 | 1.108667862 | 0.002109 | yes | up |
| MsG0080048824.01 | 7.830943498 | 3.09E-05 | yes | up |
| MsG0080048976.01 | 3.665516643 | 0.001093 | yes | up |
| MsG0080048992.01 | -2.478110756 | 8.62E-06 | yes | down |
| MsG0080049045.01 | -1.809033811 | 0.000969 | yes | down |
| MsG0080049111.01 | 3.299275456 | 0.00211 | yes | up |
| MsG0080049160.01 | -2.110906087 | 0.002017 | yes | down |
| MsG0180000068.01 | 8.32848098 | 0.000272 | yes | up |
| MsG0180000072.01 | -1.374036381 | 0.002165 | yes | down |
| MsG0180000075.01 | -5.241645441 | 0.000188 | yes | down |
| MsG0180000125.01 | 1.126567115 | 0.000763 | yes | up |
| MsG0180000147.01 | 4.662216492 | 0.00103 | yes | up |
| MsG0180000205.01 | -1.439919911 | 0.001471 | yes | down |
| MsG0180000271.01 | 1.51872076 | 2.53E-06 | yes | up |
| MsG0180000284.01 | 1.551065045 | 6.76E-05 | yes | up |
| MsG0180000395.01 | 1.998601858 | 9.61E-05 | yes | up |
| MsG0180000408.01 | 1.131353373 | 0.000224 | yes | up |
| MsG0180000437.01 | 2.689274658 | 0.001042 | yes | up |
| MsG0180000452.01 | 1.53661145 | 2.94E-07 | yes | up |
| MsG0180000472.01 | -3.523390488 | 5.08E-05 | yes | down |
| MsG0180000557.01 | -2.507321463 | 0.000506 | yes | down |
| MsG0180000577.01 | -2.346170221 | 6.77E-05 | yes | down |
| MsG0180000751.01 | 1.161771174 | 0.001992 | yes | up |
| MsG0180000774.01 | -2.191139656 | 0.00028 | yes | down |
| MsG0180000814.01 | 1.631855668 | 2.66E-05 | yes | up |
| MsG0180000842.01 | -1.107853518 | 0.001518 | yes | down |
| MsG0180000936.01 | 5.058570347 | 2.44E-05 | yes | up |
| MsG0180000942.01 | 3.416147999 | 0.000112 | yes | up |
| MsG0180000962.01 | 4.033814947 | 8.48E-05 | yes | up |
| MsG0180000970.01 | 7.239207701 | 0.000303 | yes | up |
| MsG0180000976.01 | 3.248385677 | 1.12E-12 | yes | up |
| MsG0180000984.01 | -2.003656352 | 0.000857 | yes | down |
| MsG0180000993.01 | -2.004691241 | 6.92E-05 | yes | down |
| MsG0180001019.01 | -2.294215868 | 0.000132 | yes | down |
| MsG0180001068.01 | 1.245429094 | 0.001046 | yes | up |
| MsG0180001325.01 | 7.327009988 | 1.16E-06 | yes | up |
| MsG0180001480.01 | -2.693615081 | 8.30E-06 | yes | down |
| MsG0180001484.01 | 1.772078698 | 1.52E-05 | yes | up |
| MsG0180001598.01 | 1.466502676 | 0.000532 | yes | up |
| MsG0180001609.01 | 7.174548545 | 0.001229 | yes | up |
| MsG0180001786.01 | 10.75436605 | 3.29E-06 | yes | up |
| MsG0180001799.01 | -1.737410832 | 0.000171 | yes | down |
| MsG0180001856.01 | -5.991006716 | 0.000471 | yes | down |
| MsG0180002030.01 | 5.102017164 | 0.00012 | yes | up |
| MsG0180002138.01 | 3.314490251 | 0.000175 | yes | up |
| MsG0180002226.01 | -6.369390445 | 2.98E-05 | yes | down |
| MsG0180002272.01 | -1.427963801 | 1.46E-05 | yes | down |
| MsG0180002325.01 | -3.926786777 | 0.002032 | yes | down |
| MsG0180002362.01 | 2.546486134 | 0.000236 | yes | up |
| MsG0180002438.01 | -7.080317693 | 4.28E-06 | yes | down |
| MsG0180002458.01 | -1.234985407 | 0.000334 | yes | down |
| MsG0180002490.01 | -1.365727521 | 0.001288 | yes | down |
| MsG0180002793.01 | 6.051182451 | 0.002253 | yes | up |
| MsG0180002830.01 | 3.351769402 | 1.29E-05 | yes | up |
| MsG0180002924.01 | 2.499315527 | 3.17E-07 | yes | up |
| MsG0180002931.01 | -1.586983278 | 0.000956 | yes | down |
| MsG0180002936.01 | -2.121194452 | 3.38E-05 | yes | down |
| MsG0180003243.01 | -4.976113699 | 2.98E-07 | yes | down |
| MsG0180003377.01 | 2.148491841 | 0.000994 | yes | up |
| MsG0180003404.01 | -1.454415688 | 0.000262 | yes | down |
| MsG0180003484.01 | -2.384455894 | 0.000136 | yes | down |
| MsG0180003711.01 | 8.593778239 | 8.64E-10 | yes | up |
| MsG0180003753.01 | 3.546543129 | 0.001483 | yes | up |
| MsG0180003784.01 | -7.616280808 | 1.28E-05 | yes | down |
| MsG0180003808.01 | 3.986039887 | 5.16E-07 | yes | up |
| MsG0180003854.01 | -1.096531069 | 0.00109 | yes | down |
| MsG0180003906.01 | -1.918678825 | 0.000197 | yes | down |
| MsG0180003920.01 | -1.571370935 | 0.000464 | yes | down |
| MsG0180003922.01 | -2.738025687 | 0.000103 | yes | down |
| MsG0180003932.01 | 6.590898726 | 2.38E-07 | yes | up |
| MsG0180003992.01 | 2.145283542 | 0.001099 | yes | up |
| MsG0180003993.01 | -2.937628695 | 0.001048 | yes | down |
| MsG0180004027.01 | -2.9059435 | 0.000774 | yes | down |
| MsG0180004037.01 | -4.830468834 | 1.10E-06 | yes | down |
| MsG0180004091.01 | -4.647657421 | 4.20E-11 | yes | down |
| MsG0180004094.01 | -5.24192355 | 8.89E-07 | yes | down |
| MsG0180004099.01 | -2.767418685 | 2.65E-06 | yes | down |
| MsG0180004102.01 | -3.480148535 | 9.64E-09 | yes | down |
| MsG0180004103.01 | 1.103514489 | 0.000813 | yes | up |
| MsG0180004104.01 | 2.212578412 | 0.00045 | yes | up |
| MsG0180004110.01 | 2.708752496 | 1.07E-07 | yes | up |
| MsG0180004126.01 | -1.885971216 | 0.000959 | yes | down |
| MsG0180004130.01 | -1.288768049 | 8.20E-05 | yes | down |
| MsG0180004293.01 | -1.948368456 | 7.27E-05 | yes | down |
| MsG0180004385.01 | 1.291510462 | 7.96E-06 | yes | up |
| MsG0180004622.01 | 2.002204843 | 0.000494 | yes | up |
| MsG0180004623.01 | 2.125050077 | 0.000405 | yes | up |
| MsG0180004692.01 | 3.699095063 | 4.44E-10 | yes | up |
| MsG0180004741.01 | -1.697020931 | 3.30E-05 | yes | down |
| MsG0180004801.01 | 2.045404781 | 0.001349 | yes | up |
| MsG0180004817.01 | -1.972445263 | 0.000186 | yes | down |
| MsG0180004830.01 | -4.599497584 | 4.23E-07 | yes | down |
| MsG0180004866.01 | -1.941047253 | 0.001936 | yes | down |
| MsG0180004915.01 | -1.028857038 | 0.001077 | yes | down |
| MsG0180004917.01 | 2.093838774 | 0.000169 | yes | up |
| MsG0180004921.01 | 1.04752252 | 0.000139 | yes | up |
| MsG0180004977.01 | 4.463970762 | 4.65E-09 | yes | up |
| MsG0180004978.01 | 3.35859433 | 3.68E-06 | yes | up |
| MsG0180005005.01 | 1.185555315 | 0.001627 | yes | up |
| MsG0180005121.01 | 2.426874683 | 4.64E-06 | yes | up |
| MsG0180005142.01 | 3.808055061 | 0.000608 | yes | up |
| MsG0180005151.01 | -1.859935463 | 0.001611 | yes | down |
| MsG0180005253.01 | -1.200489883 | 0.000358 | yes | down |
| MsG0180005298.01 | -1.337447233 | 0.000144 | yes | down |
| MsG0180005308.01 | 2.909653963 | 8.06E-11 | yes | up |
| MsG0180005382.01 | -2.136183609 | 6.41E-13 | yes | down |
| MsG0180005418.01 | 1.323461401 | 0.000965 | yes | up |
| MsG0180005642.01 | -1.664560862 | 0.000295 | yes | down |
| MsG0180005673.01 | 4.467398735 | 0.000942 | yes | up |
| MsG0180005687.01 | 3.544583608 | 7.35E-05 | yes | up |
| MsG0180005718.01 | 1.393750519 | 4.61E-06 | yes | up |
| MsG0180005783.01 | 1.256175621 | 0.000186 | yes | up |
| MsG0180005819.01 | -1.116503972 | 0.000101 | yes | down |
| MsG0180006081.01 | 1.945568581 | 2.70E-05 | yes | up |
| MsG0180006211.01 | 6.12354463 | 7.76E-06 | yes | up |
| MsG0280006415.01 | -2.471127498 | 0.000724 | yes | down |
| MsG0280006468.01 | 1.682192204 | 0.000741 | yes | up |
| MsG0280006479.01 | 1.366708251 | 1.61E-05 | yes | up |
| MsG0280006533.01 | 1.15697234 | 0.000951 | yes | up |
| MsG0280006550.01 | 5.417347913 | 0.00162 | yes | up |
| MsG0280006725.01 | 1.063807016 | 1.90E-05 | yes | up |
| MsG0280006740.01 | 3.778916234 | 0.000883 | yes | up |
| MsG0280006855.01 | -3.52846045 | 3.94E-07 | yes | down |
| MsG0280006918.01 | 2.920628655 | 0.002034 | yes | up |
| MsG0280006919.01 | 4.416260336 | 4.66E-05 | yes | up |
| MsG0280006932.01 | 2.170808223 | 0.001545 | yes | up |
| MsG0280006942.01 | -2.779656719 | 7.98E-12 | yes | down |
| MsG0280006973.01 | 1.738563055 | 4.01E-05 | yes | up |
| MsG0280007124.01 | 1.32815756 | 0.001478 | yes | up |
| MsG0280007176.01 | 4.388292652 | 2.69E-05 | yes | up |
| MsG0280007208.01 | 1.83133021 | 9.85E-05 | yes | up |
| MsG0280007212.01 | -2.352861119 | 6.41E-05 | yes | down |
| MsG0280007337.01 | -3.257765603 | 0.000793 | yes | down |
| MsG0280007354.01 | 3.25713806 | 3.29E-10 | yes | up |
| MsG0280007427.01 | -1.405703554 | 0.000655 | yes | down |
| MsG0280007478.01 | 6.037174122 | 0.000294 | yes | up |
| MsG0280007481.01 | -1.544650189 | 9.92E-05 | yes | down |
| MsG0280007677.01 | 2.235230648 | 2.22E-05 | yes | up |
| MsG0280007684.01 | -1.680055182 | 0.000142 | yes | down |
| MsG0280007699.01 | -2.001048441 | 2.24E-06 | yes | down |
| MsG0280007708.01 | -2.876029211 | 0.001876 | yes | down |
| MsG0280007781.01 | -1.491576836 | 0.002083 | yes | down |
| MsG0280007827.01 | 3.140490533 | 4.33E-08 | yes | up |
| MsG0280007842.01 | -1.212178413 | 0.002041 | yes | down |
| MsG0280007870.01 | 1.130865807 | 0.001157 | yes | up |
| MsG0280007878.01 | -3.014657487 | 0.001554 | yes | down |
| MsG0280007883.01 | 2.111070419 | 0.000216 | yes | up |
| MsG0280007906.01 | 3.396022863 | 6.83E-06 | yes | up |
| MsG0280007911.01 | 3.10637296 | 4.20E-06 | yes | up |
| MsG0280007923.01 | -1.612577965 | 3.65E-07 | yes | down |
| MsG0280007990.01 | 4.256312566 | 0.000284 | yes | up |
| MsG0280008072.01 | 2.072220202 | 0.000678 | yes | up |
| MsG0280008107.01 | -2.142778028 | 0.001337 | yes | down |
| MsG0280008228.01 | 2.053865053 | 0.001739 | yes | up |
| MsG0280008257.01 | -1.604908851 | 0.000862 | yes | down |
| MsG0280008258.01 | 2.170745538 | 1.48E-05 | yes | up |
| MsG0280008334.01 | -2.499749481 | 0.001113 | yes | down |
| MsG0280008349.01 | -3.943043748 | 1.42E-07 | yes | down |
| MsG0280008350.01 | -5.264232937 | 0.000498 | yes | down |
| MsG0280008352.01 | -5.501405129 | 2.45E-08 | yes | down |
| MsG0280008357.01 | -5.355376041 | 2.08E-06 | yes | down |
| MsG0280008400.01 | 1.164610247 | 0.001176 | yes | up |
| MsG0280008597.01 | 2.651618302 | 0.000699 | yes | up |
| MsG0280008601.01 | 3.210781342 | 0.000713 | yes | up |
| MsG0280008643.01 | -1.160206564 | 0.001964 | yes | down |
| MsG0280008674.01 | 2.326306201 | 7.70E-05 | yes | up |
| MsG0280008676.01 | 4.58260969 | 0.000198 | yes | up |
| MsG0280008678.01 | 2.037979152 | 0.000163 | yes | up |
| MsG0280008720.01 | 7.378973944 | 0.001036 | yes | up |
| MsG0280008756.01 | 1.236036436 | 0.001813 | yes | up |
| MsG0280008764.01 | -2.999467822 | 4.56E-10 | yes | down |
| MsG0280008879.01 | 1.206673671 | 0.000578 | yes | up |
| MsG0280008988.01 | 2.665423218 | 0.000414 | yes | up |
| MsG0280009054.01 | 3.650106517 | 0.000308 | yes | up |
| MsG0280009072.01 | 2.519408869 | 0.000373 | yes | up |
| MsG0280009139.01 | -1.510506437 | 1.98E-05 | yes | down |
| MsG0280009321.01 | -3.315562843 | 0.001816 | yes | down |
| MsG0280009541.01 | -1.050907194 | 2.79E-05 | yes | down |
| MsG0280009582.01 | -7.013111124 | 2.51E-05 | yes | down |
| MsG0280009662.01 | 1.849114623 | 0.000657 | yes | up |
| MsG0280009717.01 | 2.713429662 | 4.77E-05 | yes | up |
| MsG0280009812.01 | -1.449144614 | 3.40E-06 | yes | down |
| MsG0280009894.01 | 2.566098501 | 0.000752 | yes | up |
| MsG0280009919.01 | 1.920830845 | 0.00051 | yes | up |
| MsG0280009982.01 | -4.151395367 | 0.00032 | yes | down |
| MsG0280009992.01 | 2.148219577 | 0.001036 | yes | up |
| MsG0280010244.01 | 1.983114287 | 2.05E-11 | yes | up |
| MsG0280010248.01 | -1.843180757 | 8.72E-07 | yes | down |
| MsG0280010298.01 | 2.826805218 | 0.001836 | yes | up |
| MsG0280010301.01 | -6.25122076 | 1.68E-06 | yes | down |
| MsG0280010303.01 | -6.161667934 | 7.55E-25 | yes | down |
| MsG0280010311.01 | 6.98312868 | 0.000716 | yes | up |
| MsG0280010378.01 | -1.789724889 | 0.000453 | yes | down |
| MsG0280010428.01 | 4.475250095 | 9.20E-05 | yes | up |
| MsG0280010489.01 | -4.441110126 | 8.68E-05 | yes | down |
| MsG0280010551.01 | 1.953476456 | 0.002087 | yes | up |
| MsG0280010593.01 | -6.307776542 | 0.000335 | yes | down |
| MsG0280010598.01 | 1.685934989 | 9.84E-05 | yes | up |
| MsG0280010607.01 | -2.630230962 | 0.000288 | yes | down |
| MsG0280010648.01 | 3.525884537 | 0.002133 | yes | up |
| MsG0280010661.01 | 1.931752802 | 0.000964 | yes | up |
| MsG0280010702.01 | 2.645483632 | 3.98E-10 | yes | up |
| MsG0280010707.01 | 1.808605324 | 4.28E-05 | yes | up |
| MsG0280010728.01 | 2.177381743 | 3.05E-05 | yes | up |
| MsG0280010769.01 | 2.179403828 | 5.29E-05 | yes | up |
| MsG0280010802.01 | 1.600894367 | 0.001598 | yes | up |
| MsG0280010856.01 | -1.936874541 | 0.000264 | yes | down |
| MsG0280010864.01 | -6.766653009 | 0.000843 | yes | down |
| MsG0280010899.01 | -1.695008276 | 1.94E-07 | yes | down |
| MsG0280010901.01 | 1.20964075 | 0.001707 | yes | up |
| MsG0280010916.01 | 1.543066127 | 4.07E-06 | yes | up |
| MsG0280011027.01 | -1.219365861 | 0.001224 | yes | down |
| MsG0280011054.01 | 1.161328408 | 9.32E-05 | yes | up |
| MsG0280011162.01 | 2.105920075 | 0.001598 | yes | up |
| MsG0280011288.01 | -2.170143338 | 0.00034 | yes | down |
| MsG0280011470.01 | -2.34588294 | 0.000321 | yes | down |
| MsG0380011483.01 | 7.774656956 | 2.13E-07 | yes | up |
| MsG0380011649.01 | 4.793679627 | 5.68E-05 | yes | up |
| MsG0380011666.01 | -1.415281825 | 2.10E-05 | yes | down |
| MsG0380011745.01 | -3.829374481 | 1.07E-09 | yes | down |
| MsG0380011895.01 | 1.409588456 | 0.001538 | yes | up |
| MsG0380011921.01 | 2.612984954 | 0.000121 | yes | up |
| MsG0380011962.01 | 3.491954674 | 0.000566 | yes | up |
| MsG0380012035.01 | 1.164912771 | 0.001451 | yes | up |
| MsG0380012148.01 | 1.497104861 | 4.65E-05 | yes | up |
| MsG0380012194.01 | 2.441591872 | 0.001769 | yes | up |
| MsG0380012197.01 | 2.816877134 | 2.17E-06 | yes | up |
| MsG0380012211.01 | 5.36503543 | 2.45E-06 | yes | up |
| MsG0380012264.01 | -2.24159946 | 0.001758 | yes | down |
| MsG0380012272.01 | 2.435242294 | 8.42E-06 | yes | up |
| MsG0380012411.01 | 5.175389107 | 4.85E-08 | yes | up |
| MsG0380012513.01 | -2.02289145 | 0.001597 | yes | down |
| MsG0380012553.01 | 1.507881613 | 0.002251 | yes | up |
| MsG0380012558.01 | 4.924102415 | 0.000504 | yes | up |
| MsG0380012566.01 | -1.380637683 | 0.000179 | yes | down |
| MsG0380012788.01 | -3.669898848 | 0.000177 | yes | down |
| MsG0380012807.01 | -3.979632763 | 0.000152 | yes | down |
| MsG0380012869.01 | 1.446248121 | 0.001328 | yes | up |
| MsG0380013478.01 | -2.153172589 | 2.33E-07 | yes | down |
| MsG0380013547.01 | -1.068580245 | 0.001457 | yes | down |
| MsG0380013761.01 | 7.950339032 | 2.35E-05 | yes | up |
| MsG0380013785.01 | 7.162249036 | 1.50E-05 | yes | up |
| MsG0380013788.01 | -6.503428765 | 0.000426 | yes | down |
| MsG0380013796.01 | 2.658765883 | 0.000705 | yes | up |
| MsG0380013802.01 | 3.396153146 | 5.96E-05 | yes | up |
| MsG0380013856.01 | 3.406555941 | 0.00103 | yes | up |
| MsG0380013949.01 | -4.066792383 | 1.59E-12 | yes | down |
| MsG0380013971.01 | -3.54474982 | 3.72E-10 | yes | down |
| MsG0380013973.01 | -9.333822366 | 8.94E-13 | yes | down |
| MsG0380013974.01 | -4.463358927 | 2.64E-35 | yes | down |
| MsG0380014047.01 | -12.6809864 | 1.48E-24 | yes | down |
| MsG0380014133.01 | 3.773047911 | 0.00013 | yes | up |
| MsG0380014233.01 | -4.658922043 | 5.58E-06 | yes | down |
| MsG0380014276.01 | -1.238550556 | 1.10E-05 | yes | down |
| MsG0380014410.01 | 3.135005196 | 1.52E-06 | yes | up |
| MsG0380014422.01 | -6.676554461 | 4.50E-05 | yes | down |
| MsG0380014438.01 | -5.454105608 | 0.000568 | yes | down |
| MsG0380014582.01 | 1.26881527 | 0.001897 | yes | up |
| MsG0380014748.01 | -6.858738661 | 6.92E-15 | yes | down |
| MsG0380014847.01 | -2.358398518 | 1.90E-05 | yes | down |
| MsG0380014933.01 | -3.250817845 | 0.001528 | yes | down |
| MsG0380015095.01 | 3.926326865 | 3.67E-06 | yes | up |
| MsG0380015135.01 | -6.421236034 | 0.000197 | yes | down |
| MsG0380015148.01 | 1.321100332 | 0.002155 | yes | up |
| MsG0380015152.01 | 6.571028017 | 8.46E-05 | yes | up |
| MsG0380015174.01 | 1.631720288 | 0.000267 | yes | up |
| MsG0380015187.01 | 6.550586196 | 0.001172 | yes | up |
| MsG0380015189.01 | -5.77364237 | 0.00055 | yes | down |
| MsG0380015233.01 | 4.385472025 | 0.000736 | yes | up |
| MsG0380015267.01 | -6.661416296 | 2.07E-05 | yes | down |
| MsG0380015268.01 | -3.108066051 | 4.54E-05 | yes | down |
| MsG0380015326.01 | 3.192168135 | 0.001503 | yes | up |
| MsG0380015328.01 | 3.167370661 | 0.001136 | yes | up |
| MsG0380015414.01 | 2.827403959 | 2.55E-12 | yes | up |
| MsG0380015497.01 | 1.653660533 | 9.01E-06 | yes | up |
| MsG0380015498.01 | -8.005258474 | 5.09E-09 | yes | down |
| MsG0380015499.01 | 2.249105225 | 5.66E-06 | yes | up |
| MsG0380015519.01 | 4.96429706 | 0.000788 | yes | up |
| MsG0380015544.01 | -1.776139094 | 0.000429 | yes | down |
| MsG0380015691.01 | -2.548050505 | 0.001269 | yes | down |
| MsG0380015722.01 | -6.801827008 | 6.35E-05 | yes | down |
| MsG0380015739.01 | 1.382262073 | 3.71E-05 | yes | up |
| MsG0380015801.01 | 1.012573565 | 4.14E-05 | yes | up |
| MsG0380015819.01 | 1.706840723 | 3.25E-06 | yes | up |
| MsG0380015825.01 | -2.621380392 | 2.40E-09 | yes | down |
| MsG0380015834.01 | -2.297088128 | 0.000793 | yes | down |
| MsG0380015858.01 | -1.922263618 | 0.001693 | yes | down |
| MsG0380015862.01 | -1.845922388 | 3.00E-06 | yes | down |
| MsG0380015944.01 | -5.611577795 | 0.000204 | yes | down |
| MsG0380016028.01 | 4.48722194 | 9.71E-15 | yes | up |
| MsG0380016080.01 | -2.606723292 | 2.88E-05 | yes | down |
| MsG0380016110.01 | -3.181527363 | 0.000513 | yes | down |
| MsG0380016334.01 | -2.195027578 | 0.000998 | yes | down |
| MsG0380016341.01 | 6.308860066 | 0.00022 | yes | up |
| MsG0380016368.01 | -1.385310377 | 7.57E-05 | yes | down |
| MsG0380016458.01 | -3.95848191 | 5.30E-07 | yes | down |
| MsG0380016853.01 | 1.071978601 | 0.000256 | yes | up |
| MsG0380016941.01 | 5.001664572 | 9.99E-05 | yes | up |
| MsG0380016948.01 | 4.493910974 | 7.40E-06 | yes | up |
| MsG0380016962.01 | -1.861394919 | 0.001024 | yes | down |
| MsG0380016977.01 | -1.247076153 | 0.00178 | yes | down |
| MsG0380017008.01 | 7.161583566 | 7.22E-05 | yes | up |
| MsG0380017109.01 | -7.027991311 | 2.21E-05 | yes | down |
| MsG0380017155.01 | -1.075302649 | 0.000175 | yes | down |
| MsG0380017189.01 | 4.754155131 | 5.53E-09 | yes | up |
| MsG0380017237.01 | -3.208204917 | 0.000214 | yes | down |
| MsG0380017247.01 | 2.403218265 | 0.00098 | yes | up |
| MsG0380017382.01 | -1.447968262 | 2.57E-06 | yes | down |
| MsG0380017394.01 | -4.194453136 | 0.000277 | yes | down |
| MsG0380017404.01 | -2.237129455 | 2.22E-07 | yes | down |
| MsG0380017441.01 | -1.961563894 | 0.001602 | yes | down |
| MsG0380017502.01 | 2.652300685 | 0.000288 | yes | up |
| MsG0380017598.01 | 4.64557516 | 1.20E-07 | yes | up |
| MsG0380017606.01 | -1.798943811 | 6.80E-11 | yes | down |
| MsG0380017607.01 | 2.748989351 | 0.001598 | yes | up |
| MsG0380017614.01 | 1.954407019 | 2.87E-05 | yes | up |
| MsG0380017683.01 | 1.972170349 | 1.66E-05 | yes | up |
| MsG0380017684.01 | 2.139378497 | 1.91E-06 | yes | up |
| MsG0380017699.01 | 2.30345633 | 3.70E-05 | yes | up |
| MsG0380017707.01 | 1.797777657 | 7.89E-08 | yes | up |
| MsG0380017788.01 | -1.463792825 | 8.16E-06 | yes | down |
| MsG0380017791.01 | 1.503693941 | 1.65E-05 | yes | up |
| MsG0380017793.01 | 1.39614861 | 8.11E-07 | yes | up |
| MsG0380017883.01 | 1.017766088 | 0.002208 | yes | up |
| MsG0380017913.01 | -1.534573395 | 0.000323 | yes | down |
| MsG0380017926.01 | 1.154934923 | 1.87E-05 | yes | up |
| MsG0380017993.01 | 4.028651067 | 0.000892 | yes | up |
| MsG0380018025.01 | 3.972698835 | 1.57E-06 | yes | up |
| MsG0380018047.01 | -1.532591839 | 0.000707 | yes | down |
| MsG0380018054.01 | 1.200372608 | 0.001608 | yes | up |
| MsG0480018099.01 | -9.097953854 | 8.93E-12 | yes | down |
| MsG0480018146.01 | -6.031754937 | 0.001328 | yes | down |
| MsG0480018187.01 | 1.858836692 | 0.000143 | yes | up |
| MsG0480018219.01 | 1.8098697 | 0.000305 | yes | up |
| MsG0480018276.01 | 4.936218068 | 0.001259 | yes | up |
| MsG0480018324.01 | -1.203642244 | 0.002113 | yes | down |
| MsG0480018351.01 | -2.552328572 | 0.001952 | yes | down |
| MsG0480018352.01 | -2.098159321 | 5.34E-05 | yes | down |
| MsG0480018484.01 | -3.891077204 | 0.00023 | yes | down |
| MsG0480018496.01 | 1.448361432 | 0.000353 | yes | up |
| MsG0480018742.01 | -4.189346141 | 1.33E-05 | yes | down |
| MsG0480018745.01 | -3.211571463 | 3.39E-05 | yes | down |
| MsG0480018752.01 | -2.450060233 | 2.00E-06 | yes | down |
| MsG0480018982.01 | 1.61254789 | 8.08E-06 | yes | up |
| MsG0480018991.01 | -3.474010991 | 0.001438 | yes | down |
| MsG0480019005.01 | -2.114586247 | 0.000788 | yes | down |
| MsG0480019218.01 | 1.565808594 | 0.000388 | yes | up |
| MsG0480019359.01 | -2.5300443 | 0.001706 | yes | down |
| MsG0480019426.01 | -1.740974479 | 0.000488 | yes | down |
| MsG0480019502.01 | 1.10584453 | 2.35E-05 | yes | up |
| MsG0480019538.01 | -6.036763858 | 1.30E-13 | yes | down |
| MsG0480019829.01 | 1.971354932 | 0.001582 | yes | up |
| MsG0480020083.01 | -2.385232755 | 0.001609 | yes | down |
| MsG0480020134.01 | -1.175540583 | 0.001343 | yes | down |
| MsG0480020151.01 | 1.612788403 | 2.55E-05 | yes | up |
| MsG0480020220.01 | 1.286614148 | 2.85E-05 | yes | up |
| MsG0480020259.01 | -4.401402261 | 7.53E-37 | yes | down |
| MsG0480020344.01 | 7.156867508 | 3.45E-05 | yes | up |
| MsG0480020366.01 | -2.510689482 | 8.49E-07 | yes | down |
| MsG0480020392.01 | 6.547247767 | 0.001398 | yes | up |
| MsG0480020418.01 | -1.958919983 | 0.001434 | yes | down |
| MsG0480020492.01 | -3.505808648 | 0.001532 | yes | down |
| MsG0480020534.01 | -2.210026458 | 7.64E-06 | yes | down |
| MsG0480020547.01 | -1.523249669 | 1.70E-07 | yes | down |
| MsG0480020596.01 | 7.177634027 | 1.52E-05 | yes | up |
| MsG0480020818.01 | -4.277108939 | 1.91E-07 | yes | down |
| MsG0480020827.01 | 1.313154415 | 0.000283 | yes | up |
| MsG0480020880.01 | -7.911128047 | 3.24E-05 | yes | down |
| MsG0480020896.01 | 2.674543758 | 0.001891 | yes | up |
| MsG0480020913.01 | 1.03129885 | 0.001432 | yes | up |
| MsG0480020939.01 | -1.388987106 | 4.86E-08 | yes | down |
| MsG0480020941.01 | -1.304871211 | 3.75E-05 | yes | down |
| MsG0480020959.01 | -1.408924752 | 1.20E-05 | yes | down |
| MsG0480021198.01 | -4.494066357 | 0.001297 | yes | down |
| MsG0480021199.01 | -4.17852671 | 3.08E-05 | yes | down |
| MsG0480021200.01 | -2.929835929 | 0.000236 | yes | down |
| MsG0480021267.01 | -1.673796996 | 0.002221 | yes | down |
| MsG0480021420.01 | 1.558249673 | 0.001021 | yes | up |
| MsG0480021489.01 | 1.341492448 | 0.001044 | yes | up |
| MsG0480021529.01 | -6.041729737 | 3.99E-07 | yes | down |
| MsG0480021587.01 | -4.824929146 | 9.67E-05 | yes | down |
| MsG0480021599.01 | 6.647030744 | 1.10E-05 | yes | up |
| MsG0480021600.01 | 2.973468736 | 0.000196 | yes | up |
| MsG0480021655.01 | -1.299367007 | 0.001017 | yes | down |
| MsG0480021692.01 | 1.805363925 | 0.000817 | yes | up |
| MsG0480021761.01 | -1.45885474 | 0.000458 | yes | down |
| MsG0480021891.01 | 1.314353827 | 0.001154 | yes | up |
| MsG0480021913.01 | -1.155648737 | 0.001065 | yes | down |
| MsG0480021976.01 | 1.377674415 | 0.000516 | yes | up |
| MsG0480022032.01 | 1.235825875 | 2.37E-05 | yes | up |
| MsG0480022039.01 | -3.05273881 | 0.001444 | yes | down |
| MsG0480022053.01 | 2.753988669 | 3.61E-15 | yes | up |
| MsG0480022099.01 | 1.355097778 | 0.000176 | yes | up |
| MsG0480022111.01 | 6.089631776 | 0.000117 | yes | up |
| MsG0480022177.01 | 1.067411708 | 0.001637 | yes | up |
| MsG0480022178.01 | -1.954989742 | 5.56E-06 | yes | down |
| MsG0480022209.01 | 6.765474193 | 0.000126 | yes | up |
| MsG0480022322.01 | -1.706628569 | 0.002031 | yes | down |
| MsG0480022342.01 | -3.083852212 | 0.001586 | yes | down |
| MsG0480022345.01 | -3.369441905 | 6.06E-06 | yes | down |
| MsG0480022346.01 | -4.992823166 | 0.000172 | yes | down |
| MsG0480022470.01 | 4.332555148 | 4.41E-05 | yes | up |
| MsG0480022501.01 | -1.379624843 | 0.000428 | yes | down |
| MsG0480022512.01 | 1.223133335 | 1.14E-05 | yes | up |
| MsG0480022515.01 | 2.781592922 | 2.59E-09 | yes | up |
| MsG0480022595.01 | 1.111815931 | 0.001685 | yes | up |
| MsG0480022599.01 | -1.022176372 | 0.00048 | yes | down |
| MsG0480022639.01 | -1.639357387 | 0.001351 | yes | down |
| MsG0480022642.01 | -1.502594599 | 0.001057 | yes | down |
| MsG0480022662.01 | -4.2163561 | 3.96E-15 | yes | down |
| MsG0480022664.01 | 1.113567004 | 0.001117 | yes | up |
| MsG0480022703.01 | -5.697989821 | 3.46E-13 | yes | down |
| MsG0480022724.01 | 2.19475414 | 0.001822 | yes | up |
| MsG0480022746.01 | 2.260227701 | 0.000735 | yes | up |
| MsG0480022813.01 | 1.980009925 | 0.001803 | yes | up |
| MsG0480022848.01 | -1.712372598 | 0.000396 | yes | down |
| MsG0480022895.01 | 1.068132679 | 0.00029 | yes | up |
| MsG0480022900.01 | 3.010878332 | 0.00186 | yes | up |
| MsG0480022910.01 | -1.740720241 | 4.60E-05 | yes | down |
| MsG0480023021.01 | 7.139988942 | 0.001505 | yes | up |
| MsG0480023026.01 | 1.394198985 | 0.000212 | yes | up |
| MsG0480023102.01 | 2.072822288 | 0.000219 | yes | up |
| MsG0480023114.01 | -2.476248877 | 0.000762 | yes | down |
| MsG0480023229.01 | 6.971294192 | 0.000685 | yes | up |
| MsG0480023238.01 | 1.690909829 | 2.97E-05 | yes | up |
| MsG0480023248.01 | 1.509593069 | 0.002196 | yes | up |
| MsG0480023251.01 | 1.458869779 | 0.000567 | yes | up |
| MsG0480023368.01 | 4.315923342 | 0.000364 | yes | up |
| MsG0480023401.01 | 1.369939852 | 0.000352 | yes | up |
| MsG0480023416.01 | 1.167553556 | 3.94E-05 | yes | up |
| MsG0480023549.01 | 2.03095177 | 1.81E-05 | yes | up |
| MsG0480023550.01 | -1.244108227 | 0.001924 | yes | down |
| MsG0480023551.01 | -7.156505193 | 2.63E-07 | yes | down |
| MsG0480023604.01 | 2.914252637 | 2.00E-13 | yes | up |
| MsG0480023606.01 | 2.412135535 | 0.000829 | yes | up |
| MsG0480023627.01 | -1.096214631 | 1.24E-05 | yes | down |
| MsG0480023630.01 | 4.38673299 | 0.000222 | yes | up |
| MsG0480023682.01 | -3.697508953 | 0.000699 | yes | down |
| MsG0480023689.01 | -2.132256617 | 9.45E-07 | yes | down |
| MsG0480023742.01 | -2.919399706 | 0.00029 | yes | down |
| MsG0480023761.01 | 2.332715064 | 5.46E-05 | yes | up |
| MsG0480023819.01 | 1.827383646 | 0.001434 | yes | up |
| MsG0480023911.01 | -2.850835365 | 1.84E-05 | yes | down |
| MsG0480023916.01 | 4.759452676 | 0.00126 | yes | up |
| MsG0480023944.01 | -1.059202019 | 4.36E-05 | yes | down |
| MsG0480023969.01 | -1.100676932 | 1.18E-05 | yes | down |
| MsG0580024151.01 | -4.137081809 | 2.96E-05 | yes | down |
| MsG0580024168.01 | -4.93527228 | 7.53E-05 | yes | down |
| MsG0580024193.01 | -1.620295734 | 3.84E-05 | yes | down |
| MsG0580024217.01 | 3.591396045 | 0.001039 | yes | up |
| MsG0580024328.01 | 1.724280293 | 0.000286 | yes | up |
| MsG0580024333.01 | 1.670952188 | 0.000121 | yes | up |
| MsG0580024401.01 | -4.528034553 | 3.27E-05 | yes | down |
| MsG0580024405.01 | 2.453304106 | 1.02E-07 | yes | up |
| MsG0580024467.01 | 1.59698904 | 0.001401 | yes | up |
| MsG0580024487.01 | 1.33422465 | 0.001587 | yes | up |
| MsG0580024522.01 | 5.064439253 | 0.000763 | yes | up |
| MsG0580024551.01 | 1.658981895 | 0.000667 | yes | up |
| MsG0580024656.01 | -2.779656719 | 7.98E-12 | yes | down |
| MsG0580024782.01 | -2.416040008 | 0.000698 | yes | down |
| MsG0580024842.01 | -1.866217956 | 2.04E-05 | yes | down |
| MsG0580024855.01 | -1.676664747 | 0.001898 | yes | down |
| MsG0580024974.01 | -1.23720293 | 2.99E-05 | yes | down |
| MsG0580024996.01 | 1.072725219 | 0.00024 | yes | up |
| MsG0580025099.01 | 1.71360162 | 0.000213 | yes | up |
| MsG0580025261.01 | -2.348140182 | 0.001098 | yes | down |
| MsG0580025316.01 | -4.281210134 | 0.000707 | yes | down |
| MsG0580025352.01 | -1.255500371 | 0.00043 | yes | down |
| MsG0580025409.01 | -1.436325008 | 7.03E-05 | yes | down |
| MsG0580025468.01 | -1.166632559 | 0.000714 | yes | down |
| MsG0580025514.01 | 5.265511813 | 0.000338 | yes | up |
| MsG0580025567.01 | -7.435776268 | 0.000732 | yes | down |
| MsG0580025584.01 | 1.488554895 | 0.00085 | yes | up |
| MsG0580025586.01 | 1.313094403 | 0.000457 | yes | up |
| MsG0580025612.01 | -1.830352485 | 7.38E-05 | yes | down |
| MsG0580025659.01 | 2.865631276 | 0.001076 | yes | up |
| MsG0580025664.01 | -1.421250361 | 0.000122 | yes | down |
| MsG0580025670.01 | -2.675397729 | 2.40E-07 | yes | down |
| MsG0580025674.01 | -2.030210836 | 2.70E-05 | yes | down |
| MsG0580025683.01 | 2.926670891 | 3.84E-06 | yes | up |
| MsG0580025808.01 | 1.026520666 | 4.93E-05 | yes | up |
| MsG0580025826.01 | 2.139195881 | 1.46E-08 | yes | up |
| MsG0580025833.01 | -1.465072886 | 0.000275 | yes | down |
| MsG0580025880.01 | 3.56649517 | 0.000419 | yes | up |
| MsG0580025906.01 | 1.429742705 | 6.65E-05 | yes | up |
| MsG0580026003.01 | 1.323045634 | 0.000714 | yes | up |
| MsG0580026026.01 | -1.450037869 | 0.001048 | yes | down |
| MsG0580026176.01 | -4.628722976 | 0.000194 | yes | down |
| MsG0580026206.01 | -4.730218321 | 2.70E-07 | yes | down |
| MsG0580026594.01 | 4.39725038 | 4.17E-06 | yes | up |
| MsG0580026702.01 | -6.101587414 | 1.93E-06 | yes | down |
| MsG0580026705.01 | -2.861058097 | 3.58E-05 | yes | down |
| MsG0580026737.01 | -4.912732573 | 0.001496 | yes | down |
| MsG0580026876.01 | 6.88285412 | 0.000526 | yes | up |
| MsG0580027088.01 | -2.282615395 | 0.001979 | yes | down |
| MsG0580027113.01 | -2.576271282 | 2.21E-06 | yes | down |
| MsG0580027139.01 | -7.655601777 | 0.000105 | yes | down |
| MsG0580027215.01 | 7.143967824 | 1.80E-05 | yes | up |
| MsG0580027221.01 | -2.517766538 | 0.002165 | yes | down |
| MsG0580027261.01 | -1.336702747 | 0.000481 | yes | down |
| MsG0580027449.01 | 1.906776516 | 3.77E-06 | yes | up |
| MsG0580027470.01 | -2.395509918 | 2.27E-05 | yes | down |
| MsG0580027513.01 | 6.848424596 | 0.000307 | yes | up |
| MsG0580027523.01 | 2.021104147 | 7.29E-05 | yes | up |
| MsG0580027605.01 | 4.711674967 | 1.22E-05 | yes | up |
| MsG0580027615.01 | 3.988926456 | 7.88E-06 | yes | up |
| MsG0580027661.01 | -3.18063275 | 0.000248 | yes | down |
| MsG0580027678.01 | -1.478916996 | 0.000601 | yes | down |
| MsG0580027710.01 | -6.8496734 | 3.38E-06 | yes | down |
| MsG0580027749.01 | 6.228676322 | 0.000847 | yes | up |
| MsG0580027788.01 | -3.044826076 | 1.36E-06 | yes | down |
| MsG0580027803.01 | 4.021025115 | 0.00012 | yes | up |
| MsG0580027828.01 | -2.6440807 | 5.77E-10 | yes | down |
| MsG0580027832.01 | -1.831648756 | 0.001219 | yes | down |
| MsG0580027899.01 | 2.798440071 | 5.83E-06 | yes | up |
| MsG0580028049.01 | 7.594561969 | 2.52E-05 | yes | up |
| MsG0580028107.01 | -1.606027487 | 0.001694 | yes | down |
| MsG0580028196.01 | 7.218633802 | 0.001522 | yes | up |
| MsG0580028238.01 | -1.528835338 | 5.49E-05 | yes | down |
| MsG0580028316.01 | 1.567927906 | 1.32E-11 | yes | up |
| MsG0580028422.01 | 3.465632735 | 6.54E-10 | yes | up |
| MsG0580028455.01 | 1.778331519 | 0.001322 | yes | up |
| MsG0580028496.01 | 5.078366411 | 1.77E-10 | yes | up |
| MsG0580028593.01 | 2.180944033 | 4.36E-10 | yes | up |
| MsG0580028614.01 | -1.379565275 | 0.000257 | yes | down |
| MsG0580028702.01 | 2.724488788 | 0.000229 | yes | up |
| MsG0580028710.01 | 1.56371382 | 0.001542 | yes | up |
| MsG0580028956.01 | 6.286635307 | 1.82E-06 | yes | up |
| MsG0580028957.01 | -1.343364218 | 1.03E-05 | yes | down |
| MsG0580028958.01 | -1.88299076 | 0.000688 | yes | down |
| MsG0580029047.01 | -1.220577175 | 1.61E-05 | yes | down |
| MsG0580029282.01 | -2.730092506 | 7.13E-10 | yes | down |
| MsG0580029312.01 | 2.619084855 | 6.72E-07 | yes | up |
| MsG0580029315.01 | 2.479296064 | 2.34E-07 | yes | up |
| MsG0580029397.01 | -2.019072483 | 0.00017 | yes | down |
| MsG0580029429.01 | 1.489092145 | 7.14E-05 | yes | up |
| MsG0580029467.01 | -9.738469301 | 0.001296 | yes | down |
| MsG0580029491.01 | 1.974909204 | 0.000869 | yes | up |
| MsG0580029519.01 | -7.157120845 | 0.000266 | yes | down |
| MsG0580029583.01 | 2.242273808 | 0.000242 | yes | up |
| MsG0580029697.01 | 1.262128663 | 0.00226 | yes | up |
| MsG0580029737.01 | 1.100734364 | 0.000281 | yes | up |
| MsG0580029953.01 | -1.282357054 | 3.89E-06 | yes | down |
| MsG0580030133.01 | 2.222548753 | 0.000617 | yes | up |
| MsG0580030135.01 | 1.941599434 | 0.001613 | yes | up |
| MsG0580030137.01 | 3.627614055 | 1.20E-08 | yes | up |
| MsG0580030199.01 | -1.278874356 | 0.001123 | yes | down |
| MsG0680030342.01 | 2.481871178 | 0.001314 | yes | up |
| MsG0680030359.01 | 2.855059344 | 0.000126 | yes | up |
| MsG0680030429.01 | 7.085418812 | 0.00159 | yes | up |
| MsG0680030529.01 | -5.679862436 | 0.000275 | yes | down |
| MsG0680030556.01 | -1.992027043 | 2.49E-06 | yes | down |
| MsG0680030558.01 | 1.188831446 | 0.001277 | yes | up |
| MsG0680030559.01 | 2.853609296 | 0.001072 | yes | up |
| MsG0680030604.01 | 4.522144933 | 0.002095 | yes | up |
| MsG0680030677.01 | -4.589775327 | 2.65E-12 | yes | down |
| MsG0680030746.01 | -1.224320078 | 0.001646 | yes | down |
| MsG0680030839.01 | 3.196415186 | 1.53E-05 | yes | up |
| MsG0680030892.01 | -2.0882175 | 0.001458 | yes | down |
| MsG0680030916.01 | 1.927628214 | 0.00036 | yes | up |
| MsG0680030918.01 | -1.560893791 | 5.19E-05 | yes | down |
| MsG0680030949.01 | 3.981234469 | 2.14E-06 | yes | up |
| MsG0680030953.01 | 3.948893918 | 0.000552 | yes | up |
| MsG0680030961.01 | -2.07783877 | 0.001967 | yes | down |
| MsG0680030976.01 | 4.127881534 | 1.91E-06 | yes | up |
| MsG0680031055.01 | 2.582474072 | 0.000653 | yes | up |
| MsG0680031087.01 | 2.020890486 | 0.001212 | yes | up |
| MsG0680031105.01 | -2.681336466 | 0.00156 | yes | down |
| MsG0680031150.01 | -1.508250181 | 5.05E-05 | yes | down |
| MsG0680031206.01 | 3.085891486 | 1.30E-07 | yes | up |
| MsG0680031241.01 | -1.264057885 | 0.000957 | yes | down |
| MsG0680031254.01 | 2.937957767 | 0.000875 | yes | up |
| MsG0680031277.01 | 1.800388353 | 0.000109 | yes | up |
| MsG0680031278.01 | 2.009946005 | 0.002199 | yes | up |
| MsG0680031280.01 | 7.006054833 | 0.002112 | yes | up |
| MsG0680031284.01 | 7.856863059 | 4.39E-05 | yes | up |
| MsG0680031286.01 | 6.420399963 | 0.00053 | yes | up |
| MsG0680031294.01 | 9.488253625 | 1.73E-29 | yes | up |
| MsG0680031318.01 | 1.931844181 | 0.00029 | yes | up |
| MsG0680031334.01 | 2.528834229 | 6.72E-07 | yes | up |
| MsG0680031368.01 | -1.411560083 | 1.89E-05 | yes | down |
| MsG0680031403.01 | 1.474496807 | 0.000742 | yes | up |
| MsG0680031428.01 | 6.237648004 | 0.001652 | yes | up |
| MsG0680031545.01 | 1.486643638 | 0.000633 | yes | up |
| MsG0680031814.01 | 3.016118388 | 2.64E-06 | yes | up |
| MsG0680031952.01 | 10.53253835 | 5.36E-17 | yes | up |
| MsG0680031962.01 | 4.505261845 | 1.77E-24 | yes | up |
| MsG0680031966.01 | 1.318914848 | 0.000245 | yes | up |
| MsG0680031996.01 | 4.197099182 | 0.000117 | yes | up |
| MsG0680031997.01 | 4.234628675 | 0.000212 | yes | up |
| MsG0680032019.01 | 2.945774304 | 5.68E-05 | yes | up |
| MsG0680032095.01 | 3.658451467 | 0.00028 | yes | up |
| MsG0680032201.01 | -2.842922592 | 0.001877 | yes | down |
| MsG0680032238.01 | 5.620397109 | 0.000163 | yes | up |
| MsG0680032245.01 | 1.852762045 | 0.002175 | yes | up |
| MsG0680032380.01 | 1.456074825 | 0.0015 | yes | up |
| MsG0680032431.01 | 3.364644272 | 0.000779 | yes | up |
| MsG0680032536.01 | 6.313649115 | 1.34E-26 | yes | up |
| MsG0680032542.01 | 7.243087548 | 0.000224 | yes | up |
| MsG0680032546.01 | 4.560182517 | 0.000184 | yes | up |
| MsG0680032551.01 | 5.373620882 | 0.000118 | yes | up |
| MsG0680032617.01 | -3.096296944 | 1.34E-06 | yes | down |
| MsG0680032686.01 | -9.390249052 | 6.25E-09 | yes | down |
| MsG0680032778.01 | 3.139260211 | 0.000577 | yes | up |
| MsG0680032833.01 | 1.879739639 | 0.001753 | yes | up |
| MsG0680032925.01 | -1.682143592 | 1.75E-06 | yes | down |
| MsG0680033003.01 | 2.141675793 | 0.000776 | yes | up |
| MsG0680033015.01 | -1.930188588 | 0.00102 | yes | down |
| MsG0680033485.01 | 7.305133379 | 3.77E-08 | yes | up |
| MsG0680033821.01 | 3.69915523 | 9.42E-06 | yes | up |
| MsG0680033830.01 | 2.021003035 | 0.001711 | yes | up |
| MsG0680033932.01 | -1.167959591 | 0.001131 | yes | down |
| MsG0680034028.01 | 3.860515324 | 8.16E-07 | yes | up |
| MsG0680034032.01 | 1.767290987 | 0.000673 | yes | up |
| MsG0680034036.01 | 1.770715667 | 0.000214 | yes | up |
| MsG0680034040.01 | -1.304414458 | 0.00051 | yes | down |
| MsG0680034110.01 | 7.971952273 | 2.27E-07 | yes | up |
| MsG0680034225.01 | -2.242590788 | 0.000174 | yes | down |
| MsG0680034255.01 | -2.802350311 | 0.001787 | yes | down |
| MsG0680034317.01 | -3.499017184 | 1.93E-05 | yes | down |
| MsG0680034376.01 | 1.628080081 | 0.000172 | yes | up |
| MsG0680034435.01 | -10.70604128 | 4.84E-05 | yes | down |
| MsG0680034475.01 | -1.285976722 | 0.000129 | yes | down |
| MsG0680034491.01 | 4.736828961 | 0.001447 | yes | up |
| MsG0680034495.01 | 3.172319486 | 2.69E-05 | yes | up |
| MsG0680034499.01 | 2.623004469 | 9.78E-07 | yes | up |
| MsG0680034506.01 | 7.746192999 | 3.29E-20 | yes | up |
| MsG0680034507.01 | 12.62698836 | 7.60E-08 | yes | up |
| MsG0680034510.01 | 6.386522378 | 0.000557 | yes | up |
| MsG0680034512.01 | 6.689733582 | 0.00081 | yes | up |
| MsG0680034513.01 | 8.780592422 | 1.71E-07 | yes | up |
| MsG0680034515.01 | 10.75124022 | 4.93E-08 | yes | up |
| MsG0680034516.01 | 8.1089257 | 1.55E-05 | yes | up |
| MsG0680034576.01 | 5.93241531 | 8.24E-10 | yes | up |
| MsG0680034583.01 | -7.130385769 | 0.001464 | yes | down |
| MsG0680034723.01 | -1.325046724 | 0.000755 | yes | down |
| MsG0680034797.01 | 4.640599211 | 0.001696 | yes | up |
| MsG0680034814.01 | -4.512368269 | 1.95E-05 | yes | down |
| MsG0680034852.01 | 4.15285362 | 1.37E-05 | yes | up |
| MsG0680034868.01 | -6.092984563 | 5.51E-06 | yes | down |
| MsG0680034914.01 | 1.220637145 | 2.06E-05 | yes | up |
| MsG0680034980.01 | 2.840908245 | 2.13E-13 | yes | up |
| MsG0680034998.01 | -1.728336996 | 0.000227 | yes | down |
| MsG0680035017.01 | 2.041835705 | 0.00179 | yes | up |
| MsG0680035102.01 | -3.475843063 | 3.39E-06 | yes | down |
| MsG0680035118.01 | -2.087696594 | 1.36E-14 | yes | down |
| MsG0680035323.01 | 3.584049322 | 0.000271 | yes | up |
| MsG0680035353.01 | -4.516772703 | 0.001617 | yes | down |
| MsG0680035493.01 | 4.689194846 | 3.62E-07 | yes | up |
| MsG0680035494.01 | 3.089964661 | 1.14E-07 | yes | up |
| MsG0680035514.01 | 1.834817475 | 0.000138 | yes | up |
| MsG0680035560.01 | 1.884214767 | 0.001472 | yes | up |
| MsG0680035575.01 | 1.493890398 | 1.42E-08 | yes | up |
| MsG0680035582.01 | 1.376571847 | 4.17E-06 | yes | up |
| MsG0680035589.01 | -2.678284246 | 0.000991 | yes | down |
| MsG0680035666.01 | 1.661665212 | 9.66E-06 | yes | up |
| MsG0680035790.01 | 2.224550991 | 0.000188 | yes | up |
| MsG0680035909.01 | -1.059376901 | 0.000244 | yes | down |
| MsG0780035923.01 | 1.475518464 | 0.000503 | yes | up |
| MsG0780035943.01 | 2.603785598 | 0.000583 | yes | up |
| MsG0780036054.01 | -4.363051613 | 3.56E-06 | yes | down |
| MsG0780036139.01 | -1.157926296 | 0.001259 | yes | down |
| MsG0780036173.01 | -7.231731009 | 0.001196 | yes | down |
| MsG0780036197.01 | -3.201223666 | 0.001802 | yes | down |
| MsG0780036206.01 | -3.842699911 | 6.05E-09 | yes | down |
| MsG0780036333.01 | 4.822851836 | 0.00013 | yes | up |
| MsG0780036467.01 | -6.775022453 | 2.15E-08 | yes | down |
| MsG0780036603.01 | -5.702456309 | 0.000635 | yes | down |
| MsG0780036611.01 | -1.300446214 | 0.001117 | yes | down |
| MsG0780036759.01 | -2.917532073 | 0.000244 | yes | down |
| MsG0780036879.01 | -1.673973521 | 3.91E-07 | yes | down |
| MsG0780036880.01 | 1.347296343 | 0.000132 | yes | up |
| MsG0780036882.01 | -1.492509317 | 0.00013 | yes | down |
| MsG0780036951.01 | -2.456710791 | 7.39E-08 | yes | down |
| MsG0780036953.01 | -1.78499712 | 5.67E-08 | yes | down |
| MsG0780036965.01 | 6.248231425 | 0.000786 | yes | up |
| MsG0780037057.01 | -1.061712755 | 0.000513 | yes | down |
| MsG0780037188.01 | -2.064683849 | 0.000283 | yes | down |
| MsG0780037371.01 | -2.165812877 | 4.42E-06 | yes | down |
| MsG0780037513.01 | -2.676078141 | 6.44E-17 | yes | down |
| MsG0780037592.01 | 1.694795678 | 0.000929 | yes | up |
| MsG0780037607.01 | 5.164589867 | 0.001937 | yes | up |
| MsG0780037780.01 | -9.545202171 | 1.24E-12 | yes | down |
| MsG0780037877.01 | 5.642804441 | 0.001326 | yes | up |
| MsG0780037939.01 | -4.342667253 | 0.000102 | yes | down |
| MsG0780037982.01 | 1.071861457 | 0.001357 | yes | up |
| MsG0780038115.01 | -2.625144786 | 0.000204 | yes | down |
| MsG0780038116.01 | -3.32553994 | 0.001892 | yes | down |
| MsG0780038130.01 | -3.548490774 | 0.000475 | yes | down |
| MsG0780038373.01 | -6.40174844 | 9.53E-05 | yes | down |
| MsG0780038376.01 | 5.486926523 | 1.97E-06 | yes | up |
| MsG0780038613.01 | 2.857012664 | 5.64E-08 | yes | up |
| MsG0780038647.01 | -2.177598686 | 0.000517 | yes | down |
| MsG0780038665.01 | -2.92603352 | 1.15E-05 | yes | down |
| MsG0780038753.01 | -2.338662326 | 0.000229 | yes | down |
| MsG0780038803.01 | -1.783268413 | 5.38E-06 | yes | down |
| MsG0780038826.01 | 1.356968514 | 2.32E-05 | yes | up |
| MsG0780038861.01 | -1.339777059 | 2.72E-06 | yes | down |
| MsG0780038914.01 | -3.063077764 | 0.000272 | yes | down |
| MsG0780038937.01 | -1.481175574 | 0.002051 | yes | down |
| MsG0780039096.01 | -4.098419122 | 4.81E-06 | yes | down |
| MsG0780039097.01 | -4.142981651 | 1.07E-05 | yes | down |
| MsG0780039113.01 | -3.243095805 | 0.000233 | yes | down |
| MsG0780039135.01 | -3.868653958 | 0.000275 | yes | down |
| MsG0780039258.01 | -1.402774531 | 2.94E-06 | yes | down |
| MsG0780039388.01 | 1.859028329 | 0.000142 | yes | up |
| MsG0780039499.01 | 2.124540436 | 0.000932 | yes | up |
| MsG0780039503.01 | 3.752816655 | 2.44E-11 | yes | up |
| MsG0780039580.01 | -1.083906453 | 0.000284 | yes | down |
| MsG0780039585.01 | -4.431220747 | 0.00113 | yes | down |
| MsG0780039591.01 | 5.289988135 | 2.23E-06 | yes | up |
| MsG0780039793.01 | -1.432657577 | 0.000435 | yes | down |
| MsG0780039894.01 | 7.172226076 | 1.44E-54 | yes | up |
| MsG0780039923.01 | -3.967111627 | 0.00037 | yes | down |
| MsG0780039973.01 | -2.792399576 | 0.00049 | yes | down |
| MsG0780039993.01 | 3.018996374 | 1.31E-07 | yes | up |
| MsG0780039998.01 | -3.500883639 | 1.87E-05 | yes | down |
| MsG0780040101.01 | -1.135243972 | 0.001253 | yes | down |
| MsG0780040174.01 | -1.010002795 | 0.000416 | yes | down |
| MsG0780040237.01 | -1.376084876 | 0.000408 | yes | down |
| MsG0780040325.01 | -2.962273636 | 0.000421 | yes | down |
| MsG0780040408.01 | 1.480549124 | 4.19E-06 | yes | up |
| MsG0780040510.01 | 1.655279572 | 0.00129 | yes | up |
| MsG0780040654.01 | 1.622452976 | 1.19E-06 | yes | up |
| MsG0780040676.01 | -2.896126881 | 5.85E-05 | yes | down |
| MsG0780040683.01 | -2.4386816 | 1.55E-05 | yes | down |
| MsG0780040738.01 | -2.335846575 | 4.83E-08 | yes | down |
| MsG0780040756.01 | -1.584772571 | 2.90E-05 | yes | down |
| MsG0780040804.01 | -2.467238961 | 0.001201 | yes | down |
| MsG0780040823.01 | -1.100509002 | 7.90E-05 | yes | down |
| MsG0780040980.01 | 2.300193815 | 0.000221 | yes | up |
| MsG0780041022.01 | 2.635999892 | 0.000527 | yes | up |
| MsG0780041053.01 | 1.479569305 | 0.001317 | yes | up |
| MsG0780041062.01 | -1.368930131 | 0.000521 | yes | down |
| MsG0780041078.01 | 1.050294848 | 0.001985 | yes | up |
| MsG0780041082.01 | -1.237477839 | 4.66E-05 | yes | down |
| MsG0780041140.01 | 10.56170072 | 3.89E-13 | yes | up |
| MsG0780041221.01 | -4.460539616 | 0.00095 | yes | down |
| MsG0780041255.01 | -4.202464572 | 0.001428 | yes | down |
| MsG0780041477.01 | 3.262905409 | 7.84E-08 | yes | up |
| MsG0780041516.01 | -1.347237652 | 0.001973 | yes | down |
| MsG0780041533.01 | -1.380784919 | 0.001243 | yes | down |
| MsG0780041648.01 | 1.563596702 | 0.00034 | yes | up |
| MsG0780041677.01 | 2.432770846 | 0.001242 | yes | up |
| MsG0780041688.01 | -1.033222212 | 0.001088 | yes | down |
| MsG0780041696.01 | -3.777801311 | 5.98E-05 | yes | down |
| MsG0780041750.01 | 4.693002928 | 0.000171 | yes | up |
| MsG0780041784.01 | -2.655091053 | 7.00E-09 | yes | down |
| MsG0880041844.01 | -3.06625335 | 9.41E-07 | yes | down |
| MsG0880041863.01 | -2.714651655 | 3.30E-05 | yes | down |
| MsG0880041902.01 | 7.425098516 | 7.31E-07 | yes | up |
| MsG0880041932.01 | 5.914914495 | 4.68E-05 | yes | up |
| MsG0880041970.01 | 2.890550211 | 1.19E-05 | yes | up |
| MsG0880041971.01 | 1.967885909 | 0.000783 | yes | up |
| MsG0880041983.01 | 6.841151515 | 1.19E-22 | yes | up |
| MsG0880041996.01 | 2.204641341 | 2.86E-18 | yes | up |
| MsG0880042001.01 | 3.383573529 | 1.32E-07 | yes | up |
| MsG0880042009.01 | -1.06963143 | 0.000138 | yes | down |
| MsG0880042130.01 | 4.840463304 | 0.000189 | yes | up |
| MsG0880042139.01 | 4.587359669 | 0.000319 | yes | up |
| MsG0880042144.01 | 1.328967857 | 0.000298 | yes | up |
| MsG0880042246.01 | 1.582631028 | 0.000528 | yes | up |
| MsG0880042294.01 | -2.02262635 | 2.93E-06 | yes | down |
| MsG0880042314.01 | -1.780968504 | 4.54E-05 | yes | down |
| MsG0880042338.01 | -2.092069469 | 0.000386 | yes | down |
| MsG0880042368.01 | 1.170546246 | 0.001199 | yes | up |
| MsG0880042478.01 | 4.620289593 | 0.000145 | yes | up |
| MsG0880042495.01 | 2.413619676 | 2.22E-10 | yes | up |
| MsG0880042506.01 | -8.413065797 | 8.13E-06 | yes | down |
| MsG0880042557.01 | -2.936311964 | 6.46E-08 | yes | down |
| MsG0880042581.01 | -1.005867738 | 0.000567 | yes | down |
| MsG0880042613.01 | 2.175015765 | 0.000675 | yes | up |
| MsG0880042714.01 | 1.284589294 | 0.000577 | yes | up |
| MsG0880042794.01 | 1.215235084 | 1.30E-05 | yes | up |
| MsG0880042896.01 | -1.395038266 | 0.000216 | yes | down |
| MsG0880042901.01 | 6.286635307 | 1.82E-06 | yes | up |
| MsG0880042986.01 | -1.350964803 | 0.000877 | yes | down |
| MsG0880042993.01 | -3.830967488 | 0.000159 | yes | down |
| MsG0880042998.01 | 3.921104496 | 7.71E-05 | yes | up |
| MsG0880043020.01 | -2.250112156 | 4.70E-10 | yes | down |
| MsG0880043093.01 | 7.388154885 | 0.000206 | yes | up |
| MsG0880043094.01 | 3.223544548 | 0.000145 | yes | up |
| MsG0880043195.01 | 5.843024547 | 1.57E-14 | yes | up |
| MsG0880043206.01 | 3.565887699 | 1.72E-05 | yes | up |
| MsG0880043343.01 | 1.580440518 | 0.00097 | yes | up |
| MsG0880043363.01 | 2.761560164 | 0.000114 | yes | up |
| MsG0880043483.01 | -2.989026226 | 0.001314 | yes | down |
| MsG0880043495.01 | -1.294915695 | 0.002068 | yes | down |
| MsG0880043550.01 | -6.738604815 | 3.89E-10 | yes | down |
| MsG0880043554.01 | -1.510963062 | 0.002032 | yes | down |
| MsG0880043598.01 | 5.879493316 | 0.000286 | yes | up |
| MsG0880043647.01 | 2.213911775 | 0.000283 | yes | up |
| MsG0880043786.01 | -1.849773654 | 7.99E-05 | yes | down |
| MsG0880043827.01 | 1.983851722 | 0.000152 | yes | up |
| MsG0880043828.01 | 2.20208957 | 0.001533 | yes | up |
| MsG0880043852.01 | 2.86532609 | 2.88E-09 | yes | up |
| MsG0880043916.01 | 2.276240265 | 0.000573 | yes | up |
| MsG0880043930.01 | -4.75843957 | 5.87E-08 | yes | down |
| MsG0880043937.01 | -2.215502592 | 0.00014 | yes | down |
| MsG0880044083.01 | 2.124872648 | 4.75E-07 | yes | up |
| MsG0880044309.01 | -4.529438012 | 0.000236 | yes | down |
| MsG0880044311.01 | -4.309331339 | 7.96E-06 | yes | down |
| MsG0880044373.01 | 5.895929793 | 9.79E-06 | yes | up |
| MsG0880044380.01 | -2.888466223 | 0.000426 | yes | down |
| MsG0880044437.01 | -2.747822492 | 5.57E-06 | yes | down |
| MsG0880044444.01 | -4.997124516 | 2.42E-10 | yes | down |
| MsG0880044567.01 | -2.215939115 | 8.94E-05 | yes | down |
| MsG0880044640.01 | 2.211133325 | 0.000519 | yes | up |
| MsG0880044688.01 | 1.956474224 | 9.60E-09 | yes | up |
| MsG0880044822.01 | -6.428797325 | 0.00026 | yes | down |
| MsG0880045122.01 | -1.664442367 | 0.000367 | yes | down |
| MsG0880045163.01 | 6.642642721 | 0.001074 | yes | up |
| MsG0880045321.01 | -5.041292921 | 1.49E-05 | yes | down |
| MsG0880045339.01 | -4.324818525 | 0.00013 | yes | down |
| MsG0880045386.01 | 1.228130471 | 0.000125 | yes | up |
| MsG0880045416.01 | 1.649511153 | 7.85E-05 | yes | up |
| MsG0880045544.01 | -2.266316474 | 0.00115 | yes | down |
| MsG0880045572.01 | 6.391594624 | 2.39E-09 | yes | up |
| MsG0880045573.01 | 3.627962431 | 0.000475 | yes | up |
| MsG0880045608.01 | 1.507652948 | 1.67E-05 | yes | up |
| MsG0880045651.01 | 6.142760664 | 0.00024 | yes | up |
| MsG0880045861.01 | 2.245225622 | 1.46E-05 | yes | up |
| MsG0880045943.01 | -4.113598978 | 0.000242 | yes | down |
| MsG0880045958.01 | -2.105034459 | 0.000603 | yes | down |
| MsG0880046036.01 | -3.822661699 | 1.98E-05 | yes | down |
| MsG0880046043.01 | 2.344003935 | 0.000542 | yes | up |
| MsG0880046046.01 | 2.861356181 | 1.33E-08 | yes | up |
| MsG0880046050.01 | -2.479896912 | 0.00015 | yes | down |
| MsG0880046126.01 | 1.034194942 | 0.002004 | yes | up |
| MsG0880046140.01 | -7.614474931 | 1.14E-06 | yes | down |
| MsG0880046208.01 | -1.468300891 | 2.41E-05 | yes | down |
| MsG0880046277.01 | 2.073843897 | 0.000448 | yes | up |
| MsG0880046321.01 | -1.255872193 | 8.93E-05 | yes | down |
| MsG0880046327.01 | -1.759789216 | 3.00E-09 | yes | down |
| MsG0880046406.01 | -2.505562071 | 0.001779 | yes | down |
| MsG0880046408.01 | 4.76607874 | 1.11E-06 | yes | up |
| MsG0880046412.01 | 1.215634741 | 0.000847 | yes | up |
| MsG0880046461.01 | -1.848943331 | 5.09E-05 | yes | down |
| MsG0880046499.01 | -1.494952099 | 2.85E-05 | yes | down |
| MsG0880046518.01 | 1.075634231 | 7.62E-05 | yes | up |
| MsG0880046530.01 | 2.400462994 | 9.44E-06 | yes | up |
| MsG0880046575.01 | -1.251320307 | 4.16E-06 | yes | down |
| MsG0880046586.01 | -3.525140149 | 1.43E-05 | yes | down |
| MsG0880046652.01 | -2.016610373 | 4.07E-05 | yes | down |
| MsG0880046760.01 | -1.026943984 | 1.57E-06 | yes | down |
| MsG0880046991.01 | -2.182183798 | 0.001555 | yes | down |
| MsG0880047061.01 | 1.441003374 | 0.000193 | yes | up |
| MsG0880047081.01 | 2.137280684 | 0.000589 | yes | up |
| MsG0880047093.01 | -2.650231113 | 0.001009 | yes | down |
| MsG0880047143.01 | -1.619329357 | 0.000111 | yes | down |
| MsG0880047165.01 | 1.448074587 | 1.86E-06 | yes | up |
| MsG0880047171.01 | -2.011743913 | 0.000289 | yes | down |
| MsG0880047187.01 | -3.26276798 | 3.54E-07 | yes | down |
| MsG0880047250.01 | 8.185846003 | 2.47E-07 | yes | up |
| MsG0880047269.01 | 3.667676876 | 1.25E-08 | yes | up |
| MsG0880047288.01 | -2.677772476 | 0.001893 | yes | down |
| MsG0880047294.01 | 1.114876532 | 0.000286 | yes | up |
| MsG0880047296.01 | 1.673963165 | 0.000214 | yes | up |
| MsG0880047419.01 | -8.339650075 | 2.44E-18 | yes | down |
| MsG0880047514.01 | 3.172249036 | 0.001154 | yes | up |
| MsG0880047531.01 | 1.45584074 | 6.71E-07 | yes | up |
| MsG0880047563.01 | -1.999117172 | 0.000343 | yes | down |
| MsG0880047578.01 | 1.149967626 | 0.001379 | yes | up |
| MsG0880047589.01 | -2.243731525 | 0.001929 | yes | down |
| MsG0880047697.01 | -2.079615705 | 3.71E-11 | yes | down |
