# Supplementary material for: Physiological characteristics and transcriptomic analyses of alfalfa root crown in wintering
Source: Front Plant Sci. 2024 Dec 9;15:1486564. doi: 10.3389/fpls.2024.1486564 (PMC11663680; doi:10.3389/fpls.2024.1486564)
Supplement: Supplementary file 2 [file Table2.docx]

**(TABLE S2)** GO Classification Statistics Table for Gene Sets

| **GO ID** | **Description** | **Term Type** | **num** | **percent** | **Gene_ids** |
| --- | --- | --- | --- | --- | --- |
| GO:0002376 | immune system process | Biological process | 5 | 5/ 878 | MsG0780041688.01;MsG0780038914.01;MsG0780038373.01;MsG0580029467.01;MsG0580029429.01 |
| GO:0065007 | biological regulation | Biological process | 141 | 141/ 878 | MsG0180000284.01;MsG0380016941.01;MsG0680033015.01;MsG0180003243.01;MsG0880047143.01;MsG0880046412.01;MsG0280007911.01;MsG0180004915.01;MsG0280008601.01;MsG0780035923.01;MsG0580029282.01;MsG0580027261.01;MsG0780037939.01;MsG0680034475.01;MsG0180004623.01;MsG0280009582.01;MsG0880047250.01;MsG0680031206.01;MsG0280010916.01;MsG0480023401.01;MsG0580025409.01;MsG0180004830.01;MsG0180004293.01;MsG0580025567.01;MsG0180003993.01;MsG0880041844.01;MsG0880047187.01;MsG0880042714.01;MsG0580029737.01;MsG0580025664.01;MsG0180002138.01;MsG0180001484.01;MsG0680035118.01;MsG0280009717.01;MsG0780039258.01;MsG0380015187.01;MsG0880043916.01;MsG0280008879.01;MsG0680031278.01;MsG0680034797.01;MsG0380015519.01;MsG0880042613.01;MsG0580027139.01;MsG0280009812.01;MsG0480022322.01;MsG0180001019.01;MsG0280007842.01;MsG0880043852.01;MsG0280008334.01;MsG0480023102.01;MsG0480019218.01;MsG0380016948.01;MsG0880046518.01;MsG0780036759.01;MsG0280006932.01;MsG0280007906.01;MsG0580028455.01;MsG0680035017.01;MsG0580027470.01;MsG0580028593.01;MsG0680033003.01;MsG0480019538.01;MsG0180002924.01;MsG0780041688.01;MsG0880046586.01;MsG0880042506.01;MsG0580025514.01;MsG0580030137.01;MsG0780041648.01;MsG0480022053.01;MsG0880044380.01;MsG0380017614.01;MsG0580026026.01;MsG0180005718.01;MsG0580028710.01;MsG0880047165.01;MsG0780039388.01;MsG0480021976.01;MsG0880042986.01;MsG0880047081.01;MsG0680032925.01;MsG0080048527.01;MsG0680035909.01;MsG0380011962.01;MsG0680035493.01;MsG0680030892.01;MsG0380014133.01;MsG0680033830.01;MsG0180003906.01;MsG0680035589.01;MsG0380015135.01;MsG0480022111.01;MsG0280007870.01;MsG0380016368.01;MsG0480018187.01;MsG0680034998.01;MsG0880047288.01;MsG0680035102.01;MsG0480020220.01;MsG0680031277.01;MsG0180003922.01;MsG0280010593.01;MsG0580026003.01;MsG0280009321.01;MsG0780041533.01;MsG0680030604.01;MsG0780036611.01;MsG0880046050.01;MsG0280007427.01;MsG0380015801.01;MsG0480022099.01;MsG0580027113.01;MsG0480023251.01;MsG0880046499.01;MsG0680030949.01;MsG0680032778.01;MsG0080048035.01;MsG0680034723.01;MsG0580024328.01;MsG0680031055.01;MsG0180000842.01;MsG0380015233.01;MsG0680030976.01;MsG0880046652.01;MsG0180005642.01;MsG0180004917.01;MsG0880046208.01;MsG0080048106.01;MsG0780040683.01;MsG0480022512.01;MsG0880043363.01;MsG0180003932.01;MsG0680034110.01;MsG0080048202.01;MsG0780040676.01;MsG0280008756.01;MsG0580025612.01;MsG0480022662.01;MsG0380012807.01;MsG0180003377.01;MsG0680033821.01 |
| GO:0008152 | metabolic process | Biological process | 422 | 422/ 878 | MsG0680030916.01;MsG0780039591.01;MsG0380016941.01;MsG0680033015.01;MsG0380012211.01;MsG0380015326.01;MsG0480020083.01;MsG0580029491.01;MsG0880046461.01;MsG0180003404.01;MsG0580028196.01;MsG0780037939.01;MsG0680031150.01;MsG0780041078.01;MsG0280009582.01;MsG0780040980.01;MsG0480022746.01;MsG0880046575.01;MsG0280007677.01;MsG0480020880.01;MsG0880042478.01;MsG0180000075.01;MsG0180000936.01;MsG0580024551.01;MsG0280008357.01;MsG0480019538.01;MsG0180004921.01;MsG0580027615.01;MsG0680031997.01;MsG0380016853.01;MsG0680031368.01;MsG0280006973.01;MsG0280007699.01;MsG0380016110.01;MsG0780036173.01;MsG0580025468.01;MsG0880045573.01;MsG0080049045.01;MsG0380017684.01;MsG0380016948.01;MsG0480023238.01;MsG0880046518.01;MsG0680034036.01;MsG0280007684.01;MsG0680034868.01;MsG0280007337.01;MsG0280010899.01;MsG0780036759.01;MsG0580024151.01;MsG0580028455.01;MsG0680030918.01;MsG0680034576.01;MsG0280007354.01;MsG0880043093.01;MsG0880046991.01;MsG0380013478.01;MsG0880047294.01;MsG0380015739.01;MsG0780041255.01;MsG0280009541.01;MsG0380011895.01;MsG0780041696.01;MsG0180000147.01;MsG0680035514.01;MsG0880047165.01;MsG0480023627.01;MsG0780039923.01;MsG0380013949.01;MsG0880047296.01;MsG0780038826.01;MsG0280007878.01;MsG0280010702.01;MsG0080048355.01;MsG0480022111.01;MsG0380012869.01;MsG0480022895.01;MsG0380016368.01;MsG0180000942.01;MsG0380012553.01;MsG0380016962.01;MsG0380015095.01;MsG0380015819.01;MsG0780039113.01;MsG0480020344.01;MsG0480019829.01;MsG0780038115.01;MsG0380017683.01;MsG0880043343.01;MsG0480021587.01;MsG0480023689.01;MsG0280010856.01;MsG0580027113.01;MsG0280008988.01;MsG0480023549.01;MsG0180000984.01;MsG0380017699.01;MsG0180000962.01;MsG0180003808.01;MsG0180000976.01;MsG0880042495.01;MsG0480022512.01;MsG0280010648.01;MsG0480022662.01;MsG0880045163.01;MsG0880047563.01;MsG0380013796.01;MsG0480022664.01;MsG0280009139.01;MsG0380015858.01;MsG0380016458.01;MsG0480019426.01;MsG0180002793.01;MsG0180003753.01;MsG0280008643.01;MsG0880047578.01;MsG0880042896.01;MsG0780036054.01;MsG0580027749.01;MsG0680033932.01;MsG0580027261.01;MsG0880046277.01;MsG0680031966.01;MsG0480021267.01;MsG0880047531.01;MsG0480021529.01;MsG0780037982.01;MsG0380015414.01;MsG0880041983.01;MsG0380013761.01;MsG0780039793.01;MsG0680034040.01;MsG0480022053.01;MsG0280010428.01;MsG0180001480.01;MsG0580024656.01;MsG0580029429.01;MsG0880047514.01;MsG0380014438.01;MsG0680034506.01;MsG0680035323.01;MsG0280010728.01;MsG0280006942.01;MsG0480023416.01;MsG0880045651.01;MsG0580028614.01;MsG0680032380.01;MsG0780041053.01;MsG0380015328.01;MsG0480022177.01;MsG0680031284.01;MsG0280008720.01;MsG0880044373.01;MsG0080048638.01;MsG0580028593.01;MsG0380017793.01;MsG0880041902.01;MsG0680035666.01;MsG0180002272.01;MsG0380011483.01;MsG0780040174.01;MsG0580030137.01;MsG0580028422.01;MsG0380014422.01;MsG0380015944.01;MsG0480023550.01;MsG0180004103.01;MsG0680031280.01;MsG0580027449.01;MsG0180002030.01;MsG0880046321.01;MsG0580029315.01;MsG0780041750.01;MsG0680030529.01;MsG0880045572.01;MsG0880046126.01;MsG0780039580.01;MsG0680030892.01;MsG0380015722.01;MsG0780040804.01;MsG0680035589.01;MsG0380015135.01;MsG0580030133.01;MsG0880042338.01;MsG0280010598.01;MsG0680032095.01;MsG0480022724.01;MsG0380015498.01;MsG0480020392.01;MsG0580024522.01;MsG0780039135.01;MsG0880043827.01;MsG0880042246.01;MsG0280006918.01;MsG0480018982.01;MsG0380013547.01;MsG0380012272.01;MsG0680030342.01;MsG0580030135.01;MsG0180000577.01;MsG0480023604.01;MsG0080049160.01;MsG0680031055.01;MsG0880046327.01;MsG0380017707.01;MsG0180000068.01;MsG0580024168.01;MsG0180005642.01;MsG0080048106.01;MsG0780041477.01;MsG0380017598.01;MsG0280006479.01;MsG0880046760.01;MsG0680031403.01;MsG0580025906.01;MsG0380014047.01;MsG0380017247.01;MsG0180002226.01;MsG0680034507.01;MsG0480021692.01;MsG0680034513.01;MsG0280006415.01;MsG0080048805.01;MsG0380017382.01;MsG0680034475.01;MsG0180004623.01;MsG0180005142.01;MsG0180001068.01;MsG0880042314.01;MsG0580027523.01;MsG0080048322.01;MsG0480020534.01;MsG0780039499.01;MsG0580025567.01;MsG0880043206.01;MsG0680030556.01;MsG0580029737.01;MsG0580025586.01;MsG0380015691.01;MsG0480022039.01;MsG0480021891.01;MsG0780041062.01;MsG0480023229.01;MsG0880047697.01;MsG0880044640.01;MsG0280009992.01;MsG0780038130.01;MsG0780039096.01;MsG0380015499.01;MsG0180000395.01;MsG0480020220.01;MsG0580025261.01;MsG0880042294.01;MsG0780037188.01;MsG0680031962.01;MsG0680030953.01;MsG0580027470.01;MsG0480020366.01;MsG0580027605.01;MsG0880043828.01;MsG0580027661.01;MsG0680035353.01;MsG0680034225.01;MsG0480020492.01;MsG0580026026.01;MsG0180005308.01;MsG0780039993.01;MsG0780040823.01;MsG0480021976.01;MsG0580027678.01;MsG0080048527.01;MsG0580029467.01;MsG0280008349.01;MsG0380011962.01;MsG0280006919.01;MsG0280008350.01;MsG0880046046.01;MsG0680034510.01;MsG0580024217.01;MsG0280010298.01;MsG0680034028.01;MsG0780036879.01;MsG0280007827.01;MsG0180004622.01;MsG0380014133.01;MsG0180005687.01;MsG0580024333.01;MsG0280011162.01;MsG0880045122.01;MsG0880041996.01;MsG0180005253.01;MsG0580027221.01;MsG0680034516.01;MsG0480023682.01;MsG0380018047.01;MsG0880043094.01;MsG0780040756.01;MsG0780037057.01;MsG0480022032.01;MsG0280007923.01;MsG0880041971.01;MsG0580027832.01;MsG0580029697.01;MsG0180003377.01;MsG0280007708.01;MsG0780036197.01;MsG0680034515.01;MsG0280006533.01;MsG0780040237.01;MsG0780038116.01;MsG0380012513.01;MsG0380017926.01;MsG0580030199.01;MsG0880044083.01;MsG0880044444.01;MsG0480023401.01;MsG0380015544.01;MsG0780041082.01;MsG0880045544.01;MsG0780039388.01;MsG0780040408.01;MsG0180002438.01;MsG0480020827.01;MsG0880043483.01;MsG0080047999.01;MsG0480019502.01;MsG0280010661.01;MsG0180000970.01;MsG0280007781.01;MsG0180004915.01;MsG0880047061.01;MsG0380017913.01;MsG0680032431.01;MsG0180003992.01;MsG0280010769.01;MsG0880042001.01;MsG0880042139.01;MsG0880041970.01;MsG0280011054.01;MsG0380013973.01;MsG0580026705.01;MsG0380016341.01;MsG0180000408.01;MsG0480018099.01;MsG0280008334.01;MsG0880045321.01;MsG0380013971.01;MsG0380016080.01;MsG0780041022.01;MsG0680034376.01;MsG0380012788.01;MsG0480018991.01;MsG0480023742.01;MsG0380018025.01;MsG0680034032.01;MsG0780036467.01;MsG0680034499.01;MsG0580027828.01;MsG0580029519.01;MsG0280008072.01;MsG0880042130.01;MsG0180004130.01;MsG0480023026.01;MsG0180002924.01;MsG0780041688.01;MsG0580025514.01;MsG0180003993.01;MsG0180003484.01;MsG0680030558.01;MsG0680032201.01;MsG0680034998.01;MsG0180005718.01;MsG0580028710.01;MsG0480022910.01;MsG0380012035.01;MsG0380017404.01;MsG0180006081.01;MsG0180006211.01;MsG0380017993.01;MsG0780038647.01;MsG0680034495.01;MsG0680031996.01;MsG0280008597.01;MsG0380016028.01;MsG0180000271.01;MsG0480020259.01;MsG0580026594.01;MsG0480018496.01;MsG0680032245.01;MsG0180000437.01;MsG0480018187.01;MsG0380015519.01;MsG0680033485.01;MsG0180005819.01;MsG0280008352.01;MsG0380013974.01;MsG0880047250.01;MsG0580026003.01;MsG0580024855.01;MsG0680031087.01;MsG0280010311.01;MsG0180004385.01;MsG0180005005.01;MsG0580028107.01;MsG0380014847.01;MsG0780036611.01;MsG0280007427.01;MsG0280008107.01;MsG0880047419.01;MsG0080048202.01;MsG0480022099.01;MsG0180001856.01;MsG0180000125.01;MsG0880046499.01;MsG0880046140.01;MsG0480020134.01;MsG0680031545.01;MsG0780038937.01;MsG0580027139.01;MsG0580028496.01;MsG0180000751.01;MsG0280008756.01;MsG0580026176.01 |
| GO:0051704 | multi-organism process | Biological process | 34 | 34/ 878 | MsG0280010378.01;MsG0480021600.01;MsG0180004978.01;MsG0580029467.01;MsG0780041688.01;MsG0580027513.01;MsG0180001480.01;MsG0180005687.01;MsG0780038373.01;MsG0680032431.01;MsG0580029429.01;MsG0380014410.01;MsG0380012035.01;MsG0480021599.01;MsG0780039923.01;MsG0880045573.01;MsG0780038826.01;MsG0780038914.01;MsG0880045572.01;MsG0280006973.01;MsG0180004091.01;MsG0580024328.01;MsG0680034036.01;MsG0180004027.01;MsG0380014748.01;MsG0480022895.01;MsG0880046208.01;MsG0680032833.01;MsG0680034032.01;MsG0480021761.01;MsG0180005819.01;MsG0580025586.01;MsG0680034028.01;MsG0680033003.01 |
| GO:0040011 | locomotion | Biological process | 1 | 1/ 878 | MsG0180004027.01 |
| GO:0022414 | reproductive process | Biological process | 23 | 23/ 878 | MsG0680033015.01;MsG0380014133.01;MsG0680032551.01;MsG0680030604.01;MsG0280007427.01;MsG0680035589.01;MsG0280010648.01;MsG0880044640.01;MsG0680034376.01;MsG0380012788.01;MsG0480018991.01;MsG0480022662.01;MsG0180003753.01;MsG0280010593.01;MsG0880046652.01;MsG0080048106.01;MsG0880044373.01;MsG0280010298.01;MsG0480021761.01;MsG0280010551.01;MsG0480020220.01;MsG0680032546.01;MsG0380014233.01 |
| GO:0000003 | reproduction | Biological process | 1 | 1/ 878 | MsG0480021761.01 |
| GO:0071840 | cellular component organization or biogenesis | Biological process | 78 | 78/ 878 | MsG0480019538.01;MsG0180004130.01;MsG0880047143.01;MsG0880043363.01;MsG0480021655.01;MsG0380017788.01;MsG0680033015.01;MsG0880046586.01;MsG0380014133.01;MsG0780040174.01;MsG0580030137.01;MsG0780040237.01;MsG0580028958.01;MsG0680031368.01;MsG0580028957.01;MsG0280010593.01;MsG0480021913.01;MsG0480022346.01;MsG0280007990.01;MsG0580028710.01;MsG0880047578.01;MsG0780040756.01;MsG0180004741.01;MsG0180003993.01;MsG0180000408.01;MsG0780039258.01;MsG0480022910.01;MsG0480023604.01;MsG0480022848.01;MsG0480023248.01;MsG0380013949.01;MsG0480022345.01;MsG0680033485.01;MsG0080048716.01;MsG0680030949.01;MsG0280009582.01;MsG0780037939.01;MsG0480023550.01;MsG0280010661.01;MsG0680034376.01;MsG0380012788.01;MsG0480020134.01;MsG0480023114.01;MsG0680030892.01;MsG0280009072.01;MsG0480020220.01;MsG0180000472.01;MsG0780038753.01;MsG0480023627.01;MsG0580026206.01;MsG0480023916.01;MsG0680035589.01;MsG0380015135.01;MsG0580024974.01;MsG0580027139.01;MsG0580028455.01;MsG0680031284.01;MsG0380012869.01;MsG0480018324.01;MsG0580025567.01;MsG0080048106.01;MsG0380015414.01;MsG0680032019.01;MsG0180001484.01;MsG0680031962.01;MsG0280007870.01;MsG0680032551.01;MsG0280008107.01;MsG0480020151.01;MsG0480020818.01;MsG0880046760.01;MsG0180003711.01;MsG0480022662.01;MsG0680032546.01;MsG0680034852.01;MsG0380014233.01;MsG0680035494.01;MsG0480022342.01 |
| GO:0009987 | cellular process | Biological process | 421 | 421/ 878 | MsG0780039591.01;MsG0380016941.01;MsG0680033015.01;MsG0380012211.01;MsG0380015326.01;MsG0480020083.01;MsG0880046461.01;MsG0180003404.01;MsG0580028196.01;MsG0780037939.01;MsG0480022111.01;MsG0680031150.01;MsG0780041078.01;MsG0280009582.01;MsG0780040980.01;MsG0480022746.01;MsG0180004027.01;MsG0880046575.01;MsG0580025409.01;MsG0280007677.01;MsG0480020880.01;MsG0180004293.01;MsG0180000075.01;MsG0180003711.01;MsG0280008334.01;MsG0180000936.01;MsG0580024551.01;MsG0480019538.01;MsG0180004921.01;MsG0580027615.01;MsG0780039258.01;MsG0680031997.01;MsG0380016853.01;MsG0680031368.01;MsG0280007699.01;MsG0780036173.01;MsG0880045573.01;MsG0080049045.01;MsG0380017684.01;MsG0380016948.01;MsG0480023627.01;MsG0880046518.01;MsG0680034036.01;MsG0280007684.01;MsG0680034868.01;MsG0280007337.01;MsG0780036759.01;MsG0280007906.01;MsG0580028455.01;MsG0680030918.01;MsG0680034576.01;MsG0680035017.01;MsG0280007354.01;MsG0880046991.01;MsG0380013478.01;MsG0880047294.01;MsG0880045544.01;MsG0880046586.01;MsG0280009541.01;MsG0680032551.01;MsG0380011895.01;MsG0180000147.01;MsG0680035514.01;MsG0880047165.01;MsG0380017404.01;MsG0180004126.01;MsG0880047081.01;MsG0380013949.01;MsG0880047296.01;MsG0680032925.01;MsG0780038826.01;MsG0680035909.01;MsG0280007878.01;MsG0280010702.01;MsG0080048355.01;MsG0680030976.01;MsG0580024974.01;MsG0380012869.01;MsG0480022895.01;MsG0380016368.01;MsG0180000942.01;MsG0680035102.01;MsG0380016962.01;MsG0380015095.01;MsG0680031277.01;MsG0180003922.01;MsG0780039113.01;MsG0380017788.01;MsG0480020344.01;MsG0780041533.01;MsG0880042581.01;MsG0880043343.01;MsG0480021587.01;MsG0280007990.01;MsG0880042495.01;MsG0380017683.01;MsG0480022848.01;MsG0580027113.01;MsG0680030949.01;MsG0480023549.01;MsG0680034723.01;MsG0180000984.01;MsG0380017699.01;MsG0180000962.01;MsG0480022599.01;MsG0180003808.01;MsG0180000976.01;MsG0680032019.01;MsG0480022512.01;MsG0780040676.01;MsG0280010648.01;MsG0480022662.01;MsG0880045163.01;MsG0880047563.01;MsG0380013796.01;MsG0480022664.01;MsG0280010856.01;MsG0380016458.01;MsG0180002793.01;MsG0180003753.01;MsG0680031962.01;MsG0880047578.01;MsG0880042896.01;MsG0580027749.01;MsG0680033932.01;MsG0580029282.01;MsG0580027261.01;MsG0680031966.01;MsG0480021267.01;MsG0880047531.01;MsG0480021529.01;MsG0780037982.01;MsG0880042478.01;MsG0380015414.01;MsG0380013761.01;MsG0780039793.01;MsG0280009139.01;MsG0380018047.01;MsG0680034998.01;MsG0580024656.01;MsG0580029429.01;MsG0680031278.01;MsG0480022346.01;MsG0880047514.01;MsG0680035323.01;MsG0280010728.01;MsG0280006942.01;MsG0480023416.01;MsG0880045651.01;MsG0580028614.01;MsG0680032380.01;MsG0780041053.01;MsG0380015328.01;MsG0680031284.01;MsG0280008720.01;MsG0880044373.01;MsG0080048638.01;MsG0580028593.01;MsG0480020151.01;MsG0380017793.01;MsG0880041902.01;MsG0680035666.01;MsG0180002272.01;MsG0380011483.01;MsG0780040174.01;MsG0580030137.01;MsG0780038937.01;MsG0380014422.01;MsG0380015944.01;MsG0480023550.01;MsG0180004103.01;MsG0680031280.01;MsG0580027449.01;MsG0180002030.01;MsG0880046321.01;MsG0580029315.01;MsG0780041750.01;MsG0680030529.01;MsG0880045572.01;MsG0880046126.01;MsG0780039580.01;MsG0680030892.01;MsG0780040804.01;MsG0180003906.01;MsG0680035589.01;MsG0480018324.01;MsG0880042338.01;MsG0280010598.01;MsG0680032095.01;MsG0680032536.01;MsG0780039096.01;MsG0480020392.01;MsG0480020220.01;MsG0780039135.01;MsG0880043827.01;MsG0180000472.01;MsG0280006918.01;MsG0480018982.01;MsG0380013547.01;MsG0380012272.01;MsG0780038753.01;MsG0680030342.01;MsG0180000577.01;MsG0480023604.01;MsG0080049160.01;MsG0680031055.01;MsG0180000842.01;MsG0880046327.01;MsG0480023916.01;MsG0180000068.01;MsG0080048106.01;MsG0580029697.01;MsG0380017598.01;MsG0280006479.01;MsG0880046760.01;MsG0680031403.01;MsG0680032546.01;MsG0580025906.01;MsG0380014233.01;MsG0380017247.01;MsG0180002226.01;MsG0880047143.01;MsG0580026206.01;MsG0280007911.01;MsG0280006415.01;MsG0080048805.01;MsG0380015135.01;MsG0680034852.01;MsG0380017382.01;MsG0680034475.01;MsG0180004623.01;MsG0180001068.01;MsG0880042314.01;MsG0580027523.01;MsG0180004830.01;MsG0080048322.01;MsG0480020534.01;MsG0580025567.01;MsG0880043206.01;MsG0880042714.01;MsG0580029737.01;MsG0580025586.01;MsG0380015691.01;MsG0480022039.01;MsG0180001484.01;MsG0680035118.01;MsG0480021891.01;MsG0480023248.01;MsG0580025674.01;MsG0780041062.01;MsG0880042613.01;MsG0480022724.01;MsG0280010551.01;MsG0880044640.01;MsG0280009992.01;MsG0880047250.01;MsG0380015499.01;MsG0780036953.01;MsG0780036951.01;MsG0180000395.01;MsG0780040823.01;MsG0580025261.01;MsG0880042294.01;MsG0280010607.01;MsG0680030953.01;MsG0580027470.01;MsG0580027605.01;MsG0480020939.01;MsG0580027661.01;MsG0680035353.01;MsG0680034225.01;MsG0480020492.01;MsG0580026026.01;MsG0180005308.01;MsG0780039993.01;MsG0480022345.01;MsG0480021976.01;MsG0580024151.01;MsG0880042986.01;MsG0080048527.01;MsG0580029467.01;MsG0380011962.01;MsG0680035493.01;MsG0280006919.01;MsG0480023114.01;MsG0880046046.01;MsG0680034510.01;MsG0480022342.01;MsG0280010298.01;MsG0680034028.01;MsG0780036879.01;MsG0280007827.01;MsG0180004622.01;MsG0380014133.01;MsG0180005687.01;MsG0580024333.01;MsG0880045122.01;MsG0180005253.01;MsG0580027221.01;MsG0480023682.01;MsG0280010428.01;MsG0880044437.01;MsG0480020818.01;MsG0780040756.01;MsG0780037057.01;MsG0880043828.01;MsG0480022032.01;MsG0180003932.01;MsG0580027832.01;MsG0780037780.01;MsG0180003377.01;MsG0280007708.01;MsG0280010916.01;MsG0880042009.01;MsG0780040237.01;MsG0380012513.01;MsG0380017926.01;MsG0580030199.01;MsG0880044083.01;MsG0880044444.01;MsG0480023401.01;MsG0380015544.01;MsG0780041082.01;MsG0780039388.01;MsG0180002438.01;MsG0480020827.01;MsG0080047999.01;MsG0480019502.01;MsG0280010661.01;MsG0480021913.01;MsG0680031206.01;MsG0180000970.01;MsG0280007781.01;MsG0180004915.01;MsG0880047061.01;MsG0880041844.01;MsG0380014276.01;MsG0180004741.01;MsG0480021655.01;MsG0280010769.01;MsG0680034495.01;MsG0880042139.01;MsG0880041970.01;MsG0680034797.01;MsG0580027139.01;MsG0380016341.01;MsG0180000408.01;MsG0480018099.01;MsG0880043852.01;MsG0880045321.01;MsG0380013971.01;MsG0380016080.01;MsG0480019218.01;MsG0680034376.01;MsG0380012788.01;MsG0480018991.01;MsG0680034032.01;MsG0380015819.01;MsG0580027828.01;MsG0580029519.01;MsG0680033003.01;MsG0880042130.01;MsG0180004130.01;MsG0380017707.01;MsG0180002924.01;MsG0780041688.01;MsG0580025514.01;MsG0180003993.01;MsG0180003484.01;MsG0680030558.01;MsG0480022053.01;MsG0880044380.01;MsG0380017606.01;MsG0580028710.01;MsG0680035494.01;MsG0480022910.01;MsG0880042506.01;MsG0180006081.01;MsG0180006211.01;MsG0380017993.01;MsG0780038647.01;MsG0680031996.01;MsG0280008597.01;MsG0180000271.01;MsG0280010593.01;MsG0580026594.01;MsG0680032245.01;MsG0280007870.01;MsG0480018187.01;MsG0380015519.01;MsG0680033485.01;MsG0180005819.01;MsG0580026003.01;MsG0580024855.01;MsG0680031087.01;MsG0280010311.01;MsG0180004385.01;MsG0180005005.01;MsG0580028107.01;MsG0380014847.01;MsG0780036611.01;MsG0280007427.01;MsG0280008107.01;MsG0780036197.01;MsG0080048202.01;MsG0480022099.01;MsG0780036882.01;MsG0180001856.01;MsG0180000125.01;MsG0880046499.01;MsG0080048716.01;MsG0880046140.01;MsG0480020134.01;MsG0480023742.01;MsG0280009072.01;MsG0480020941.01;MsG0480018496.01;MsG0880043363.01;MsG0180000751.01;MsG0280008756.01;MsG0580026176.01 |
| GO:0032502 | developmental process | Biological  process | 27 | 27/ 878 | MsG0180004094.01;MsG0380017614.01;MsG0580027513.01;MsG0180004102.01;MsG0780037371.01;MsG0680030604.01;MsG0280007427.01;MsG0880045386.01;MsG0180004104.01;MsG0780038613.01;MsG0480021976.01;MsG0880045416.01;MsG0180001856.01;MsG0680035909.01;MsG0680034376.01;MsG0480023627.01;MsG0480018991.01;MsG0580026206.01;MsG0280010593.01;MsG0780038753.01;MsG0480022895.01;MsG0080048716.01;MsG0880043363.01;MsG0180004099.01;MsG0480021761.01;MsG0280010551.01;MsG0380016962.01 |
| GO:0032501 | multicellular organismal process | Biological  process | 7 | 7/ 878 | MsG0480023627.01;MsG0380016962.01;MsG0480018991.01;MsG0280010551.01;MsG0180001856.01;MsG0680030604.01;MsG0580025683.01 |
| GO:0040007 | growth | Biological  process | 2 | 2/ 878 | MsG0680034376.01;MsG0480021976.01 |
| GO:0048511 | rhythmic process | Biological  process | 2 | 2/ 878 | MsG0680032536.01;MsG0180005005.01 |
| GO:0051179 | localization | Biological  process | 70 | 70/ 878 | MsG0180001484.01;MsG0480020896.01;MsG0180005151.01;MsG0780039258.01;MsG0880046586.01;MsG0480020913.01;MsG0880046406.01;MsG0880042506.01;MsG0680035560.01;MsG0780041516.01;MsG0080047868.01;MsG0280011470.01;MsG0780039973.01;MsG0880044309.01;MsG0880047093.01;MsG0580025674.01;MsG0180000993.01;MsG0480023819.01;MsG0180002325.01;MsG0380017008.01;MsG0380011962.01;MsG0680035323.01;MsG0180005298.01;MsG0480022900.01;MsG0380015801.01;MsG0480022099.01;MsG0580029282.01;MsG0880043495.01;MsG0680034814.01;MsG0580029953.01;MsG0480022515.01;MsG0480023248.01;MsG0780040510.01;MsG0380016977.01;MsG0380015825.01;MsG0880046408.01;MsG0480023630.01;MsG0880041844.01;MsG0280007212.01;MsG0880046652.01;MsG0280010661.01;MsG0380014276.01;MsG0280010864.01;MsG0080049160.01;MsG0180000842.01;MsG0280010607.01;MsG0780041677.01;MsG0180004027.01;MsG0480023916.01;MsG0880043647.01;MsG0480018324.01;MsG0280011027.01;MsG0580028238.01;MsG0680030677.01;MsG0880043598.01;MsG0280011288.01;MsG0880047143.01;MsG0780040738.01;MsG0880043020.01;MsG0880044311.01;MsG0580025659.01;MsG0280007478.01;MsG0480020151.01;MsG0680030746.01;MsG0580025586.01;MsG0380012411.01;MsG0480023969.01;MsG0880041902.01;MsG0380012807.01;MsG0780037780.01 |
| GO:0098754 | detoxification | Biological  process | 1 | 1/ 878 | MsG0380016458.01 |
| GO:0019740 | nitrogen utilization | Biological  process | 1 | 1/ 878 | MsG0080048202.01 |
| GO:0050896 | response to stimulus | Biological  process | 121 | 121/ 878 | MsG0180004978.01;MsG0680033015.01;MsG0680032833.01;MsG0280007911.01;MsG0680031814.01;MsG0280010593.01;MsG0380015544.01;MsG0780039923.01;MsG0780037939.01;MsG0880043550.01;MsG0180004623.01;MsG0780041078.01;MsG0280009582.01;MsG0680032431.01;MsG0180004110.01;MsG0180000075.01;MsG0180003993.01;MsG0880042714.01;MsG0280008334.01;MsG0580025586.01;MsG0680035017.01;MsG0480022039.01;MsG0480019538.01;MsG0680035118.01;MsG0780038937.01;MsG0680034495.01;MsG0180001480.01;MsG0180004801.01;MsG0280007677.01;MsG0580029429.01;MsG0480022346.01;MsG0680034797.01;MsG0480023229.01;MsG0880042613.01;MsG0280009812.01;MsG0880045573.01;MsG0380012197.01;MsG0880043852.01;MsG0680035102.01;MsG0780036953.01;MsG0480019218.01;MsG0280010728.01;MsG0780036951.01;MsG0480023238.01;MsG0480023416.01;MsG0880046518.01;MsG0680034036.01;MsG0280007684.01;MsG0680034723.01;MsG0680034032.01;MsG0180004866.01;MsG0880043093.01;MsG0180004091.01;MsG0480020939.01;MsG0680033003.01;MsG0180002272.01;MsG0180000271.01;MsG0380012194.01;MsG0380014410.01;MsG0180002924.01;MsG0780041688.01;MsG0580030137.01;MsG0480023761.01;MsG0680034998.01;MsG0580024405.01;MsG0580028710.01;MsG0880047165.01;MsG0380012035.01;MsG0180000472.01;MsG0480021976.01;MsG0280007906.01;MsG0880042986.01;MsG0880047081.01;MsG0680032925.01;MsG0080048527.01;MsG0780038914.01;MsG0880045572.01;MsG0280007878.01;MsG0380011962.01;MsG0280006550.01;MsG0680030892.01;MsG0680030976.01;MsG0680035589.01;MsG0380014748.01;MsG0380015135.01;MsG0380012869.01;MsG0480022895.01;MsG0480022342.01;MsG0480022724.01;MsG0180005819.01;MsG0880047288.01;MsG0780038826.01;MsG0480020220.01;MsG0680034028.01;MsG0380015095.01;MsG0280010378.01;MsG0480021600.01;MsG0580029467.01;MsG0480022345.01;MsG0380014133.01;MsG0180005687.01;MsG0480018982.01;MsG0780038373.01;MsG0380017607.01;MsG0280010856.01;MsG0880043937.01;MsG0780036197.01;MsG0480021599.01;MsG0280010428.01;MsG0280006973.01;MsG0480023606.01;MsG0580024328.01;MsG0480023742.01;MsG0480022599.01;MsG0480020941.01;MsG0880046208.01;MsG0080048106.01;MsG0780041477.01;MsG0780040676.01;MsG0480022662.01;MsG0280010916.01 |
| GO:0031974 | membrane-enclosed lumen | Cellular component | 8 | 8/ 878 | MsG0880042009.01;MsG0580027139.01;MsG0780038861.01;MsG0480022032.01;MsG0580029047.01;MsG0380014276.01;MsG0380017606.01;MsG0380016977.01 |
| GO:0032991 | protein-containing complex | Cellular component | 90 | 90/ 878 | MsG0380014047.01;MsG0380016941.01;MsG0580028107.01;MsG0680033015.01;MsG0180003243.01;MsG0680031150.01;MsG0180002226.01;MsG0780040237.01;MsG0780041516.01;MsG0880044444.01;MsG0680031962.01;MsG0880045386.01;MsG0880042896.01;MsG0180002438.01;MsG0580029953.01;MsG0380017382.01;MsG0280007781.01;MsG0880046575.01;MsG0480023401.01;MsG0580025409.01;MsG0180004293.01;MsG0180000075.01;MsG0180003711.01;MsG0380017793.01;MsG0180004921.01;MsG0680034495.01;MsG0680035560.01;MsG0680031368.01;MsG0380013973.01;MsG0580026705.01;MsG0180000408.01;MsG0280009812.01;MsG0780041677.01;MsG0580027088.01;MsG0880047250.01;MsG0380013971.01;MsG0380016080.01;MsG0580028238.01;MsG0380016948.01;MsG0480023627.01;MsG0480018991.01;MsG0580024151.01;MsG0480023416.01;MsG0880047143.01;MsG0080048638.01;MsG0480023969.01;MsG0880041902.01;MsG0180004130.01;MsG0780040174.01;MsG0680035494.01;MsG0680035514.01;MsG0480022910.01;MsG0480021976.01;MsG0880045416.01;MsG0180004126.01;MsG0880046321.01;MsG0380013949.01;MsG0580027678.01;MsG0480020392.01;MsG0580028957.01;MsG0680035493.01;MsG0680035589.01;MsG0380012869.01;MsG0680032925.01;MsG0680032536.01;MsG0380017155.01;MsG0380013547.01;MsG0080047868.01;MsG0380017788.01;MsG0780040823.01;MsG0180004622.01;MsG0380014133.01;MsG0480018982.01;MsG0580028958.01;MsG0280011027.01;MsG0480023689.01;MsG0480020134.01;MsG0080049160.01;MsG0780040756.01;MsG0180003920.01;MsG0380015233.01;MsG0480023916.01;MsG0080048106.01;MsG0780037780.01;MsG0480022512.01;MsG0380017598.01;MsG0880046760.01;MsG0480022662.01;MsG0380014233.01;MsG0180003377.01 |
| GO:0044425 | membrane part | Cellular component | 279 | 279/ 878 | MsG0580026594.01;MsG0680030916.01;MsG0180004978.01;MsG0380013796.01;MsG0380012211.01;MsG0280006533.01;MsG0480022664.01;MsG0780040237.01;MsG0780041516.01;MsG0280011470.01;MsG0880045861.01;MsG0280007781.01;MsG0280006468.01;MsG0480019426.01;MsG0080048527.01;MsG0480023819.01;MsG0480021692.01;MsG0680031814.01;MsG0280009054.01;MsG0880045321.01;MsG0280008643.01;MsG0880047578.01;MsG0880042896.01;MsG0780038613.01;MsG0580024522.01;MsG0580029282.01;MsG0680034814.01;MsG0580029953.01;MsG0780037939.01;MsG0880044311.01;MsG0880046277.01;MsG0680034475.01;MsG0380015862.01;MsG0880043598.01;MsG0280009992.01;MsG0480020880.01;MsG0880044640.01;MsG0380014933.01;MsG0380011745.01;MsG0280010864.01;MsG0180001609.01;MsG0880042478.01;MsG0580027523.01;MsG0880042557.01;MsG0480022746.01;MsG0180004027.01;MsG0280008764.01;MsG0780040654.01;MsG0580025409.01;MsG0880047061.01;MsG0780039499.01;MsG0180004293.01;MsG0280011288.01;MsG0180000075.01;MsG0280008334.01;MsG0180005382.01;MsG0880044309.01;MsG0880041844.01;MsG0380015148.01;MsG0780037513.01;MsG0380013761.01;MsG0680030558.01;MsG0480022639.01;MsG0380015825.01;MsG0180000936.01;MsG0580024401.01;MsG0580024551.01;MsG0380015499.01;MsG0180000125.01;MsG0880042901.01;MsG0280007883.01;MsG0580028422.01;MsG0680034225.01;MsG0580025670.01;MsG0780039258.01;MsG0480023248.01;MsG0480020913.01;MsG0780037057.01;MsG0580028956.01;MsG0680031997.01;MsG0380017791.01;MsG0580029429.01;MsG0180002936.01;MsG0580025674.01;MsG0880041863.01;MsG0580028238.01;MsG0180004917.01;MsG0780037877.01;MsG0580026705.01;MsG0380016110.01;MsG0280010551.01;MsG0280009812.01;MsG0580025468.01;MsG0680035790.01;MsG0480022515.01;MsG0380015498.01;MsG0880044083.01;MsG0080048035.01;MsG0880043852.01;MsG0380014438.01;MsG0380013971.01;MsG0680033485.01;MsG0180005121.01;MsG0680035323.01;MsG0180005308.01;MsG0280010728.01;MsG0180005418.01;MsG0580025586.01;MsG0480018745.01;MsG0580025833.01;MsG0880046518.01;MsG0880045651.01;MsG0680034036.01;MsG0380018054.01;MsG0680032380.01;MsG0280007337.01;MsG0780041053.01;MsG0180002325.01;MsG0280010248.01;MsG0780039894.01;MsG0680034576.01;MsG0180000942.01;MsG0780036467.01;MsG0880047563.01;MsG0880044373.01;MsG0680034499.01;MsG0380012411.01;MsG0880042368.01;MsG0080048992.01;MsG0580027828.01;MsG0180004091.01;MsG0580028614.01;MsG0180000452.01;MsG0180000976.01;MsG0280007481.01;MsG0780035943.01;MsG0680035666.01;MsG0580024996.01;MsG0880042130.01;MsG0580027803.01;MsG0480023026.01;MsG0080048716.01;MsG0180005151.01;MsG0880047294.01;MsG0880046586.01;MsG0780037607.01;MsG0580027661.01;MsG0180003484.01;MsG0680032431.01;MsG0680030746.01;MsG0880044380.01;MsG0680031105.01;MsG0680034914.01;MsG0380015944.01;MsG0780036206.01;MsG0180002931.01;MsG0180000147.01;MsG0380017993.01;MsG0680035514.01;MsG0880042506.01;MsG0680031280.01;MsG0580027449.01;MsG0180002030.01;MsG0180001786.01;MsG0780040510.01;MsG0880047143.01;MsG0680034998.01;MsG0880047296.01;MsG0780038826.01;MsG0680030529.01;MsG0680035909.01;MsG0580029467.01;MsG0480023630.01;MsG0280010802.01;MsG0280007878.01;MsG0680034852.01;MsG0180000993.01;MsG0680030677.01;MsG0680031996.01;MsG0880042998.01;MsG0280006919.01;MsG0280006550.01;MsG0080048355.01;MsG0680032778.01;MsG0380016028.01;MsG0780040804.01;MsG0880046046.01;MsG0580026206.01;MsG0380011666.01;MsG0380014748.01;MsG0680034110.01;MsG0180006211.01;MsG0780041140.01;MsG0480022703.01;MsG0280010598.01;MsG0580025826.01;MsG0880043020.01;MsG0380016368.01;MsG0280010298.01;MsG0680034032.01;MsG0180005819.01;MsG0780039096.01;MsG0780041750.01;MsG0680031334.01;MsG0780040738.01;MsG0480020220.01;MsG0680034028.01;MsG0780039135.01;MsG0880043827.01;MsG0280010378.01;MsG0780039113.01;MsG0380015152.01;MsG0280009139.01;MsG0580026003.01;MsG0280010607.01;MsG0880041970.01;MsG0280006918.01;MsG0280009321.01;MsG0880046406.01;MsG0180005687.01;MsG0780038373.01;MsG0580027899.01;MsG0280010311.01;MsG0680030604.01;MsG0380014847.01;MsG0480023551.01;MsG0580028496.01;MsG0880042993.01;MsG0280011027.01;MsG0480023689.01;MsG0680032095.01;MsG0280010648.01;MsG0480022642.01;MsG0880047419.01;MsG0180000577.01;MsG0480022900.01;MsG0380015801.01;MsG0480022099.01;MsG0480023969.01;MsG0580027113.01;MsG0880043495.01;MsG0380017008.01;MsG0280008988.01;MsG0880046408.01;MsG0880042139.01;MsG0280007212.01;MsG0180005298.01;MsG0480018351.01;MsG0080049160.01;MsG0480023114.01;MsG0580028316.01;MsG0180000842.01;MsG0780040756.01;MsG0380014582.01;MsG0380015233.01;MsG0180000962.01;MsG0480023916.01;MsG0880041902.01;MsG0880043786.01;MsG0880043828.01;MsG0880046652.01;MsG0880045339.01;MsG0880045163.01;MsG0280008072.01;MsG0180000970.01;MsG0180003808.01;MsG0880043363.01;MsG0880041971.01;MsG0380017598.01;MsG0280006479.01;MsG0580024842.01;MsG0280007478.01;MsG0180004977.01;MsG0180005673.01;MsG0580027832.01;MsG0880043647.01;MsG0380012807.01;MsG0280008107.01;MsG0280007708.01 |
| GO:0044421 | extracellular region part | Cellular component | 8 | 8/ 878 | MsG0380013785.01;MsG0380013788.01;MsG0180005142.01;MsG0280006550.01;MsG0580027749.01;MsG0380017109.01;MsG0280009992.01;MsG0380013802.01 |
| GO:0044422 | organelle part | Cellular component | 102 | 102/ 878 | MsG0880042009.01;MsG0380014047.01;MsG0580028107.01;MsG0680033015.01;MsG0180002226.01;MsG0780040237.01;MsG0780041516.01;MsG0680031962.01;MsG0880045386.01;MsG0180002438.01;MsG0580029953.01;MsG0680034852.01;MsG0780038861.01;MsG0380017382.01;MsG0280009982.01;MsG0480019502.01;MsG0880046575.01;MsG0180004915.01;MsG0380016977.01;MsG0580025567.01;MsG0180003711.01;MsG0880043206.01;MsG0380014276.01;MsG0480022039.01;MsG0780039258.01;MsG0680035560.01;MsG0680031368.01;MsG0780036139.01;MsG0580027139.01;MsG0380013973.01;MsG0580026705.01;MsG0480021199.01;MsG0180000408.01;MsG0780041677.01;MsG0380013971.01;MsG0380016080.01;MsG0580028238.01;MsG0480023944.01;MsG0480023627.01;MsG0480023416.01;MsG0780041053.01;MsG0580024151.01;MsG0880047143.01;MsG0180006211.01;MsG0080048638.01;MsG0580029047.01;MsG0180004091.01;MsG0480023969.01;MsG0880041902.01;MsG0280008072.01;MsG0180004130.01;MsG0780040174.01;MsG0180003484.01;MsG0480023550.01;MsG0380017606.01;MsG0680035494.01;MsG0480022910.01;MsG0880043495.01;MsG0880045416.01;MsG0180004126.01;MsG0880046321.01;MsG0380013949.01;MsG0580027678.01;MsG0480020392.01;MsG0680035909.01;MsG0580028957.01;MsG0380011962.01;MsG0080048355.01;MsG0680032778.01;MsG0480021198.01;MsG0680035589.01;MsG0380012869.01;MsG0280010598.01;MsG0080048106.01;MsG0680032536.01;MsG0080048527.01;MsG0380013974.01;MsG0080048992.01;MsG0080047868.01;MsG0380017788.01;MsG0780040823.01;MsG0380014133.01;MsG0480018982.01;MsG0580028958.01;MsG0680030604.01;MsG0280007990.01;MsG0280011027.01;MsG0480023689.01;MsG0680030949.01;MsG0280007212.01;MsG0880044437.01;MsG0080049160.01;MsG0780040756.01;MsG0180003920.01;MsG0380015233.01;MsG0480022032.01;MsG0780037780.01;MsG0680034110.01;MsG0880046760.01;MsG0480022662.01;MsG0380014233.01;MsG0180003377.01 |
| GO:0043226 | organelle | Cellular component | 202 | 202/ 878 | MsG0180000284.01;MsG0380016941.01;MsG0880046991.01;MsG0680033015.01;MsG0180003243.01;MsG0180002226.01;MsG0480022664.01;MsG0780040237.01;MsG0280007781.01;MsG0880046412.01;MsG0180002793.01;MsG0880044444.01;MsG0480021976.01;MsG0680031962.01;MsG0880045386.01;MsG0880047578.01;MsG0880042896.01;MsG0480020134.01;MsG0280008601.01;MsG0580027749.01;MsG0180000205.01;MsG0580028196.01;MsG0180002438.01;MsG0580029953.01;MsG0180004915.01;MsG0480020827.01;MsG0380017382.01;MsG0680034475.01;MsG0880047250.01;MsG0280009992.01;MsG0180004623.01;MsG0780041078.01;MsG0180005142.01;MsG0480021267.01;MsG0880046036.01;MsG0680031206.01;MsG0580027523.01;MsG0280010916.01;MsG0480022746.01;MsG0880046575.01;MsG0480023401.01;MsG0180006211.01;MsG0180004830.01;MsG0880047061.01;MsG0180000075.01;MsG0580025567.01;MsG0180003711.01;MsG0880043206.01;MsG0580029737.01;MsG0380014276.01;MsG0580025833.01;MsG0580025664.01;MsG0180002138.01;MsG0380017793.01;MsG0180001484.01;MsG0880047143.01;MsG0480021655.01;MsG0780039258.01;MsG0480023248.01;MsG0680034495.01;MsG0880043916.01;MsG0480019502.01;MsG0180002458.01;MsG0680031368.01;MsG0280008879.01;MsG0680031278.01;MsG0380015148.01;MsG0780036139.01;MsG0780041062.01;MsG0280007699.01;MsG0180004917.01;MsG0380013973.01;MsG0580026705.01;MsG0780036173.01;MsG0280009812.01;MsG0480022322.01;MsG0480023944.01;MsG0780041677.01;MsG0580027088.01;MsG0280007911.01;MsG0180001019.01;MsG0480018099.01;MsG0280007842.01;MsG0880045321.01;MsG0380013971.01;MsG0480023102.01;MsG0580028238.01;MsG0180005308.01;MsG0280010728.01;MsG0380016948.01;MsG0380012788.01;MsG0180000395.01;MsG0480023416.01;MsG0480023627.01;MsG0680034868.01;MsG0280010899.01;MsG0780036759.01;MsG0280006932.01;MsG0280007906.01;MsG0280009072.01;MsG0680031284.01;MsG0680030918.01;MsG0880042986.01;MsG0480020534.01;MsG0380015819.01;MsG0080048638.01;MsG0880041902.01;MsG0180004130.01;MsG0480023026.01;MsG0380013478.01;MsG0180002924.01;MsG0780040174.01;MsG0580025514.01;MsG0580025659.01;MsG0780041648.01;MsG0680035353.01;MsG0680031105.01;MsG0480020492.01;MsG0380014422.01;MsG0380015497.01;MsG0480023550.01;MsG0180004103.01;MsG0180005718.01;MsG0680035514.01;MsG0480022910.01;MsG0680031280.01;MsG0580027261.01;MsG0880045416.01;MsG0580024151.01;MsG0880046321.01;MsG0580029315.01;MsG0880047081.01;MsG0380013949.01;MsG0080048527.01;MsG0680035909.01;MsG0580028957.01;MsG0380011962.01;MsG0280010901.01;MsG0480022099.01;MsG0380014133.01;MsG0680033830.01;MsG0180003906.01;MsG0680035589.01;MsG0580024974.01;MsG0380012869.01;MsG0480022895.01;MsG0080048106.01;MsG0280007870.01;MsG0380015519.01;MsG0480022724.01;MsG0880047288.01;MsG0380017155.01;MsG0380013974.01;MsG0480020220.01;MsG0380016962.01;MsG0380013547.01;MsG0680031277.01;MsG0280010593.01;MsG0380015152.01;MsG0380017788.01;MsG0780040823.01;MsG0580024855.01;MsG0180004622.01;MsG0280009321.01;MsG0280011162.01;MsG0280010311.01;MsG0580028958.01;MsG0680030604.01;MsG0880042581.01;MsG0880043343.01;MsG0480021587.01;MsG0180005005.01;MsG0680034435.01;MsG0680030342.01;MsG0280010856.01;MsG0480022848.01;MsG0580027113.01;MsG0480023251.01;MsG0680030949.01;MsG0280007212.01;MsG0680032778.01;MsG0580024328.01;MsG0180003920.01;MsG0480022599.01;MsG0880046652.01;MsG0580025906.01;MsG0880046208.01;MsG0480022032.01;MsG0480022512.01;MsG0880043363.01;MsG0680034110.01;MsG0080048202.01;MsG0880046760.01;MsG0780040676.01;MsG0680031403.01;MsG0580025612.01;MsG0480022662.01;MsG0580028107.01;MsG0180001799.01;MsG0380014233.01;MsG0180003377.01;MsG0680033821.01 |
| GO:0016020 | membrane | Cellular component | 124 | 124/ 878 | MsG0880047563.01;MsG0280006533.01;MsG0780040237.01;MsG0780041516.01;MsG0280011470.01;MsG0480023551.01;MsG0480023819.01;MsG0780036054.01;MsG0680034814.01;MsG0680034852.01;MsG0880044311.01;MsG0880046277.01;MsG0880044640.01;MsG0380014933.01;MsG0280010864.01;MsG0480022746.01;MsG0280008764.01;MsG0380016977.01;MsG0480020880.01;MsG0780038753.01;MsG0280011288.01;MsG0680030558.01;MsG0480022639.01;MsG0880045943.01;MsG0580025586.01;MsG0580024401.01;MsG0580028422.01;MsG0580025670.01;MsG0780039258.01;MsG0480020913.01;MsG0380017791.01;MsG0580029429.01;MsG0580025674.01;MsG0880047514.01;MsG0580025468.01;MsG0480022515.01;MsG0380014438.01;MsG0380013971.01;MsG0680035323.01;MsG0180005308.01;MsG0480023944.01;MsG0380014276.01;MsG0880042368.01;MsG0680034036.01;MsG0680034868.01;MsG0780041053.01;MsG0180002325.01;MsG0180000942.01;MsG0280010607.01;MsG0880044373.01;MsG0080048992.01;MsG0580027828.01;MsG0180004091.01;MsG0480023969.01;MsG0880041902.01;MsG0180000976.01;MsG0580024842.01;MsG0180005151.01;MsG0880046586.01;MsG0580027661.01;MsG0680032431.01;MsG0280009139.01;MsG0680034914.01;MsG0480023550.01;MsG0880043495.01;MsG0780040510.01;MsG0180006211.01;MsG0380015825.01;MsG0680032925.01;MsG0780038826.01;MsG0580029467.01;MsG0480023630.01;MsG0280008072.01;MsG0080048355.01;MsG0780040804.01;MsG0880046046.01;MsG0580026206.01;MsG0380014748.01;MsG0580026594.01;MsG0480022703.01;MsG0280010598.01;MsG0580025826.01;MsG0880043020.01;MsG0280010298.01;MsG0680034032.01;MsG0180005819.01;MsG0080048527.01;MsG0680034028.01;MsG0880043827.01;MsG0580026003.01;MsG0880041970.01;MsG0280006918.01;MsG0680032778.01;MsG0880046406.01;MsG0180005687.01;MsG0880045958.01;MsG0180000993.01;MsG0480019502.01;MsG0580028496.01;MsG0280011027.01;MsG0480022642.01;MsG0180000577.01;MsG0480022900.01;MsG0380015801.01;MsG0180000125.01;MsG0880044309.01;MsG0880046408.01;MsG0280007212.01;MsG0180005298.01;MsG0880044437.01;MsG0080049160.01;MsG0480023114.01;MsG0480020818.01;MsG0180000842.01;MsG0880043786.01;MsG0880045339.01;MsG0880043828.01;MsG0880041971.01;MsG0680034110.01;MsG0280007478.01;MsG0380012411.01;MsG0580027832.01;MsG0880043647.01;MsG0280008107.01 |
| GO:0030054 | cell junction | Cellular component | 2 | 2/ 878 | MsG0280010378.01;MsG0180004027.01 |
| GO:0005576 | extracellular region | cellular_component | 38 | 38/ 878 | MsG0180000271.01;MsG0580027615.01;MsG0880045544.01;MsG0180001480.01;MsG0280006973.01;MsG0380015858.01;MsG0780041533.01;MsG0780041696.01;MsG0380017683.01;MsG0380015544.01;MsG0180004741.01;MsG0780038613.01;MsG0780041688.01;MsG0780039923.01;MsG0680032617.01;MsG0280008352.01;MsG0680032686.01;MsG0280010428.01;MsG0280008349.01;MsG0880046140.01;MsG0380017684.01;MsG0680034255.01;MsG0280006550.01;MsG0280008350.01;MsG0280007684.01;MsG0580026206.01;MsG0280007677.01;MsG0780038753.01;MsG0680034980.01;MsG0380017394.01;MsG0480021761.01;MsG0380015498.01;MsG0880043093.01;MsG0680034317.01;MsG0580027605.01;MsG0480023682.01;MsG0280008357.01;MsG0580026176.01 |
| GO:0044217 | other organism part | Cellular component | 5 | 5/ 878 | MsG0580024855.01;MsG0580029315.01;MsG0680035353.01;MsG0680030342.01;MsG0580025586.01 |
| GO:0044464 | cell part | Cellular component | 367 | 367/ 878 | MsG0380016941.01;MsG0680033015.01;MsG0680031150.01;MsG0780041516.01;MsG0880045386.01;MsG0180003404.01;MsG0280008601.01;MsG0580028196.01;MsG0280009982.01;MsG0780041078.01;MsG0480022746.01;MsG0880046575.01;MsG0580025409.01;MsG0480020880.01;MsG0180004293.01;MsG0180000075.01;MsG0180003711.01;MsG0180004921.01;MsG0780039258.01;MsG0680031368.01;MsG0280007699.01;MsG0380016110.01;MsG0780036173.01;MsG0880046412.01;MsG0580025468.01;MsG0080049045.01;MsG0480023102.01;MsG0380016948.01;MsG0480023627.01;MsG0680034036.01;MsG0680034868.01;MsG0280007337.01;MsG0280010899.01;MsG0780036759.01;MsG0280006932.01;MsG0280007906.01;MsG0280009072.01;MsG0680030918.01;MsG0280007354.01;MsG0880046991.01;MsG0380013478.01;MsG0180005151.01;MsG0880047294.01;MsG0880046586.01;MsG0680035514.01;MsG0380017707.01;MsG0380017404.01;MsG0180004126.01;MsG0880047081.01;MsG0380013949.01;MsG0880047296.01;MsG0680032925.01;MsG0780038826.01;MsG0680035909.01;MsG0480022099.01;MsG0080048355.01;MsG0280009321.01;MsG0680033830.01;MsG0480021198.01;MsG0580024974.01;MsG0380012869.01;MsG0480022895.01;MsG0480022703.01;MsG0380017155.01;MsG0380016962.01;MsG0680031277.01;MsG0380015819.01;MsG0380015152.01;MsG0380017788.01;MsG0280010856.01;MsG0880043343.01;MsG0480021587.01;MsG0280007990.01;MsG0280011027.01;MsG0480023689.01;MsG0880042581.01;MsG0480022848.01;MsG0580027113.01;MsG0480023251.01;MsG0680030949.01;MsG0480023549.01;MsG0280007212.01;MsG0580028316.01;MsG0180000984.01;MsG0180003920.01;MsG0480022599.01;MsG0880042495.01;MsG0480022512.01;MsG0780040676.01;MsG0480022662.01;MsG0180001799.01;MsG0680033821.01;MsG0880047563.01;MsG0580028107.01;MsG0180003243.01;MsG0480022664.01;MsG0280006855.01;MsG0380016458.01;MsG0180002793.01;MsG0680031962.01;MsG0880047578.01;MsG0880042896.01;MsG0580027749.01;MsG0580027261.01;MsG0480021267.01;MsG0280010864.01;MsG0380015414.01;MsG0180003484.01;MsG0480022639.01;MsG0580025833.01;MsG0580029429.01;MsG0280008879.01;MsG0680031278.01;MsG0480022346.01;MsG0780036139.01;MsG0880047514.01;MsG0480022322.01;MsG0580027088.01;MsG0280007842.01;MsG0880045943.01;MsG0280010728.01;MsG0480023416.01;MsG0680032380.01;MsG0780041053.01;MsG0480022177.01;MsG0680031284.01;MsG0880044373.01;MsG0080048638.01;MsG0580028593.01;MsG0380017793.01;MsG0880041902.01;MsG0180002272.01;MsG0880044311.01;MsG0780040174.01;MsG0780038937.01;MsG0780041648.01;MsG0680031105.01;MsG0380014422.01;MsG0480023550.01;MsG0180004103.01;MsG0680031280.01;MsG0880046321.01;MsG0580029315.01;MsG0780041750.01;MsG0880046327.01;MsG0180003906.01;MsG0680035589.01;MsG0480018324.01;MsG0580030133.01;MsG0280010598.01;MsG0680032095.01;MsG0680032536.01;MsG0880047288.01;MsG0480020392.01;MsG0480020220.01;MsG0880043827.01;MsG0480022345.01;MsG0280006918.01;MsG0880046406.01;MsG0480018982.01;MsG0380013547.01;MsG0480018146.01;MsG0680030342.01;MsG0580030135.01;MsG0480022642.01;MsG0180000577.01;MsG0380015801.01;MsG0480023604.01;MsG0880046408.01;MsG0580024328.01;MsG0080047999.01;MsG0080049160.01;MsG0680031055.01;MsG0480023916.01;MsG0880043786.01;MsG0180000068.01;MsG0880046652.01;MsG0380012807.01;MsG0080048106.01;MsG0780041477.01;MsG0380017598.01;MsG0880046760.01;MsG0680031403.01;MsG0580025612.01;MsG0580025906.01;MsG0380014233.01;MsG0380014047.01;MsG0480021199.01;MsG0180002226.01;MsG0880047143.01;MsG0180000284.01;MsG0280007911.01;MsG0680034852.01;MsG0780038861.01;MsG0380017382.01;MsG0680034475.01;MsG0180004623.01;MsG0180005142.01;MsG0180001068.01;MsG0880042314.01;MsG0580027523.01;MsG0180004830.01;MsG0380016977.01;MsG0480020534.01;MsG0280011288.01;MsG0580025567.01;MsG0880043206.01;MsG0580029737.01;MsG0580025586.01;MsG0180002138.01;MsG0380015691.01;MsG0480022039.01;MsG0180001484.01;MsG0480021891.01;MsG0580025670.01;MsG0480023248.01;MsG0880043916.01;MsG0680035560.01;MsG0180002458.01;MsG0780041062.01;MsG0480022724.01;MsG0280009812.01;MsG0880044640.01;MsG0280009992.01;MsG0180001019.01;MsG0780036953.01;MsG0580028238.01;MsG0780036951.01;MsG0180000395.01;MsG0880042294.01;MsG0580029047.01;MsG0480020939.01;MsG0580027661.01;MsG0580025659.01;MsG0680035353.01;MsG0480020492.01;MsG0180005308.01;MsG0780040823.01;MsG0480021976.01;MsG0880045416.01;MsG0580024151.01;MsG0880042986.01;MsG0580027678.01;MsG0080048527.01;MsG0480022595.01;MsG0580029467.01;MsG0380011962.01;MsG0680035493.01;MsG0480023114.01;MsG0680032778.01;MsG0880046046.01;MsG0280010298.01;MsG0680034028.01;MsG0580028958.01;MsG0180004622.01;MsG0380014133.01;MsG0180005687.01;MsG0580024333.01;MsG0280011162.01;MsG0480019502.01;MsG0180005005.01;MsG0680034435.01;MsG0180005253.01;MsG0880044309.01;MsG0380018047.01;MsG0180005298.01;MsG0880044437.01;MsG0480020818.01;MsG0780040756.01;MsG0780037057.01;MsG0880043828.01;MsG0480022032.01;MsG0680034110.01;MsG0580027832.01;MsG0780037780.01;MsG0180003377.01;MsG0280010916.01;MsG0880042009.01;MsG0780040237.01;MsG0580030199.01;MsG0880044444.01;MsG0180004915.01;MsG0780041082.01;MsG0180000205.01;MsG0180002438.01;MsG0580029953.01;MsG0480020827.01;MsG0880044688.01;MsG0880046036.01;MsG0680031206.01;MsG0280007781.01;MsG0480023401.01;MsG0880047061.01;MsG0380014276.01;MsG0580025664.01;MsG0480021655.01;MsG0280010769.01;MsG0680034495.01;MsG0180004917.01;MsG0580027139.01;MsG0380013973.01;MsG0580026705.01;MsG0180000408.01;MsG0780041677.01;MsG0480018099.01;MsG0280008334.01;MsG0880045321.01;MsG0380013971.01;MsG0380016080.01;MsG0480023944.01;MsG0380012788.01;MsG0480018991.01;MsG0680034032.01;MsG0780036467.01;MsG0580027828.01;MsG0180004091.01;MsG0480023969.01;MsG0280008072.01;MsG0880042130.01;MsG0180004130.01;MsG0480023026.01;MsG0180002924.01;MsG0580025514.01;MsG0380015148.01;MsG0680030558.01;MsG0380017606.01;MsG0180005718.01;MsG0680035494.01;MsG0480022910.01;MsG0880043495.01;MsG0180006211.01;MsG0580028957.01;MsG0280010901.01;MsG0380016028.01;MsG0280010593.01;MsG0380014748.01;MsG0580026594.01;MsG0580025826.01;MsG0280007870.01;MsG0380015519.01;MsG0680033485.01;MsG0180005819.01;MsG0380013974.01;MsG0080048992.01;MsG0880047250.01;MsG0280010378.01;MsG0080047868.01;MsG0380015497.01;MsG0580026003.01;MsG0580024855.01;MsG0680031087.01;MsG0280010311.01;MsG0880045958.01;MsG0680030604.01;MsG0180000993.01;MsG0280008107.01;MsG0880047419.01;MsG0080048202.01;MsG0180001856.01;MsG0880042139.01;MsG0480020134.01;MsG0380015233.01;MsG0880045339.01;MsG0480020941.01;MsG0880046208.01;MsG0880043363.01;MsG0180000751.01 |
| GO:0099080 | supramolecular complex | Cellular component | 12 | 12/ 878 | MsG0280009982.01;MsG0280007990.01;MsG0480023689.01;MsG0680030949.01;MsG0880044437.01;MsG0680035494.01;MsG0580026705.01;MsG0480023944.01;MsG0680035909.01;MsG0680032536.01;MsG0180004126.01;MsG0780037780.01 |
| GO:0045182 | translation regulator activity | Molecular  function | 13 | 13/ 878 | MsG0180001484.01;MsG0480020392.01;MsG0880042338.01;MsG0580029519.01;MsG0880047578.01;MsG0180005253.01;MsG0180002793.01;MsG0480018099.01;MsG0180000068.01;MsG0380014422.01;MsG0880046499.01;MsG0480018324.01;MsG0480021587.01 |
| GO:0140110 | transcription regulator activity | Molecular  function | 36 | 36/ 878 | MsG0880043363.01;MsG0380015497.01;MsG0180002924.01;MsG0280009321.01;MsG0180000284.01;MsG0780041648.01;MsG0280008879.01;MsG0480022322.01;MsG0680034435.01;MsG0280010593.01;MsG0580026705.01;MsG0280008601.01;MsG0880046412.01;MsG0180001019.01;MsG0480023251.01;MsG0180004623.01;MsG0480023102.01;MsG0880047250.01;MsG0480022099.01;MsG0280006740.01;MsG0580024328.01;MsG0880043916.01;MsG0680032778.01;MsG0680033830.01;MsG0680034868.01;MsG0380015233.01;MsG0280006932.01;MsG0180004830.01;MsG0880046208.01;MsG0880043020.01;MsG0680034110.01;MsG0780040676.01;MsG0580025612.01;MsG0580025664.01;MsG0180002138.01;MsG0680033821.01 |
| GO:0005198 | structural molecule activity | Molecular  function | 24 | 24/ 878 | MsG0180004130.01;MsG0580028107.01;MsG0180004622.01;MsG0180002226.01;MsG0780040237.01;MsG0780041516.01;MsG0380013547.01;MsG0680031368.01;MsG0880044444.01;MsG0680031962.01;MsG0880042896.01;MsG0480022910.01;MsG0180002438.01;MsG0580027788.01;MsG0880047250.01;MsG0380017382.01;MsG0380013949.01;MsG0380016028.01;MsG0180003920.01;MsG0280007781.01;MsG0580024151.01;MsG0680032019.01;MsG0080048638.01;MsG0180003711.01 |
| GO:0044183 | protein folding chaperone | Molecular  function | 6 | 6/ 878 | MsG0780036953.01;MsG0480020941.01;MsG0780036951.01;MsG0480020939.01;MsG0480022599.01;MsG0280008334.01 |
| GO:0140104 | molecular carrier activity | Molecular  function | 1 | 1/ 878 | MsG0880046991.01 |
| GO:0016209 | antioxidant activity | Molecular  function | 5 | 5/ 878 | MsG0280010428.01;MsG0380017883.01;MsG0280007677.01;MsG0280007684.01;MsG0380015544.01 |
| GO:0005215 | transporter activity | Molecular  function | 56 | 56/ 878 | MsG0580027803.01;MsG0180005151.01;MsG0880046586.01;MsG0480020913.01;MsG0880046406.01;MsG0480021200.01;MsG0580027899.01;MsG0280011470.01;MsG0680030746.01;MsG0680031105.01;MsG0680034914.01;MsG0180000993.01;MsG0480023819.01;MsG0180002325.01;MsG0180005308.01;MsG0580025674.01;MsG0680035323.01;MsG0480021199.01;MsG0480022900.01;MsG0380015801.01;MsG0580029282.01;MsG0880043495.01;MsG0580025468.01;MsG0480022515.01;MsG0780040510.01;MsG0880043598.01;MsG0380015825.01;MsG0880046408.01;MsG0480023630.01;MsG0280007212.01;MsG0180005298.01;MsG0280010864.01;MsG0080049160.01;MsG0180000842.01;MsG0280010607.01;MsG0480021198.01;MsG0880043786.01;MsG0280011027.01;MsG0680030677.01;MsG0880044309.01;MsG0280011288.01;MsG0480022703.01;MsG0780040738.01;MsG0580025826.01;MsG0880043020.01;MsG0880044311.01;MsG0680034814.01;MsG0280007478.01;MsG0380018054.01;MsG0180000774.01;MsG0580025586.01;MsG0380012411.01;MsG0480023969.01;MsG0880043647.01;MsG0380012807.01;MsG0780041677.01 |
| GO:0098772 | molecular function regulator | Molecular  function | 20 | 20/ 878 | MsG0680030949.01;MsG0380013788.01;MsG0880046140.01;MsG0380013785.01;MsG0680035493.01;MsG0680034255.01;MsG0680034317.01;MsG0580029583.01;MsG0680034980.01;MsG0380017394.01;MsG0180004110.01;MsG0380017109.01;MsG0280007911.01;MsG0380014276.01;MsG0380013802.01;MsG0880047081.01;MsG0880047187.01;MsG0680032617.01;MsG0280007906.01;MsG0680032686.01 |
| GO:0140299 | small molecule sensor activity | Molecular  function | 1 | 1/ 878 | MsG0680035909.01 |
| GO:0005488 | binding | Molecular  function | 492 | 492/ 878 | MsG0780039591.01;MsG0380016941.01;MsG0680033015.01;MsG0380012211.01;MsG0380015326.01;MsG0780041516.01;MsG0880045608.01;MsG0480020083.01;MsG0180004978.01;MsG0680031814.01;MsG0580029491.01;MsG0880045386.01;MsG0280008601.01;MsG0780037939.01;MsG0480022111.01;MsG0680031150.01;MsG0280009982.01;MsG0780041078.01;MsG0280009582.01;MsG0780039503.01;MsG0880046575.01;MsG0580025409.01;MsG0480020880.01;MsG0880042478.01;MsG0180000075.01;MsG0180003711.01;MsG0680032238.01;MsG0280008334.01;MsG0180000936.01;MsG0580024401.01;MsG0580024551.01;MsG0480019538.01;MsG0480021489.01;MsG0180004921.01;MsG0280009717.01;MsG0780039258.01;MsG0680031997.01;MsG0480021199.01;MsG0780036173.01;MsG0880046412.01;MsG0880045573.01;MsG0780036333.01;MsG0280008678.01;MsG0680035102.01;MsG0180001325.01;MsG0480023102.01;MsG0380017684.01;MsG0380016948.01;MsG0480023627.01;MsG0880046518.01;MsG0680034036.01;MsG0280007684.01;MsG0680034868.01;MsG0280010899.01;MsG0280006932.01;MsG0280007906.01;MsG0580028455.01;MsG0680030918.01;MsG0680035017.01;MsG0280007124.01;MsG0280007354.01;MsG0880046991.01;MsG0380013478.01;MsG0180005151.01;MsG0880046586.01;MsG0780041255.01;MsG0280009541.01;MsG0480021200.01;MsG0380011895.01;MsG0280006415.01;MsG0680035514.01;MsG0880047165.01;MsG0180004126.01;MsG0880047081.01;MsG0380013949.01;MsG0680032925.01;MsG0780038826.01;MsG0680035909.01;MsG0280007878.01;MsG0480022099.01;MsG0080048355.01;MsG0680031241.01;MsG0680033830.01;MsG0480021198.01;MsG0580024974.01;MsG0380012869.01;MsG0480022895.01;MsG0880043020.01;MsG0580027139.01;MsG0680034032.01;MsG0180004692.01;MsG0380012553.01;MsG0480020547.01;MsG0480021600.01;MsG0780039113.01;MsG0380017788.01;MsG0480020344.01;MsG0480019829.01;MsG0180002362.01;MsG0880042581.01;MsG0380012264.01;MsG0480021587.01;MsG0280007990.01;MsG0880042993.01;MsG0280011027.01;MsG0480023689.01;MsG0380017683.01;MsG0680031254.01;MsG0480022848.01;MsG0580027113.01;MsG0480023251.01;MsG0280008988.01;MsG0680030949.01;MsG0480018351.01;MsG0680034723.01;MsG0180003920.01;MsG0180000962.01;MsG0480022599.01;MsG0680030677.01;MsG0180000976.01;MsG0880042495.01;MsG0480022512.01;MsG0280008072.01;MsG0780040676.01;MsG0280010648.01;MsG0480022662.01;MsG0180001799.01;MsG0680033821.01;MsG0880047563.01;MsG0380013796.01;MsG0180003243.01;MsG0480022664.01;MsG0280006855.01;MsG0680032833.01;MsG0380015858.01;MsG0380016458.01;MsG0480019426.01;MsG0180002793.01;MsG0180003753.01;MsG0180002924.01;MsG0880047578.01;MsG0780036054.01;MsG0180004104.01;MsG0580027261.01;MsG0880046277.01;MsG0880043598.01;MsG0180000557.01;MsG0480021267.01;MsG0480021529.01;MsG0780037982.01;MsG0780038376.01;MsG0380015414.01;MsG0380013761.01;MsG0480019359.01;MsG0380018047.01;MsG0680034998.01;MsG0580029429.01;MsG0280008879.01;MsG0480022346.01;MsG0580025584.01;MsG0880047514.01;MsG0480022322.01;MsG0580027088.01;MsG0280007842.01;MsG0380014438.01;MsG0280010728.01;MsG0580028614.01;MsG0380015328.01;MsG0680031284.01;MsG0280008720.01;MsG0880044373.01;MsG0580028593.01;MsG0880041902.01;MsG0580028702.01;MsG0680035666.01;MsG0180002272.01;MsG0380011483.01;MsG0780040174.01;MsG0580030137.01;MsG0580028422.01;MsG0680030746.01;MsG0680031105.01;MsG0380012566.01;MsG0380014422.01;MsG0480023550.01;MsG0180004103.01;MsG0480023911.01;MsG0180002030.01;MsG0580029315.01;MsG0780041750.01;MsG0680030529.01;MsG0880045572.01;MsG0780039580.01;MsG0680030892.01;MsG0780040804.01;MsG0180003906.01;MsG0680035589.01;MsG0480018324.01;MsG0580030133.01;MsG0880042338.01;MsG0680032095.01;MsG0680032536.01;MsG0780039096.01;MsG0480020392.01;MsG0480020220.01;MsG0780039135.01;MsG0880043827.01;MsG0180000472.01;MsG0280006918.01;MsG0780038373.01;MsG0380012272.01;MsG0780039998.01;MsG0880047269.01;MsG0680030342.01;MsG0580030135.01;MsG0480018352.01;MsG0180000577.01;MsG0480021599.01;MsG0480023604.01;MsG0380013856.01;MsG0080048035.01;MsG0580024328.01;MsG0080049160.01;MsG0680031055.01;MsG0880046327.01;MsG0380017707.01;MsG0180000068.01;MsG0580024168.01;MsG0880045163.01;MsG0080048106.01;MsG0580029697.01;MsG0180000774.01;MsG0680031403.01;MsG0580025612.01;MsG0580025906.01;MsG0380014233.01;MsG0380014047.01;MsG0180002226.01;MsG0880047143.01;MsG0180000284.01;MsG0580028049.01;MsG0880047093.01;MsG0580024467.01;MsG0280007911.01;MsG0480021692.01;MsG0380017189.01;MsG0780038613.01;MsG0080048805.01;MsG0380015135.01;MsG0680034475.01;MsG0180004623.01;MsG0280006533.01;MsG0880047250.01;MsG0580026594.01;MsG0180004830.01;MsG0080048322.01;MsG0480020534.01;MsG0780039499.01;MsG0380011921.01;MsG0580025567.01;MsG0780041221.01;MsG0880043206.01;MsG0880042714.01;MsG0580029737.01;MsG0580025586.01;MsG0180002138.01;MsG0380015691.01;MsG0480022039.01;MsG0180001484.01;MsG0680035118.01;MsG0480021891.01;MsG0480023248.01;MsG0880043916.01;MsG0680035560.01;MsG0180002458.01;MsG0780041062.01;MsG0880042613.01;MsG0880047697.01;MsG0280009812.01;MsG0880044640.01;MsG0880046043.01;MsG0180001019.01;MsG0380012197.01;MsG0680030359.01;MsG0780036953.01;MsG0280006740.01;MsG0780036951.01;MsG0780040823.01;MsG0880042368.01;MsG0580025261.01;MsG0180002830.01;MsG0480020896.01;MsG0680030953.01;MsG0580027470.01;MsG0480020366.01;MsG0480020939.01;MsG0380015267.01;MsG0380012194.01;MsG0580027661.01;MsG0580025659.01;MsG0680035353.01;MsG0680034225.01;MsG0480020492.01;MsG0580025683.01;MsG0880042246.01;MsG0880045416.01;MsG0880042986.01;MsG0580027678.01;MsG0080048527.01;MsG0780038914.01;MsG0580029467.01;MsG0480023630.01;MsG0380011962.01;MsG0680035493.01;MsG0880042998.01;MsG0480022209.01;MsG0880043554.01;MsG0880046046.01;MsG0680034510.01;MsG0480022342.01;MsG0280010298.01;MsG0680034028.01;MsG0780036879.01;MsG0480023761.01;MsG0180004622.01;MsG0380014133.01;MsG0180005687.01;MsG0580024333.01;MsG0280011162.01;MsG0880045122.01;MsG0380016334.01;MsG0880046050.01;MsG0880047171.01;MsG0680034435.01;MsG0780037877.01;MsG0180005253.01;MsG0580027221.01;MsG0480023682.01;MsG0280010428.01;MsG0180005298.01;MsG0880044437.01;MsG0880043094.01;MsG0780040756.01;MsG0580025352.01;MsG0880043828.01;MsG0480022032.01;MsG0280010802.01;MsG0880041971.01;MsG0680034110.01;MsG0580027832.01;MsG0780037780.01;MsG0180003377.01;MsG0280007708.01;MsG0280010916.01;MsG0880042009.01;MsG0580027513.01;MsG0880043930.01;MsG0780040237.01;MsG0380015268.01;MsG0380017926.01;MsG0880044083.01;MsG0880044444.01;MsG0380017155.01;MsG0180004915.01;MsG0380015544.01;MsG0180003854.01;MsG0780035923.01;MsG0180000205.01;MsG0780039388.01;MsG0180002438.01;MsG0480020827.01;MsG0880043550.01;MsG0880043483.01;MsG0080047999.01;MsG0880046036.01;MsG0080047910.01;MsG0780040101.01;MsG0280008674.01;MsG0480022345.01;MsG0580024487.01;MsG0280007781.01;MsG0480023401.01;MsG0880047061.01;MsG0380017913.01;MsG0380014276.01;MsG0580025664.01;MsG0080049111.01;MsG0680032431.01;MsG0480021655.01;MsG0280010769.01;MsG0880042001.01;MsG0380011649.01;MsG0280007677.01;MsG0880041970.01;MsG0680034797.01;MsG0180004917.01;MsG0280011054.01;MsG0380013973.01;MsG0580026705.01;MsG0380016341.01;MsG0180000970.01;MsG0480018099.01;MsG0280010248.01;MsG0880043852.01;MsG0880045321.01;MsG0380013971.01;MsG0480023944.01;MsG0380012788.01;MsG0480018991.01;MsG0380018025.01;MsG0780039894.01;MsG0180000942.01;MsG0380015819.01;MsG0680034499.01;MsG0580027828.01;MsG0180004091.01;MsG0580029519.01;MsG0280007481.01;MsG0780035943.01;MsG0680033003.01;MsG0180004130.01;MsG0480023026.01;MsG0380014410.01;MsG0580025880.01;MsG0780037607.01;MsG0580025514.01;MsG0180003993.01;MsG0180003484.01;MsG0680030558.01;MsG0280008257.01;MsG0880044380.01;MsG0380017606.01;MsG0180005718.01;MsG0580028710.01;MsG0680035494.01;MsG0380012035.01;MsG0180006081.01;MsG0380017993.01;MsG0780038647.01;MsG0780039585.01;MsG0280010901.01;MsG0680031996.01;MsG0280008597.01;MsG0280006919.01;MsG0280010593.01;MsG0380014748.01;MsG0180000271.01;MsG0680031318.01;MsG0680031294.01;MsG0880046208.01;MsG0680032245.01;MsG0280007870.01;MsG0480018187.01;MsG0380015519.01;MsG0680033485.01;MsG0180005819.01;MsG0280009321.01;MsG0680030429.01;MsG0080047868.01;MsG0380015497.01;MsG0580026003.01;MsG0580024855.01;MsG0680032778.01;MsG0280010311.01;MsG0180004385.01;MsG0180005005.01;MsG0580028107.01;MsG0380014847.01;MsG0280007427.01;MsG0280008107.01;MsG0780036197.01;MsG0080048202.01;MsG0880046760.01;MsG0780036882.01;MsG0180001856.01;MsG0180000125.01;MsG0880046499.01;MsG0380012807.01;MsG0080048716.01;MsG0880046140.01;MsG0480020134.01;MsG0480023742.01;MsG0380015233.01;MsG0680030976.01;MsG0280009072.01;MsG0480020941.01;MsG0780038937.01;MsG0880043363.01;MsG0580028496.01;MsG0180000751.01;MsG0280008756.01;MsG0580026176.01 |
| GO:0045735 | nutrient reservoir activity | Molecular  function | 1 | 1/ 878 | MsG0180002490.01 |
| GO:0060089 | molecular transducer activity | Molecular  function | 15 | 15/ 878 | MsG0480020880.01;MsG0680035666.01;MsG0880047514.01;MsG0580029467.01;MsG0280007911.01;MsG0880047165.01;MsG0680030558.01;MsG0280007906.01;MsG0880046046.01;MsG0580029429.01;MsG0880047081.01;MsG0580026594.01;MsG0480023401.01;MsG0380014748.01;MsG0780038826.01 |
| GO:0003824 | catalytic activity | Molecular  function | 450 | 450/ 878 | MsG0680030916.01;MsG0780039591.01;MsG0380016941.01;MsG0680033015.01;MsG0380012211.01;MsG0380015326.01;MsG0480020083.01;MsG0180004978.01;MsG0580029491.01;MsG0880046461.01;MsG0180003404.01;MsG0580028196.01;MsG0780037939.01;MsG0480022111.01;MsG0780041078.01;MsG0280009582.01;MsG0680034510.01;MsG0780040980.01;MsG0680034495.01;MsG0480022746.01;MsG0180003753.01;MsG0580025409.01;MsG0280007677.01;MsG0480020880.01;MsG0880042478.01;MsG0680032238.01;MsG0280008334.01;MsG0180000936.01;MsG0580024551.01;MsG0280008357.01;MsG0480019538.01;MsG0580027615.01;MsG0680031997.01;MsG0380016853.01;MsG0280006973.01;MsG0280007699.01;MsG0380016110.01;MsG0780036173.01;MsG0580025468.01;MsG0880045573.01;MsG0080049045.01;MsG0280008678.01;MsG0380017684.01;MsG0380016948.01;MsG0480023627.01;MsG0880046518.01;MsG0680034036.01;MsG0280007684.01;MsG0680034868.01;MsG0280007337.01;MsG0280010899.01;MsG0780036759.01;MsG0580028455.01;MsG0680030918.01;MsG0680034576.01;MsG0280008400.01;MsG0380017883.01;MsG0280007354.01;MsG0680034040.01;MsG0380013478.01;MsG0180005151.01;MsG0880047294.01;MsG0380015739.01;MsG0880046586.01;MsG0780041255.01;MsG0280009541.01;MsG0380011895.01;MsG0780039113.01;MsG0780041696.01;MsG0780036206.01;MsG0180000147.01;MsG0680035514.01;MsG0880047165.01;MsG0780039923.01;MsG0180004126.01;MsG0880047296.01;MsG0680032925.01;MsG0780038826.01;MsG0680035909.01;MsG0280007878.01;MsG0280010702.01;MsG0280006550.01;MsG0080048355.01;MsG0580024974.01;MsG0380012869.01;MsG0480022895.01;MsG0380016368.01;MsG0180000942.01;MsG0180004692.01;MsG0380012553.01;MsG0380016962.01;MsG0480021600.01;MsG0780038937.01;MsG0480020344.01;MsG0480019829.01;MsG0780038115.01;MsG0380017683.01;MsG0880043343.01;MsG0480021587.01;MsG0480023689.01;MsG0280010648.01;MsG0480022848.01;MsG0580027113.01;MsG0280008988.01;MsG0480023549.01;MsG0680034723.01;MsG0280006725.01;MsG0580028316.01;MsG0180000984.01;MsG0380017699.01;MsG0180000962.01;MsG0480022599.01;MsG0180003808.01;MsG0680030677.01;MsG0180000976.01;MsG0880042495.01;MsG0780036880.01;MsG0280008072.01;MsG0780040676.01;MsG0180001799.01;MsG0480018484.01;MsG0480022664.01;MsG0280006855.01;MsG0680032833.01;MsG0280009139.01;MsG0380015858.01;MsG0380016458.01;MsG0480019426.01;MsG0180002793.01;MsG0280008643.01;MsG0680031952.01;MsG0880042896.01;MsG0780036054.01;MsG0180004104.01;MsG0180002931.01;MsG0580027261.01;MsG0880046277.01;MsG0680031966.01;MsG0480021267.01;MsG0880047531.01;MsG0380015414.01;MsG0880041983.01;MsG0380013761.01;MsG0780039793.01;MsG0580025833.01;MsG0880047143.01;MsG0480022053.01;MsG0780039096.01;MsG0280010428.01;MsG0180001480.01;MsG0580024656.01;MsG0580029429.01;MsG0880047514.01;MsG0880044437.01;MsG0380014438.01;MsG0680034506.01;MsG0680035323.01;MsG0280010728.01;MsG0280006942.01;MsG0480023416.01;MsG0180002936.01;MsG0880045651.01;MsG0580028614.01;MsG0680032380.01;MsG0780041053.01;MsG0380015328.01;MsG0480022177.01;MsG0680031284.01;MsG0880044373.01;MsG0580028593.01;MsG0380017793.01;MsG0880041902.01;MsG0580028702.01;MsG0680035666.01;MsG0180002272.01;MsG0380011483.01;MsG0380015819.01;MsG0580030137.01;MsG0580028422.01;MsG0680030746.01;MsG0680031105.01;MsG0380014422.01;MsG0380015944.01;MsG0480023550.01;MsG0680031280.01;MsG0580027449.01;MsG0180002030.01;MsG0580029315.01;MsG0780041750.01;MsG0680030529.01;MsG0880045572.01;MsG0880046126.01;MsG0780039580.01;MsG0680030892.01;MsG0380015722.01;MsG0780040804.01;MsG0380015135.01;MsG0580030133.01;MsG0880042338.01;MsG0280010598.01;MsG0180005005.01;MsG0680032095.01;MsG0680032536.01;MsG0380015498.01;MsG0480020220.01;MsG0780039135.01;MsG0880043827.01;MsG0880042246.01;MsG0280006918.01;MsG0480018982.01;MsG0680030961.01;MsG0380012272.01;MsG0680030342.01;MsG0580030135.01;MsG0180000577.01;MsG0480021599.01;MsG0480023604.01;MsG0080047999.01;MsG0680031055.01;MsG0880046327.01;MsG0380017707.01;MsG0180000068.01;MsG0580024168.01;MsG0880045163.01;MsG0180005642.01;MsG0580029697.01;MsG0780041477.01;MsG0580025906.01;MsG0380014047.01;MsG0380017247.01;MsG0680034507.01;MsG0580024467.01;MsG0880047563.01;MsG0480021692.01;MsG0680034513.01;MsG0380017189.01;MsG0280006415.01;MsG0080048805.01;MsG0680034852.01;MsG0680034475.01;MsG0180004623.01;MsG0180005142.01;MsG0180001068.01;MsG0880042314.01;MsG0280010244.01;MsG0580027523.01;MsG0080048322.01;MsG0480020534.01;MsG0780039499.01;MsG0580025567.01;MsG0780041221.01;MsG0880043206.01;MsG0780037513.01;MsG0680030556.01;MsG0880042714.01;MsG0580029737.01;MsG0580025586.01;MsG0380015691.01;MsG0480022039.01;MsG0480021891.01;MsG0880043916.01;MsG0780041062.01;MsG0480023229.01;MsG0880042613.01;MsG0880047697.01;MsG0880044640.01;MsG0280009992.01;MsG0780038130.01;MsG0380012197.01;MsG0780036953.01;MsG0780036951.01;MsG0180000395.01;MsG0580024522.01;MsG0880042368.01;MsG0480023238.01;MsG0880042294.01;MsG0280009919.01;MsG0780037188.01;MsG0680030953.01;MsG0580027470.01;MsG0380017441.01;MsG0480020366.01;MsG0580027605.01;MsG0480020939.01;MsG0580025261.01;MsG0380012194.01;MsG0580027749.01;MsG0680034225.01;MsG0580025316.01;MsG0480020492.01;MsG0580026026.01;MsG0680033932.01;MsG0780039993.01;MsG0480023630.01;MsG0780037982.01;MsG0580027678.01;MsG0280008352.01;MsG0480022595.01;MsG0580029467.01;MsG0280008349.01;MsG0380011962.01;MsG0880042998.01;MsG0280006919.01;MsG0880043093.01;MsG0280008350.01;MsG0880046046.01;MsG0880043598.01;MsG0580024217.01;MsG0280010298.01;MsG0680034028.01;MsG0780036879.01;MsG0280007827.01;MsG0280009321.01;MsG0180005687.01;MsG0580024333.01;MsG0280011162.01;MsG0880045122.01;MsG0880043195.01;MsG0880041996.01;MsG0180005253.01;MsG0580026702.01;MsG0580027221.01;MsG0680034516.01;MsG0480023682.01;MsG0380018047.01;MsG0180005298.01;MsG0680030559.01;MsG0880043094.01;MsG0780037057.01;MsG0880043828.01;MsG0480022032.01;MsG0780040738.01;MsG0280007923.01;MsG0880041971.01;MsG0780037780.01;MsG0280007708.01;MsG0780036197.01;MsG0680034515.01;MsG0280006533.01;MsG0880043930.01;MsG0780038116.01;MsG0580030199.01;MsG0880044083.01;MsG0380015544.01;MsG0180003854.01;MsG0780041082.01;MsG0780039388.01;MsG0780040408.01;MsG0480020827.01;MsG0880043483.01;MsG0880044688.01;MsG0480019502.01;MsG0180000970.01;MsG0380012513.01;MsG0480023401.01;MsG0880047061.01;MsG0380017913.01;MsG0180004741.01;MsG0680035353.01;MsG0680032431.01;MsG0480021655.01;MsG0180003992.01;MsG0280010769.01;MsG0880042001.01;MsG0880042139.01;MsG0880041970.01;MsG0680034797.01;MsG0280011054.01;MsG0380013973.01;MsG0580026705.01;MsG0180000408.01;MsG0480018099.01;MsG0880043852.01;MsG0380013971.01;MsG0380016080.01;MsG0780041022.01;MsG0480023944.01;MsG0680034376.01;MsG0380012788.01;MsG0480018991.01;MsG0380018054.01;MsG0480023742.01;MsG0380018025.01;MsG0780039894.01;MsG0280008676.01;MsG0680034032.01;MsG0780036467.01;MsG0680034499.01;MsG0580029519.01;MsG0680031428.01;MsG0880042130.01;MsG0380013796.01;MsG0480023026.01;MsG0380014410.01;MsG0780041688.01;MsG0180000557.01;MsG0280008764.01;MsG0580025514.01;MsG0180003993.01;MsG0180003484.01;MsG0680030558.01;MsG0680032201.01;MsG0180005718.01;MsG0580028710.01;MsG0380012035.01;MsG0380017404.01;MsG0180006081.01;MsG0180006211.01;MsG0380017993.01;MsG0780038647.01;MsG0380015187.01;MsG0680031996.01;MsG0280008597.01;MsG0380016028.01;MsG0180000271.01;MsG0480020259.01;MsG0380014748.01;MsG0580026594.01;MsG0680032245.01;MsG0180000437.01;MsG0480018187.01;MsG0380015519.01;MsG0680033485.01;MsG0180005819.01;MsG0380013974.01;MsG0880047250.01;MsG0580026003.01;MsG0580024855.01;MsG0680031087.01;MsG0280010311.01;MsG0180004385.01;MsG0680030604.01;MsG0380014847.01;MsG0780036611.01;MsG0280007427.01;MsG0280008107.01;MsG0880047419.01;MsG0080048202.01;MsG0480022099.01;MsG0180001856.01;MsG0180000125.01;MsG0880046140.01;MsG0680031545.01;MsG0280009072.01;MsG0480020941.01;MsG0480018496.01;MsG0580028496.01;MsG0180000751.01;MsG0280008756.01;MsG0580026176.01 |
