# Supplementary material for: Physiological characteristics and transcriptomic analyses of alfalfa root crown in wintering
Source: Front Plant Sci. 2024 Dec 9;15:1486564. doi: 10.3389/fpls.2024.1486564 (PMC11663680; doi:10.3389/fpls.2024.1486564)
Supplement: Supplementary file 3 [file Table3.docx]

(TABLE S3) KEGG Classification Statistics Table for Gene Sets

| **First Category** | **Second Category** | **Description** | **Gene numbers** | **Gene_ids** |
| --- | --- | --- | --- | --- |
| Metabolism | Amino acid metabolism | Lysine degradation | 2 | MsG0880047296.01,  MsG0180001068.01 |
|  |  | Alanine, aspartate and glutamate metabolism | 3 | MsG0080048202.01,  MsG0080047999.01,  MsG0380015691.01 |
|  |  | Tryptophan metabolism | 2 | MsG0780041255.01,  MsG0180001068.01 |
|  |  | Valine, leucine and isoleucine biosynthesis | 1 | MsG0380017247.01 |
|  |  | Valine, leucine and isoleucine degradation | 5 | MsG0480018496.01,  MsG0380017247.01,  MsG0480018982.01,  MsG0180001068.01,  MsG0480020827.01 |
|  |  | Cysteine and methionine metabolism | 6 | MsG0880047697.01,  MsG0280007427.01,  MsG0180004692.01,  MsG0380017247.01,  MsG0880046461.01,  MsG0280009072.01 |
|  |  | Phenylalanine metabolism | 1 | MsG0580029697.01 |
|  |  | Tyrosine metabolism | 2 | MsG0380016458.01,  MsG0580029697.01 |
|  |  | Arginine biosynthesis | 1 | MsG0380015691.01 |
|  |  | Histidine metabolism | 1 | MsG0180001068.01 |
|  |  | Arginine and proline metabolism | 2 | MsG0480022895.01,MsG0180001068.01 |
| Metabolism | Lipid metabolism | Fatty acid biosynthesis | 1 | MsG0480018496.01 |
|  |  | Fatty acid degradation | 3 | MsG0380016458.01,  MsG0180001068.01,  MsG0880047061.01 |
|  |  | Fatty acid elongation | 1 | MsG0280009139.01 |
|  |  | Ether lipid metabolism | 1 | MsG0380016962.01 |
|  |  | Glycerophospholipid metabolism | 7 | MsG0480022177.01,  MsG0680034576.01,  MsG0380016962.01,  MsG0880046518.01,  MsG0380017441.01,  MsG0280006918.01,  MsG0680032380.01 |
|  |  | Glycerolipid metabolism | 5 | MsG0680034576.01,  MsG0880046518.01,  MsG0880047419.01,  MsG0780036467.01,  MsG0180001068.01 |
|  |  | Sphingolipid metabolism | 3 | MsG0580027749.01,  MsG0880047419.01,  MsG0780036467.01 |
|  |  | Steroid biosynthesis | 1 | MsG0780037188.01 |
|  |  | Arachidonic acid metabolism | 2 | MsG0680034475.01,  MsG0580027113.01 |
|  |  | alpha-Linolenic acid metabolism | 6 | MsG0880047061.01,  MsG0580024168.01,  MsG0380016458.01,  MsG0580027261.01,  MsG0780036759.01,  MsG0480022177.01 |
| Metabolism | Metabolism of cofactors and vitamins | Folate biosynthesis | 1 | MsG0280009992.01 |
|  |  | Porphyrin metabolism | 3 | MsG0180006211.01,  MsG0580030199.01,  MsG0380012513.01 |
|  |  | Pantothenate and CoA biosynthesis | 2 | MsG0180001068.01,  MsG0380017247.01 |
|  |  | Vitamin B6 metabolism | 2 | MsG0580024656.01,  MsG0280006942.01 |
|  |  | Ubiquinone and other terpenoid-quinone biosynthesis | 3 | MsG0680031284.01,  MsG0680031280.01,  MsG0580029697.01 |
| Metabolism | Metabolism of other amino acids | beta-Alanine metabolism | 1 | MsG0180001068.01 |
|  |  | Selenocompound metabolism | 1 | MsG0480018991.01 |
|  |  | Taurine and hypotaurine metabolism | 1 | MsG0280008988.01 |
|  |  | Glutathione metabolism | 4 | MsG0780036879.01,MsG0880041970.01,  MsG0180002272.01,MsG0780038937.01 |
| Metabolism | Carbohydrate metabolism | Butanoate metabolism | 1 | MsG0280011162.01 |
|  |  | Pentose and glucuronate interconversions | 3 | MsG0380017707.01,  MsG0880041983.01,  MsG0780038647.01 |
|  |  | Inositol phosphate metabolism | 4 | MsG0380016962.01,  MsG0880046327.01,  MsG0480019502.01,  MsG0880042495.01 |
|  |  | Fructose and mannose metabolism | 5 | MsG0080049045.01,  MsG0180003404.01,  MsG0780041082.01,  MsG0680033932.01,  MsG0180003808.01 |
|  |  | Ascorbate and aldarate metabolism | 3 | MsG0880042495.01,  MsG0180001068.01,  MsG0380015858.01 |
|  |  | Galactose metabolism | 8 | MsG0180000437.01,  MsG0780041477.01,  MsG0380015414.01,  MsG0580024333.01,  MsG0480023604.01,  MsG0280010769.01,  MsG0880047419.01,  MsG0780036467.01 |
|  |  | Citrate cycle (TCA cycle) | 1 | MsG0880046461.01 |
|  |  | Pentose phosphate pathway | 7 | MsG0080049045.01,MsG0180003404.01,  MsG0780041082.01,MsG0480023549.01,  MsG0780036879.01,MsG0880041970.01,  MsG0180000984.01 |
|  |  | Pyruvate metabolism | 4 | MsG0380016458.01,MsG0780041062.01,  MsG0180001068.01,MsG0880046461.01 |
|  |  | Starch and sucrose metabolism | 14 | MsG0480021267.01,MsG0780036173.01,  MsG0480020259.01,MsG0880045651.01,  MsG0880043093.01,MsG0480023682.01,  MsG0880047531.01,MsG0380016110.01,  MsG0180000271.01,MsG0580025567.01,  MsG0680030918.01,MsG0680033932.01,  MsG0180003808.01,MsG0180000437.01 |
|  |  | Glycolysis / Gluconeogenesis | 9 | MsG0380016458.01,MsG0080049045.01,  MsG0180003404.01,MsG0780041082.01,  MsG0780041062.01,MsG0680032095.01,  MsG0280009541.01,MsG0180001068.01,  MsG0280006855.01 |
|  |  | Propanoate metabolism | 1 | MsG0480018982.01 |
|  |  | Glyoxylate and dicarboxylate metabolism | 2 | MsG0280011162.01,MsG0880046461.01 |
|  |  | Amino sugar and nucleotide sugar metabolism | 24 | MsG0480021267.01,MsG0780036173.01,  MsG0880045572.01,MsG0880045573.01,  MsG0280008349.01,MsG0380017684.01,  MsG0780041688.01,MsG0280008350.01,  MsG0380017683.01,MsG0280008357.01,  MsG0280008352.01,MsG0680033932.01,  MsG0180003808.01,MsG0180000751.01,  MsG0380015414.01,MsG0580024333.01,  MsG0480023604.01,MsG0280010769.01,  MsG0680034513.01,MsG0680034506.01,  MsG0680034515.01,MsG0680034507.01,  MsG0680031545.01,MsG0680034516.01 |
|  |  | Glyoxylate and dicarboxylate metabolism | 2 | MsG0280011162.01,MsG0880046461.01 |
| Metabolism | Energy metabolism | Carbon fixation in photosynthetic organisms | 6 | MsG0280009541.01,  MsG0080049045.01,  MsG0180003404.01,  MsG0780041082.01,  MsG0680032095.01,  MsG0880046461.01 |
|  |  | Nitrogen metabolism | 3 | MsG0180003484.01,  MsG0080048202.01,  MsG0580027470.01 |
|  |  | Photosynthesis | 3 | MsG0380013971.01,  MsG0280007699.01,  MsG0380013971.01,  MsG0480020534.01 |
|  |  | Oxidative phosphorylation | 2 | MsG0180006211.01,  MsG0080049160.01 |
| Metabolism | Biosynthesis of other secondary metabolites | Isoflavonoid biosynthesis | 3 | MsG0380014438.01,  MsG0680030961.01,  MsG0280008072.01 |
|  |  | Flavonoid biosynthesis | 4 | MsG0480018484.01,  MsG0580030133.01,  MsG0580030135.01,  MsG0580024217.01 |
|  |  | Phenylpropanoid biosynthesis | 5 | MsG0480018484.01,  MsG0280010428.01,  MsG0380015544.01,  MsG0280007677.01,  MsG0280007684.01 |
|  |  | Stilbenoid, diarylheptanoid and gingerol biosynthesis | 1 | MsG0480018484.01 |
|  |  | Flavone and flavonol biosynthesis | 1 | MsG0680030961.01 |
|  |  | Tropane, piperidine and pyridine alkaloid biosynthesis | 1 | MsG0580024217.01 |
|  |  | Glucosinolate biosynthesis | 1 | MsG0380017247.01 |
|  |  | Caffeine metabolism | 1 | MsG0280011054.01 |
| Metabolism | Glycan biosynthesis and metabolism | Glycosylphosphatidylinositol (GPI)-anchor biosynthesis | 1 | MsG0480022746.01 |
|  |  | Glycosphingolipid biosynthesis-globo and isoglobo series | 2 | MsG0880047419.01,  MsG0780036467.01 |
|  |  | Glycosaminoglycan degradation | 2 | MsG0380018054.01,MsG0280008764.01 |
| Metabolism | Metabolism of terpenoids and polyketides | Sesquiterpenoid and triterpenoid biosynthesis | 1 | MsG0480020344.01 |
|  |  | Zeatin biosynthesis | 2 | MsG0780036054.01,  MsG0780041022.01 |
|  |  | Carotenoid biosynthesis | 2 | MsG0580029491.01,  MsG0680034868.01 |
|  |  | Diterpenoid biosynthesis | 1 | MsG0580025514.01 |
|  |  | Limonene and pinene degradation | 1 | MsG0180001068.01 |
| Metabolism | Nucleotide metabolism | Purine metabolism | 4 | MsG0280007337.01,MsG0380017404.01,  MsG0580025261.01,MsG0280011054.01 |
|  |  | Pyrimidine metabolism | 1 | MsG0480021891.01 |
| Genetic Information Processing | Transcription | Spliceosome | 9 | MsG0380016341.01,MsG0780036953.01,  MsG0480020941.01,MsG0780037877.01,  MsG0780039585.01,MsG0780036951.01,  MsG0480020939.01,MsG0880046760.01,  MsG0380012566.01 |
|  |  | Aminoacyl-tRNA biosynthesis | 1 | MsG0480018991.01 |
|  |  | Ribosome | 14 | MsG0180002438.01,MsG0480022910.01,  MsG0380017382.01,MsG0280007781.01,  MsG0580024151.01,MsG0380013547.01,  MsG0680031368.01,MsG0680031962.01,  MsG0380013949.01,MsG0180002226.01,  MsG0180004130.01,MsG0880044444.01,  MsG0180004622.01,MsG0580028107.01 |
|  |  | Nucleocytoplasmic transport | 11 | MsG0580025659.01,MsG0780041677.01,  MsG0380016341.01,MsG0080048824.01,  MsG0880042338.01,MsG0480023021.01,  MsG0180005253.01,MsG0580029519.01,  MsG0080048051.01,MsG0880041932.01,  MsG0180000068.01 |
|  |  | mRNA surveillance pathway | 4 | MsG0680035493.01,MsG0380017155.01,  MsG0180001799.01,MsG0380016341.01 |
|  |  | Ribosome biogenesis in eukaryotes | 8 | MsG0880046575.01,MsG0880046760.01,  MsG0580025659.01,MsG0880046321.01,  MsG0180004915.01,MsG0780040756.01,  MsG0780040174.01,MsG0480023627.01 |
| Genetic Information Processing | Folding, sorting and degradation | Ubiquitin mediated proteolysis | 3 | MsG0680034495.01,MsG0280006725.01,  MsG0280007827.01 |
|  |  | Sulfur relay system | 1 | MsG0180001856.01 |
|  |  | RNA degradation | 5 | MsG0480022599.01,MsG0380017155.01,  MsG0180003243.01,MsG0880043930.01,  MsG0880042581.01 |
|  |  | Protein processing in endoplasmic reticulum | 14 | MsG0680034495.01,MsG0280010728.01,  MsG0880047143.01,MsG0480022345.01,  MsG0180000472.01,MsG0480022346.01,  MsG0480022342.01,MsG0680032925.01,  MsG0780036953.01,MsG0480020941.01,  MsG0780037877.01,MsG0780039585.01,  MsG0780036951.01,MsG0480020939.01 |
|  |  | Proteasome | 2 | MsG0680035514.01,MsG0280009812.01 |
| Genetic Information Processing | Replication and repair | Homologous recombination | 1 | MsG0380015519.01 |
| Environmental Information | Signal transduction | MAPK signaling pathway - plant | 7 | MsG0880045572.01,MsG0880045573.01,  MsG0180002924.01,MsG0880047171.01,  MsG0280006550.01,MsG0280006918.01,  MsG0280006919.01 |
|  |  | Phosphatidylinositol signaling system | 3 | MsG0880046327.01,MsG0880046518.01,  MsG0880047171.01 |
|  |  | Plant hormone signal transduction | 10 | MsG0880042986.01,MsG0880044688.01,  MsG0180003906.01,MsG0480022512.01,  MsG0380017607.01,MsG0480019218.01,  MsG0680035909.01,MsG0280006550.01,  MsG0180002924.01,MsG0880047288.01 |
| Environmental Information Processing | Membrane transport | ABC transporters | 9 | MsG0580025586.01,MsG0180005151.01,  MsG0180005298.01,MsG0780040804.01,  MsG0880042368.01,MsG0680030677.01,  MsG0880043598.01,MsG0680030746.01,  MsG0480023630.01 |
| Cellular Processes | Transport and catabolism | Endocytosis | 7 | MsG0380014276.01,MsG0780036953.01,  MsG0480020941.01,MsG0780037877.01,  MsG0780039585.01,MsG0780036951.01,  MsG0480020939.01 |
|  |  | Peroxisome | 1 | MsG0280011054.01 |
| Organismal Systems | Environmental adaptation | Plant-pathogen interaction | 13 | MsG0280009139.01,MsG0480021599.01,  MsG0480021600.01,MsG0380014410.01,  MsG0380012035.01,MsG0880047171.01,  MsG0680032925.01,MsG0180005142.01,MsG0880043020.01,  MsG0680034814.01,  MsG0280006550.01,MsG0280006918.01,MsG0280006919.01 |
|  |  | Circadian rhythm - plant | 1 | MsG0580024217.01 |
